# Addressing gaps in pediatric resident education on the management of intestinal failure in the United States: Creation and implementation of a targeted curriculum

**DOI:** 10.1016/j.intf.2026.100368

**Published:** 2026-04-11

**Authors:** Sirine Belaid, Maeve Reidy, Kimberly Ackerman, Tracey Campagna, Sarah Fox, Feras Alissa, Jeffrey Rudolph, Vikram Raghu

**Affiliations:** a Department of Pediatrics, University of Pittsburgh School of Medicine, Pittsburgh, PA, United States; b Division of Pediatric Gastroenterology, UPMC Children’s Hospital of Pittsburgh, Pittsburgh, PA, United States

**Keywords:** Intestinal failure, Total parenteral nutrition, Medical education, Curriculum development

## Abstract

**Introduction:**

Pediatric residents at our institution manage patients with intestinal failure (IF), but report associated anxiety due to clinical inexperience and lack of formal education in this field. This pilot project aimed to identify knowledge gaps and address them through a targeted educational intervention.

**Methods:**

A needs assessment survey was distributed to all pediatric residents via QR codes and email. The survey included Likert-scale, multiple-choice, open-ended, and rating questions to assess confidence in IF-related care. Survey results informed a targeted curriculum that addressed key deficit areas through an in-person lecture, workshops, and a reference guide aligned with residents’ preferred learning formats. A post-intervention survey, modeled after the initial assessment, was distributed five months later to evaluate the curriculum’s impact.

**Results:**

Of the 131 residents, 40 (31%) completed the initial survey. Residents reported lowest confidence (<4/10) in calculating and ordering home Total Parenteral Nutrition (TPN) and TPN-like fluids, understanding remaining anatomy, managing central access loss and poor catheter blood flow, identifying signs of D-lactic acidosis and small intestinal bacterial overgrowth (SIBO), and ensuring proper stoma care. Conversely, they felt more confident managing feeding intolerance and central line-associated infections (5–6/10), and signs of shock (>7/10). Five months post-implementation, twenty residents (15%) completed the post-intervention survey, showing improved confidence in managing IF patients, particularly in TPN management (p = 0.0083) and enteral/stoma care (p = 0.0393). Interest in IF education increased from 64% to 85%.

**Conclusion:**

The needs assessment highlighted critical gaps in resident IF education. A targeted, resident-informed curriculum improved confidence and engagement. Next step is national dissemination.

## Introduction

Chronic intestinal failure (IF), mainly caused by short bowel syndrome (SBS), affects approximately 75 per million individuals—a rate that has remained stable over the past decade [1], [2]. With a growing patient population, improved survival rates, and declining transplant needs, the demand for healthcare providers trained in managing IF/SBS continues to rise [2], [3], [4].

However, there is a significant gap in education and provider preparedness to manage these complex patients [2], [3]. Pediatric residency programs lack a standardized curriculum on IF/SBS [5], [6]. As a result, many primary care providers and specialists—particularly in emergency medicine (EM) and gastroenterology—are not adequately trained to manage the acute and complex needs of these patients [7], [8]. This is especially concerning when patients present with complications such as central line–associated infections or septic shock, where timely and knowledgeable intervention is critical.

Decreased interest in pediatric residencies may contribute to a shortage of pediatric gastroenterologists and intestinal rehab (IR) specialists, further compounding the issue, as those currently in practice may lack sufficient training in IF/SBS care [2], [7], [9], [10], [11]. With projections showing a continued decline in the pediatric workforce, there is an urgent need to improve training at the residency level [9], [13].

At our institution—an internationally recognized center for the surgical and medical management of children with SBS—pediatric residents care for these patients during inpatient, EM, and intensive care unit rotations, as well as through multidisciplinary clinic visits. Despite this clinical exposure, the absence of a standardized IF/SBS curriculum leaves residents inadequately prepared to manage the complex care needs of this population. This project aimed to identify knowledge gaps among pediatric residents at our institution regarding IF/SBS management and to develop a targeted curriculum to address these deficits.

## Methods

At the start of the academic year in July 2024, an initial needs assessment was conducted using an online survey created in Qualtrics (Appendix A). The survey was distributed to pediatric residents (PGY 1–3) at our institution by sharing QR codes at the end of a noon lecture, followed by reminder emails to all residents. Participants were given one month to respond. The survey included Likert-scale, multiple-choice, open-ended, and rating questions to assess self-reported confidence in managing patients with IF and SBS. Non-clinical staff reviewed the survey for face validity, and IR specialists reviewed it for content validity. Participation was voluntary, anonymous, and approved as exempt by the institutional review board (STUDY #: 24020040).

A targeted curriculum was developed after survey results revealed gaps in resident confidence. The curriculum was implemented in January 2025 using educational resources preferred by the residents. Content development was informed by an extensive literature review and by curated materials provided by ostomy nurses and the IR team.

The curriculum was launched with a 45-minute in-person lecture (Appendix B), delivered by a third-year pediatric gastroenterology fellow during a noon conference. Key knowledge deficits identified in the needs assessment— including post-surgical anatomy and related complications, central line care and complications, recognition and management of central line–associated bloodstream infections (CLABSI), stoma care, and enteral tube complications—were addressed. The lecture was recorded and made available to residents who could not attend in person.

In addition, weekly hands-on Total Parenteral Nutrition (TPN) workshops (Appendix C) were led by the same third-year pediatric gastroenterology fellow for PGY-1 and PGY-2 residents rotating on the gastroenterology service. During these sessions, residents worked through structured, case-based scenarios to practice calculating and ordering TPN and TPN-like intravenous (IV) fluids. These solutions, administered centrally or peripherally, contain dextrose and major electrolytes and are designed to approximate the contents of patients’ home TPN.

To supplement the in-person sessions, a two-page quick reference guide (Appendix D) was developed. This resource outlined common and emergent issues related to IF care in a bullet-point format and was designed for inclusion in the resident survival guide to improve accessibility.

To evaluate the effectiveness of the curriculum, a post-intervention survey (Appendix E) was administered five months later, closely modeled on the initial assessment to allow for direct comparison. The survey was distributed in person via QR code and by email to encourage maximum participation. Ordinal data were analyzed using the Mann–Whitney *U* test and the Kruskal–Wallis test, with a significance threshold set at an alpha level of 0.05.

## Results

### Rated confidence levels from the needs assessment survey

Among the 131 pediatric residents across all PGY levels assigned to the IR service during their training, we collected survey responses from 40 residents (31%) between July 26 and August 11, 2024. Half of the respondents had already completed a rotation on the IR service.

**Fig. 1:**
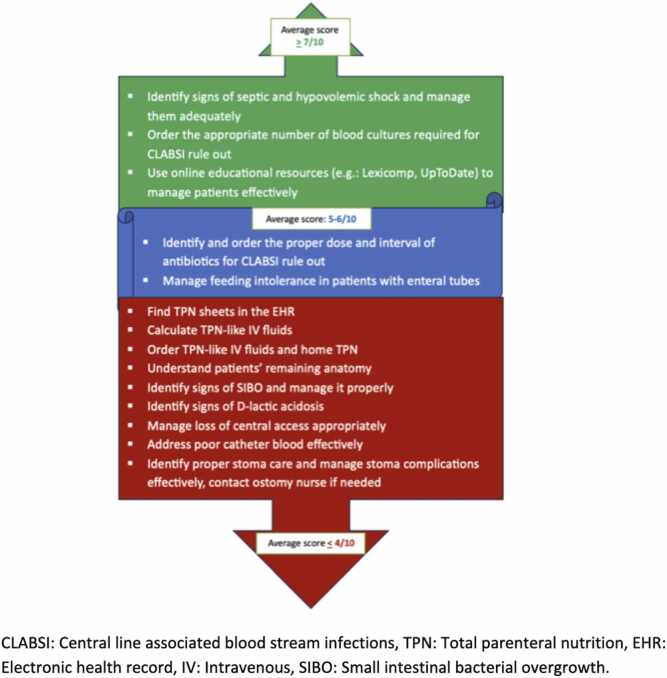
Average rating scores of pediatric residents on the most common and emergent topics related to patients with intestinal failure (IF) and short bowel syndrome (SBS).

Residents reported the lowest confidence in calculating and ordering home TPN and TPN-like IV fluids, understanding patients’ remaining anatomy, managing central access loss, addressing poor catheter blood flow, identifying signs of D-lactic acidosis and small intestinal bacterial overgrowth (SIBO), managing SIBO, and providing proper stoma care (average confidence rating of <4/10). Conversely, residents expressed slightly more confidence in managing feeding intolerance and CLABSI (average ratings of 5–6/10) and reported high confidence in identifying and managing signs of septic and hypovolemic shock (>7/10). These findings are summarized in Fig. 1.

### Qualitative survey results

A few residents shared additional details about their experiences rotating on the IR service overnight. In their comments, they emphasized feeling unprepared and inexperienced in calculating and ordering TPN-like IV fluids, ordering the appropriate number of blood cultures for CLABSI rule out, and managing prolapsed ostomy.

### Educational value and preferred resources

Additionally, 64% of the residents agreed – including 25% who strongly agreed – that managing IF patients is educationally valuable. Residents identified their preferred educational resources as a quick reference survival guide and simulated sessions, with average ratings of 8 and 6.8 out of 10, respectively. These were followed by in-person lectures (5.6/10) and lastly online modules (3.5/10). Finally, 98% of the respondents agreed – including 81% who strongly agreed – that an admission order set would be beneficial for IF patients.

### Curriculum intervention

Based on the results of the needs assessment survey, we developed a curricular intervention to address the topics residents felt least confident in. We launched the curriculum with an in-person lecture in early January 2025, which was attended by 26 residents (20%). The lecture targeted key areas where knowledge gaps had been identified: understanding patients’ remaining anatomy and associated complications, managing CLABSI, and addressing central line, enteral tube, and stoma complications. In addition, weekly hands-on TPN workshops were conducted for pediatric residents (PGY-1 and PGY-2) rotating on the gastroenterology service. Over the course of the intervention, 34 residents (40% of PGY 1 – 2) participated. These workshops provided practical experience in calculating and ordering TPN-like IV fluids and ordering home TPN. Finally, we created a two-page survival guide summarizing the most common and emergent issues encountered on the IR service in a bullet-point format. Topics included calculating and ordering TPN-like IV fluids, managing CLABSI, handling loss of central access, and troubleshooting poor blood return from catheters.

### Post-intervention survey

At the end of May 2025—five months after we implemented the curriculum—we distributed a post-intervention survey containing questions similar to those in the initial assessment to allow for direct comparison. A total of 20 residents (15%) completed the survey. Of those, the majority had previously rotated on the IR service; 5 attended the lecture, and 14 participated in the TPN workshops.

Residents reported significantly higher confidence ratings across all tasks related to IF care following the intervention (p < 0.05), with the exception of CLABSI management, as shown in Table 1.

**Table 1:** Pre- and post-intervention comparison of resident confidence levels (1–10 scale) across 23 tasks related to the care of patients with intestinal failure and short bowel syndrome.

**Questions**	**Tasks**	**Pre-Intervention Scores**^a^		**Post-Intervention Scores**^a^		***p***
		**Median**	**Mean (SD)**	**Median**	**Mean (SD)**	
Question 1	**Finding TPN sheets on Cerner**	2	2.64 (3.12)	8	7.5 (3.09)	**.0000**
Question 2	**Calculate TPN-like fluids**	1	1.77 (2.06)	6	5.95 (3.15)	**.0000**
Question 3	**Order TPN-like fluids on Cerner**	2	3.21 (3.26)	8.5	7.15 (3.17)	**.0002**
Question 4	**Order home TPN**	1	2.79 (3.25)	8	7.4 (2.74)	**.0000**
Question 5	Identify proper antibiotics for CLABSI rule out	5	5.23 (2.92)	7	6.2 (2.55)	.2550
Question 6	Order proper dose of antibiotics for CLABSI rule out	6	5.28 (3.01)	7	6.75 (2.55)	.0732
Question 7	Order proper interval of antibiotics for CLABSI rule out	6	5.26 (2.87)	7	6.5 (2.74)	.1065
Question 8	Order appropriate blood cultures required for CLABSI rule out	7	6.08 (3.26)	7.5	7.2 (2.48)	.2481
Question 9	**Identify proper steps when central access is lost**	3	3.05 (2.71)	6	5.9 (2.59)	**.0004**
Question 10	**Manage poor blood return from central line**	3	3.51 (3.02)	5.5	5.6 (2.35)	**.0082**
Question 11	**Identify signs of hypovolemic shock**	7	6.31 (2.70)	8	7.6 (2.44)	**.0497**
Question 12	**Manage hypovolemic shock**	6	5.62 (2.80)	8	7.55 (2.48)	**.0028**
Question 13	**Identify signs of septic shock**	7	6.31 (2.50)	8	7.65 (2.25)	**.0221**
Question 14	**Manage septic shock**	6	5.90 (2.35)	8	7.7 (2.11)	**.0040**
Question 15	**Identify appropriate steps for feeding intolerance**	5	4.26 (2.89)	7	6.35 (2.32)	**.0067**
Question 16	**Identify signs of D-lactic acidosis**	1	1.74 (1.83)	5	4.4 (2.70)	**.0002**
Question 17	**Identify signs of bacterial overgrowth**	1	1.95 (1.86)	5	4.35 (1.98)	**.0001**
Question 18	**Manage suspected bacterial overgrowth**	1	1.38 (1.55)	4.5	3.9 (2.17)	**.0000**
Question 19	**Effectively understand their remaining bowel anatomy**	2	2.15 (2.21)	5	4.45 (1.70)	**.0001**
Question 20	**Identify barrier cream for enteral/stoma care**	2	2.72 (2.58)	4	4.35 (1.81)	**.0039**
Question 21	Order barrier cream for enteral/stoma care	4	3.95 (3.07)	5	5.4 (1.98)	.0600
Question 22	**Contact colostomy nurse to assist me in patient care**	4	4.13 (3.23)	5	5.9 (2.85)	**.0390**
Question 23	**Use educational resources to assist me in patient care**	8	7.54 (2.40)	9	8.75 (1.37)	**.0455**

Abbreviations: SD: Standard deviation, TPN: Total parenteral nutrition, CLABSI: Central line associated blood stream infections.

a Rated on a 10-point scale (1 = Not confident, 10 = Very confident).

**Fig. 2:**
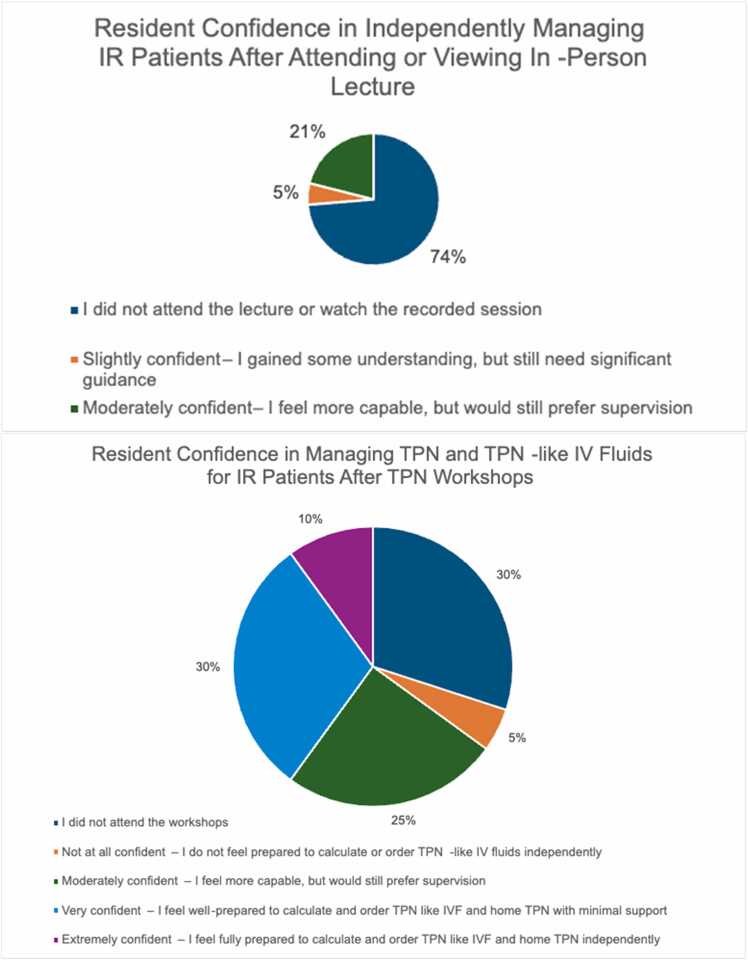
Self-reported confidence in managing intestinal rehabilitation patients among residents who completed the post-intervention survey after the in-person lecture (n = 5) and TPN workshop (n = 14). Abbreviations: IR: Intestinal rehab; TPN: Total parenteral nutrition; IV: Intravenous.

The five residents who attended or watched the recorded lecture showed significantly improved confidence in managing enteral and stoma care (p = 0.039). They also reported slightly higher, though not statistically significant scores in identifying appropriate antibiotics for CLABSI rule-out and managing poor catheter blood return, compared to those who did not attend the lecture (Table 2a). Four out of five residents expressed feeling moderately more confident in managing patients with IF, as shown in Fig. 2. Residents responded positively to the lecture and requested that it be offered regularly as a refresher course.

**Table 2a:** Comparison of resident confidence levels (1–10 scale) across tasks discussed during the in-person lecture between attendees and non-attendees.

**Questions**	**Tasks**	**Lecture**^a^		**No Lecture**^a^		***p***
		**Median**	**Mean (SD)**	**Median**	**Mean (SD)**	
Question 5	Identify proper antibiotics for CLABSI rule out	7	7 (3.08)	6	5.93 (2.40)	.4066
Question 6	Order proper dose of antibiotics for CLABSI rule out	7	7.8 (1.64)	7	6.4 (2.75)	.4774
Question 7	Order proper interval of antibiotics for CLABSI rule out	7	6.6 (3.21)	7	6.47 (2.70)	.9251
Question 8	Order appropriate blood cultures required for CLABSI rule out	9	7.4 (3.44)	7	7.13 (2.23)	.6669
Question 9	Identify proper steps when central access is lost	5	5.4 (2.97)	6	6.07 (2.55)	.5157
Question 10	Manage poor blood return from central line	8	7 (1.73)	5	5.13 (2.39)	.1188
Question 15	Identify appropriate steps for feeding intolerance	7	6.8 (1.30)	7	6.2 (2.60)	.8709
Question 16	Identify signs of D-lactic acidosis	3	3.6 (1.82)	5	4.67 (2.94)	.5368
Question 17	Identify signs of bacterial overgrowth	5	4 (1.41)	5	4.67 (2.17)	.6356
Question 18	Manage suspected bacterial overgrowth	5	3.6 (2.41)	4	4 (2.17)	.6976
Question 19	Effectively understand their remaining bowel anatomy	5	5.4 (1.14)	5	4.13 (1.77)	.2185
Question 20	**Identify barrier cream for enteral/stoma care**	5	5.8 (1.64)	4	3.87 (1.64)	**.0393**
Question 21	Order barrier cream for enteral/stoma care	8	6.6 (1.95)	5	5 (1.89)	.1202
Question 22	Contact colostomy nurse to assist me in patient care	10	7.6 (3.29)	5	5.33 (2.55)	.2039

Abbreviations: SD: Standard deviation, CLABSI: Central line associated blood stream infections.

a Rated on a 10-point scale (1 = Not confident, 10 = Very confident).

Residents who participated in the TPN workshops demonstrated significantly higher confidence in calculating TPN-like IV fluids (p = 0.008). They also showed higher average ratings in locating TPN order sheets and ordering TPN-like IV fluids compared to those who did not attend, though these differences were not statistically significant (Table 2b). Thirteen out of fourteen residents expressed improved confidence in managing TPN and TPN-like IV fluids after the intervention, as reflected in Fig. 2. Residents provided enthusiastic feedback, describing the workshops as informative, practical, and highly relevant to their clinical experience with IF patients. One resident remarked, *“The 1:1 teaching was so helpful and informative. I liked having case-based learning with very practical examples.”* Another stated, *“The in-person, small group, practical and hands-on workshop greatly increased my confidence.”* Other residents also noted that the skills gained were applicable beyond IF care, with one commenting, *“The skills are translatable beyond just the IR service, and I feel more confident caring for IR kids knowing that I understand their nutrition.”*

**Table 2b:** Comparison of resident confidence levels (1–10 scale) in managing TPN-related tasks between TPN workshop attendees and non-attendees.

**Questions**	**Tasks**	**TPN workshops**^a^		**No TPN workshops**^a^		***p***
		**Median**	**Mean (SD)**	**Median**	**Mean (SD)**	
Question 1	Finding TPN sheets on Cerner	9	7.64 (3.10)	7.5	7.17 (3.31)	.7121
Question 2	**Calculate TPN-like fluids**	**8**	7 (3.01)	4.5	3.5 (1.97)	**.0083**
Question 3	Order TPN-like fluids on Cerner	9	7.29 (3.17)	7.5	6.83 (3.43)	.8211
Question 4	Order home TPN	8	7.36 (2.76)	8	7.5 (2.95)	.9622

Abbreviations: TPN: Total parenteral nutrition, SD: Standard deviation

a Rated on a 10-point scale (1 = Not confident, 10 = Very confident).

**Fig. 3:**
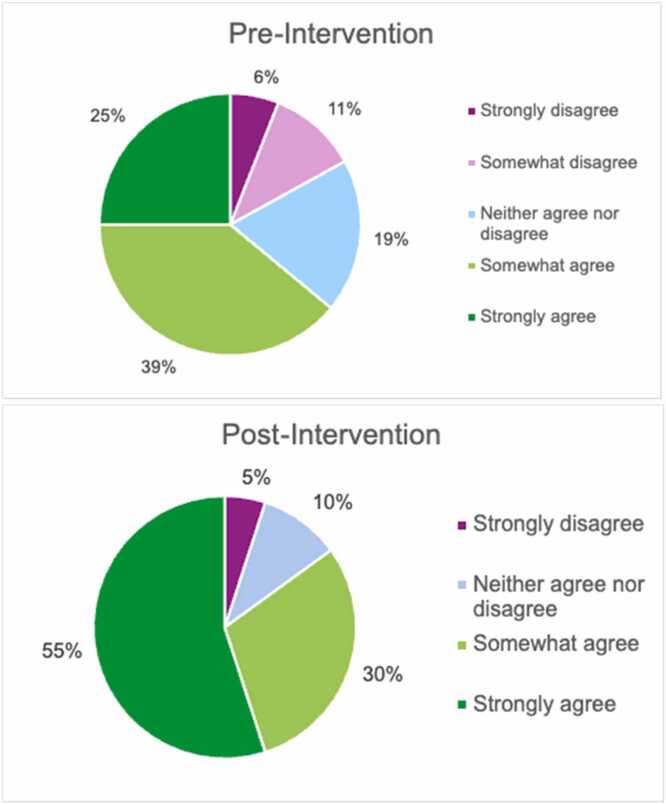
Comparison of pre-intervention (n = 40) and post-intervention (n = 20) pediatric residents’ perceptions of the educational value of managing patients with intestinal failure in pediatric training.

As illustrated in Fig. 3, 85% of residents agreed—with 55% strongly agreeing—that managing IF patients is educationally valuable. This represented a 21% increase in agreement following the intervention.

## Discussion

Our needs assessment survey clearly demonstrated both a strong interest among pediatric residents in learning how to manage patients with SBS and IF, as well as significant knowledge gaps in key areas. Residents expressed the lowest confidence in topics such as TPN management, central line-associated complications, stoma care, and enteral tube management. In response, we developed a curriculum specifically targeting these deficits.

Following the implementation of the 5-month pilot curriculum, post-intervention survey results showed improved resident confidence in managing patients with SBS/IF—particularly in TPN management, enteral tube care, and stoma care.

This is a unique curriculum designed specifically for pediatric residents to address the management of patients with SBS and IF. While other institutions have identified similar educational deficits, most efforts have focused on partial curricula or targeted learning for different audiences.

### Comparison with other curricula

Cincinnati Children’s Hospital Medical Center developed a gastroenterology curriculum using fellows as educators—similar to our approach [5]. Their content was delivered through case-based discussions, short didactic sessions, self-directed learning cases, and topic reviews. Their curriculum evolved based on a needs assessment survey distributed to recent residency graduates now working in general pediatrics. The survey revealed average scores in nutrition topics and the lowest confidence in managing enteral tube care. After implementation, a modest improvement in knowledge was observed (pre- vs. post-test MCQ scores improved from 20.5 to 22.6). Six months post-implementation, residents requested more SBS content due to frequent clinical exposure—an observation similarly noted among our residents on the IR service.

Children’s Hospital of Alabama conducted a comprehensive needs assessment survey asking pediatric residents about both knowledge and self-assessed comfort levels in TPN initiation and progression, enteral nutrition and access, and recognition of nutritional deficiencies [14]. Dietitians were also surveyed to identify perceived needs of pediatric residents, with 9 out of 10 agreeing that using enteral and parenteral nutrition ordering systems was the most pressing gap. Their curriculum included monthly simulations focused on managing nutritional deficiencies during TPN shortages. The simulation received positive feedback, with 96% of residents agreeing that the experience would improve their clinical performance.

Ann & Robert H. Lurie Children’s Hospital of Chicago created a self-directed, web-based module for pediatric hospitalists focused on caring for medically complex children with central lines [15]. The content addressed types of central lines and complications, including CLABSI, thrombosis, and catheter patency—an effort that aligns with the central line-related gaps our own residents reported.

Despite the increasing need for education in this area, there is a notable lack of updated resources from the North American Society for Pediatric Gastroenterology, Hepatology, and Nutrition (NASPGHAN) [16], [17]. The available curricular materials consist primarily of PowerPoint slides, guidelines, and video series. While these resources briefly cover TPN components, they lack practical guidance on ordering TPN, calculating TPN-like IV fluids, and managing central line–associated complications. Similarly, enteral tube complications are minimally addressed, and stoma care is not mentioned at all. When SBS is referenced, the focus is limited to its etiology, without further discussion on the importance of understanding the patient’s remaining anatomy or functional adaptation.

### Resident preferred educational resources

Our needs assessment also asked residents about their preferred learning methods. Interestingly, the majority did not prefer online modules. This could be attributed to online learning fatigue, particularly in the wake of the COVID-19 pandemic, which accelerated the shift to virtual education. The reduced interactivity of online modules and the fact that they often require personal time may further explain their unpopularity. In contrast, in-person lectures, quick-reference guides, and simulation-based workshops were preferred, likely due to their hands-on and interactive nature. These findings align with prior research highlighting the value of adult learning principles, particularly the emphasis on interactive, applied learning experiences [5], [14], [18].

Another notable finding was that nearly all residents expressed a desire for an admission order set for IR patients—underscoring an opportunity to improve clinical efficiency and reduce errors during transitions of care.

### The growing need for SBS/IF education

With improved survival and a decreased need for intestinal transplants, the number of pediatric IF/SBS patients is expected to increase. However, fewer trainees are entering primary care or pediatric subspecialties, raising concern about the future availability of providers equipped to manage this complex population [2], [3], [8].

Even among current providers, training gaps persist. Due to limited nutrition-related content in residency curricula, studies have shown that pediatric trainees often lack sufficient knowledge in key areas of nutrition [6], [18] and receive inadequate guidance in managing patients with IF/SBS [5]. Similarly, pediatric gastroenterology providers and providers actively managing IF patients have also reported feeling inadequately prepared [2], [7], [10], which has led to the development of initiatives such as Learn Intestinal Failure Tele-ECHO [11], [12]. This program aims to educate multidisciplinary teams, including GI providers.

Our curriculum takes a proactive approach by targeting pediatric residents earlier in their training to strengthen foundational knowledge and better prepare them for the complexities of managing IF/SBS patients. Better-educated and trained residents may also develop a stronger interest in IR, ultimately contributing to a more capable and committed workforce.

As these patients increasingly present to outpatient clinics, emergency departments, and inpatient settings, it is essential that frontline providers—including pediatric residents—receive appropriate training. Clinical exposure alone is insufficient. Our data shows that despite rotating on IR services, residents still reported substantial gaps in confidence and knowledge, underscoring the need for structured education—an approach supported by the majority of residents.

Because these patients are medically complex—often requiring central lines, feeding tubes, and stomas—they offer a valuable learning opportunity for building competency in managing the growing population of children with medical complexity [19], [20]. This curriculum aims to equip trainees not only to care for patients with IF/SBS, but also to confidently manage any medically complex patients they may encounter throughout their careers.

### Strengths

Key strengths of this project include leveraging a needs assessment to identify targeted knowledge gaps, incorporating residents’ preferred learning methods to enhance curriculum engagement, and implementing a pilot with both pre- and post-intervention evaluation. Despite the time gap between the intervention and survey completion, residents still demonstrated strong results, suggesting retained understanding and possible competence. Leveraging fellows as educators allowed for sustainability and faculty time efficiency while also enhancing fellow teaching skills and leadership. Another notable strength was that noon lectures and TPN workshops were directly incorporated into resident education during their GI rotations, improving access and engagement.

### Limitations

There are several limitations to consider. Voluntary participation may have lowered the response rate and introduced response bias. Respondents were likely overrepresented by first-year residents, who were more likely to attend in-person sessions where the survey was distributed, as well as by residents with a particular interest in the topic. Survey fatigue may have further limited participation, resulting in a small and potentially biased sample. Post-intervention results may have been influenced by confounding factors, such as residents gaining additional exposure through IR elective outpatient rotations or informal learning experiences over the course of their residency. Due to incomplete response overlap and the anonymous nature of the surveys, matched pre- and post-intervention survey scores were not available. Consequently, we compared aggregate scores from all residents who completed the surveys at each time point. Additionally, attendance at the in-person lecture was limited, and we were unable to verify whether absent residents accessed the recorded materials. The low attendance may have been due to the January timing, during a winter patient surge, when many residents were likely busy with clinical duties. Only five of the twenty residents who attended the lecture completed the post-lecture survey, limiting statistical power and likely contributing to the absence of significant differences between the two groups. Furthermore, due to time constraints, SIBO and D-lactic acidosis were not covered in detail during the lecture, which may have further contributed to the lack of observed differences between attendees and non-attendees. Due to logistical constraints, it was not feasible for the pediatric residency program to convene all residents—or even each PGY level separately—for the TPN workshops. As a result, these workshops were primarily delivered to residents rotating on the gastroenterology service, typically first- and second-year trainees, which may limit the generalizability of our findings to third-year residents. The quick reference guide was added after survey completion, so post-intervention results did not reflect its impact. Including it earlier may have yielded stronger outcomes. This intervention was conducted at a single institution, and thus findings may not be generalizable to all pediatric residency programs. Long-term sustainability remains uncertain, as continued implementation requires dedicated time and personnel.

## Conclusion

Our needs assessment revealed notable knowledge gaps and low confidence among pediatric residents in managing SBS/IF, particularly in TPN management, central line–associated complications, stoma care, and enteral tube management. Following implementation of a targeted 5-month curriculum, residents demonstrated improved confidence across these domains, with the greatest gains in TPN management, enteral tube care, and stoma care, along with a growing interest in IF education.

Based on participant feedback, we plan to iteratively refine the curriculum to ensure it remains relevant, learner-centric, and responsive to the evolving needs of trainees. The next step is to implement the revised curriculum institution-wide over a full academic year, allowing for more comprehensive evaluation and continued improvement. Our long-term goal is national dissemination and advocacy for its integration into pediatric residency and pediatric gastroenterology fellowship training standards, including potential updates to ABP guidelines.

Recognizing that not all residency programs have access to patients with IF/SBS, we encourage trainees at smaller institutions to pursue away rotations for hands-on experience. The curriculum is designed to be highly adaptable and reproducible—lectures can be delivered by IR or gastroenterology providers, the survival guide can be distributed electronically, and TPN workshops can be supplemented with recorded materials when staffing is limited. While the original curriculum was intended for residents, we believe the content can be adapted for trainees at all levels, including pediatric and adult gastroenterology fellows, as well as junior faculty interested in managing intestinal failure.

## CRediT authorship contribution statement

**Jeffrey Rudolph:** Writing – review & editing, Validation, Supervision, Resources, Conceptualization. **Feras Alissa:** Writing – review & editing, Validation, Supervision, Resources, Conceptualization. **Sarah Fox:** Writing – review & editing, Validation, Resources. **Tracey Campagna:** Writing – review & editing, Validation, Resources. **Kimberly Ackerman:** Writing – review & editing, Validation, Resources. **Maeve Reidy:** Writing – review & editing, Validation, Project administration, Methodology, Conceptualization. **Sirine Belaid:** Writing – review & editing, Writing – original draft, Validation, Supervision, Resources, Project administration, Methodology, Investigation, Formal analysis, Data curation, Conceptualization. **Vikram Raghu:** Writing – review & editing, Validation, Supervision, Resources, Methodology, Investigation, Formal analysis, Conceptualization.

## Patients consent

Not applicable.

## Patients Consent

Not applicable.

## Ethical clearance

Not required.

## Declaration of Generative AI and AI-assisted technologies in the writing process

During the preparation of this manuscript, the authors used ChatGPT to assist in identifying typographical and grammatical errors. After using this tool/service, the authors reviewed and edited the content as needed and take full responsibility for the content of the published article.

## Financial support

No funding sources to disclose.

## Funding

This research did not receive any specific grant from funding agencies in the public, commercial or not-for-profit sectors.

## Appendices

A: Needs Assessment Survey.pdf

B: In-Person Lecture.pptx

C: TPN Workshops.pptx

D: Quick Reference Guide.docx

E: Post-Intervention Survey.pdf

## Acknowledgments

The authors would like to thank the residents at the University of Pittsburgh Medical Center for their participation in this project.

## Guarantor of the article

Sirine Belaid, no conflict of interest to disclose.

## Potential competing interests

The authors report no conflicts of interest.

## Declaration of Competing Interest

The authors declare that they have no known competing financial interests or personal relationships that could have appeared to influence the work reported in this paper.
