# Supplementary material for: Addressing gaps in pediatric resident education on the management of intestinal failure in the United States: Creation and implementation of a targeted curriculum
Source: Intest Fail. 2026 Apr 11;10:100368. doi: 10.1016/j.intf.2026.100368 (PMC13092194; doi:10.1016/j.intf.2026.100368)
Supplement: Supplementary file 1 — Supplementary material [file mmc1.pdf]

Default Question Block

Rate from 1 to 10 the following items based on your level of confidence in performing the following tasks **individually** when managing ICARE patients, with 1 being not confident and 10 being very confident.

ICARE stands for Intestinal Care and Rehabilitation Center
TPN stands for Total Parenteral Nutrition
CLABSI stands for Central Line-Associated Bloodstream Infection

Click to write Label 1

	0	1	2	3	4	5	6	7	8	9	10
Finding TPN sheets on Cerner											
Calculate TPN-like fluids											

Click to write Label 1

0 1 2 3 4 5 6 7 8 9 10

Order TPN-like fluids on Cerner

Order home TPN

Identify proper antibiotics for CLABSI rule out

Order proper dose of antibiotics for CLABSI rule out

Order proper interval of antibiotics for CLABSI rule out

Order appropriate blood cultures required for CLABSI rule out

Click to write Label 1

	0	1	2	3	4	5	6	7	8	9	10	
Identify proper steps when central access is lost												
Manage poor blood return from central line												
Identify signs of hypovolemic shock												
Manage hypovolemic shock												
Identify signs of septic shock												
Manage septic shock												

Click to write Label 1

	0	1	2	3	4	5	6	7	8	9	10
Identify appropriate steps for feeding intolerance (with otherwise reassuring exam)											
Identify signs of D-lactic acidosis											
Identify signs of bacterial overgrowth											
Manage suspected bacterial overgrowth											
Effectively understand their remaining bowel anatomy											

Click to write Label 1

	0	1	2	3	4	5	6	7	8	9	10
Identify barrier cream for enteral/stoma care											
Order barrier cream for enteral/stoma care											
Contact ostomy nurse to assist me in patient care											
Use educational resources (such as Lexicomp, Uptodate) to assist me in patient care											

I believe pediatric residents would benefit from an *admission* order set when managing ICARE patients.

- Strongly disagree
- Somewhat disagree
- Neither agree nor disagree
- Somewhat agree
- Strongly agree

I believe it is of educational value for pediatric residents to manage ICARE patients.

- Strongly disagree
- Somewhat disagree
- Neither agree nor disagree
- Somewhat agree
- Strongly agree

Rate from 1 to 10 your *preferred* form of educational resources to manage ICARE patients, 1 being not helpful and 10 being very

helpful.

	0	1	2	3	4	5	6	7	8	9	10	
In-person lectures												
Online modules												
Laminated cards (such as PALS cards)												
Simulation sessions												

Have you rotated on ICARE (day or night) prior to taking this survey?

- Yes
- No
- I cannot recall

If you answered “Yes” to the previous question, have you encountered a particularly challenging or stressful situation while managing an ICARE patient? If so, could you kindly share the details?

Please provide the first and last letters of your **last** name so we can follow up on your progress with the post-intervention survey.

Powered by Qualtrics
