# Supplementary material for: Addressing gaps in pediatric resident education on the management of intestinal failure in the United States: Creation and implementation of a targeted curriculum
Source: Intest Fail. 2026 Apr 11;10:100368. doi: 10.1016/j.intf.2026.100368 (PMC13092194; doi:10.1016/j.intf.2026.100368)
Supplement: Supplementary file 2 — Supplementary material [file mmc2.pptx]

## Slide 1
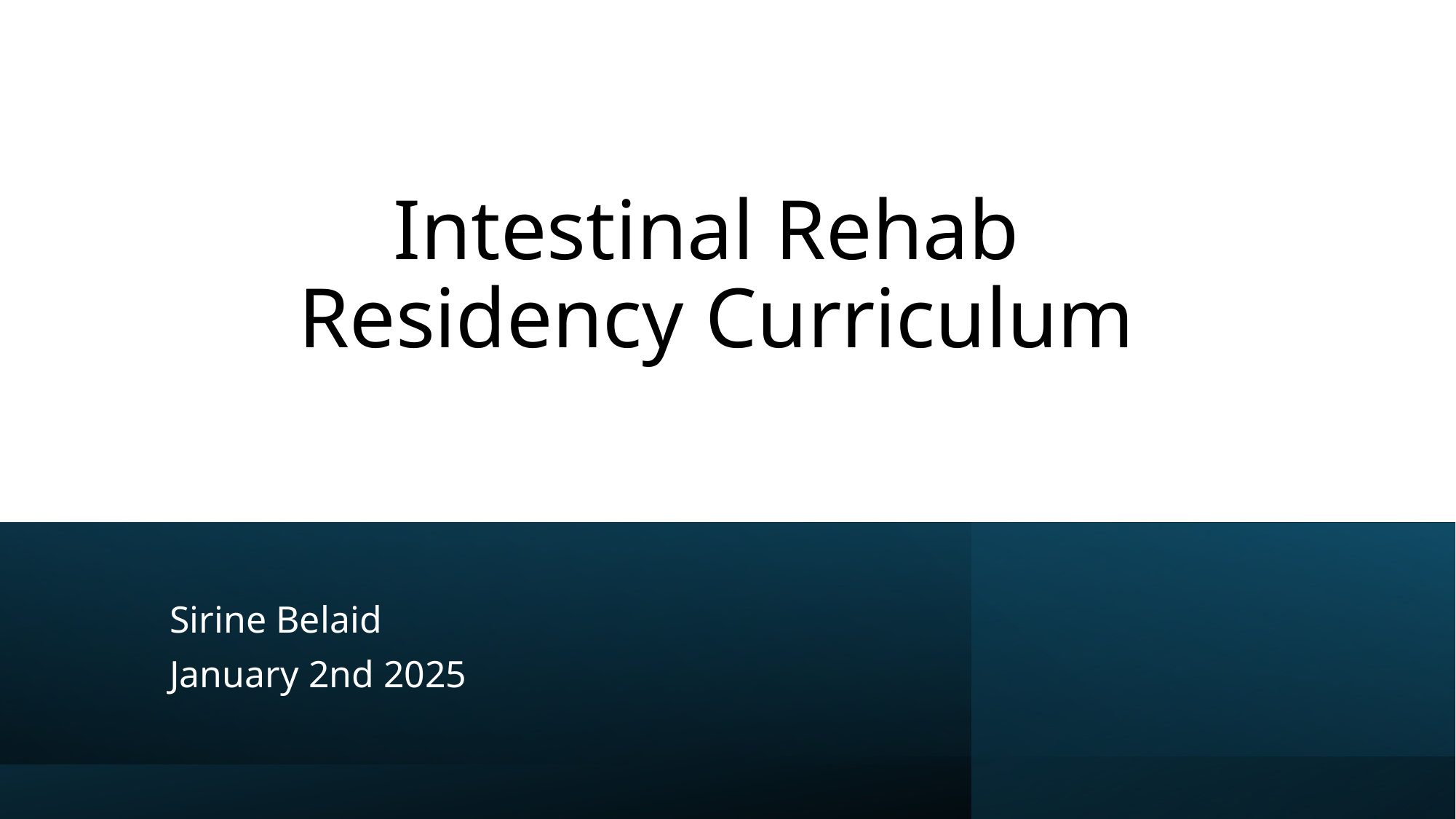

# Intestinal Rehab Residency Curriculum
Sirine Belaid
January 2nd 2025

## Slide 2
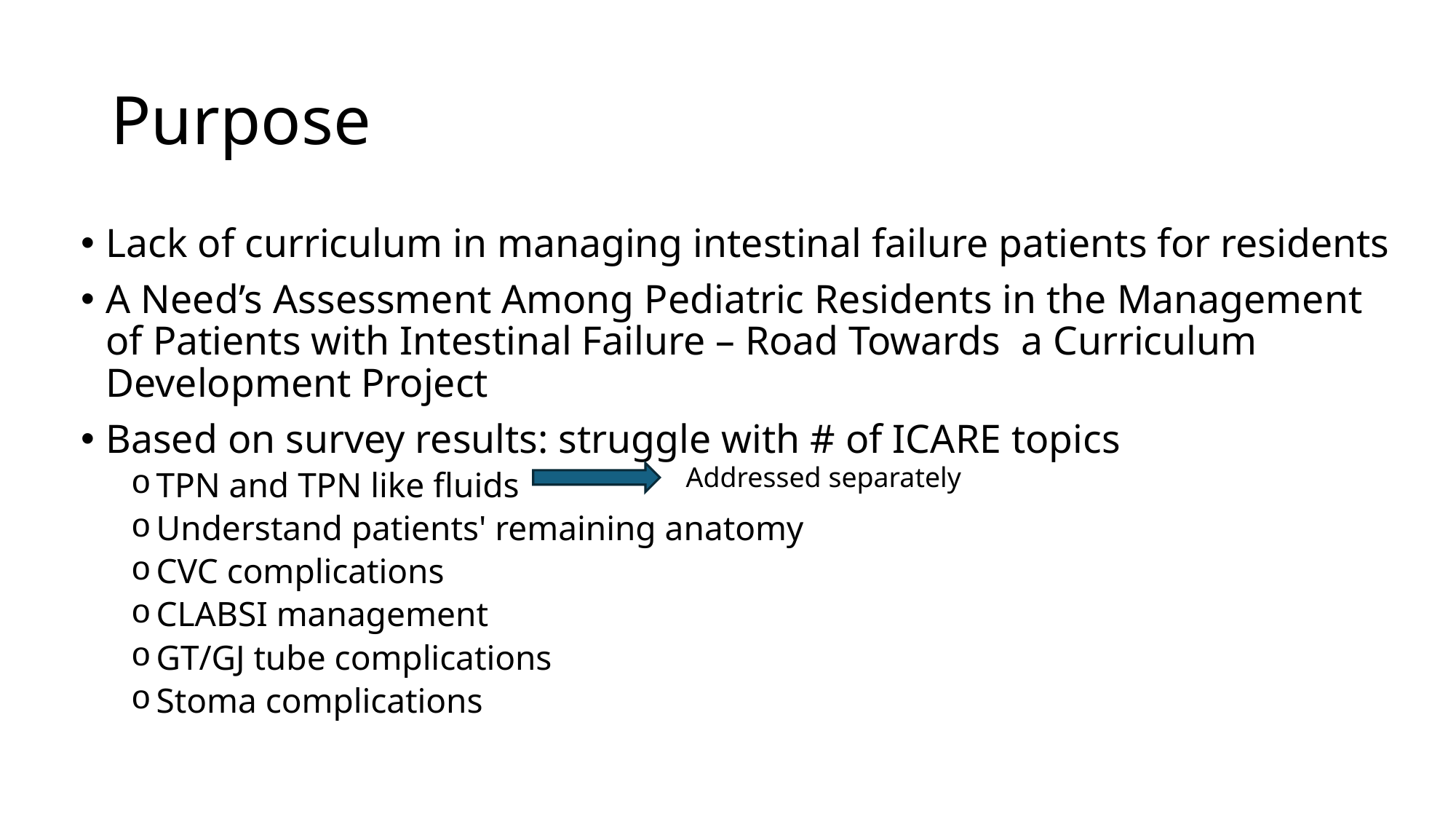

# Purpose
Lack of curriculum in managing intestinal failure patients for residents
A Need’s Assessment Among Pediatric Residents in the Management of Patients with Intestinal Failure – Road Towards  a Curriculum Development Project
Based on survey results: struggle with # of ICARE topics
TPN and TPN like fluids
Understand patients' remaining anatomy
CVC complications
CLABSI management
GT/GJ tube complications
Stoma complications
Addressed separately

## Slide 3
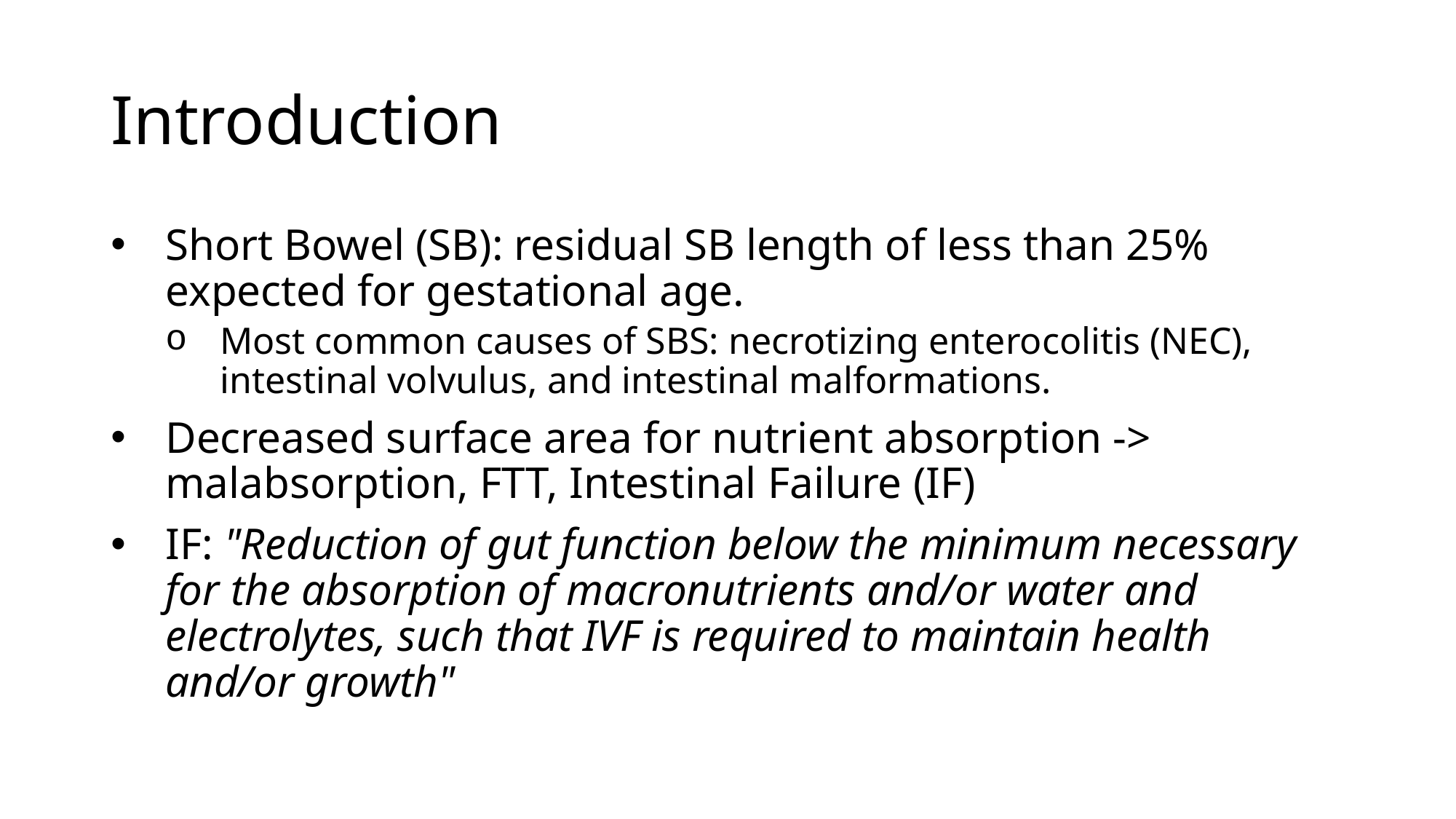

# Introduction
Short Bowel (SB): residual SB length of less than 25% expected for gestational age.
Most common causes of SBS: necrotizing enterocolitis (NEC), intestinal volvulus, and intestinal malformations.
Decreased surface area for nutrient absorption -> malabsorption, FTT, Intestinal Failure (IF)
IF: "Reduction of gut function below the minimum necessary for the absorption of macronutrients and/or water and electrolytes, such that IVF is required to maintain health and/or growth"

## Slide 4
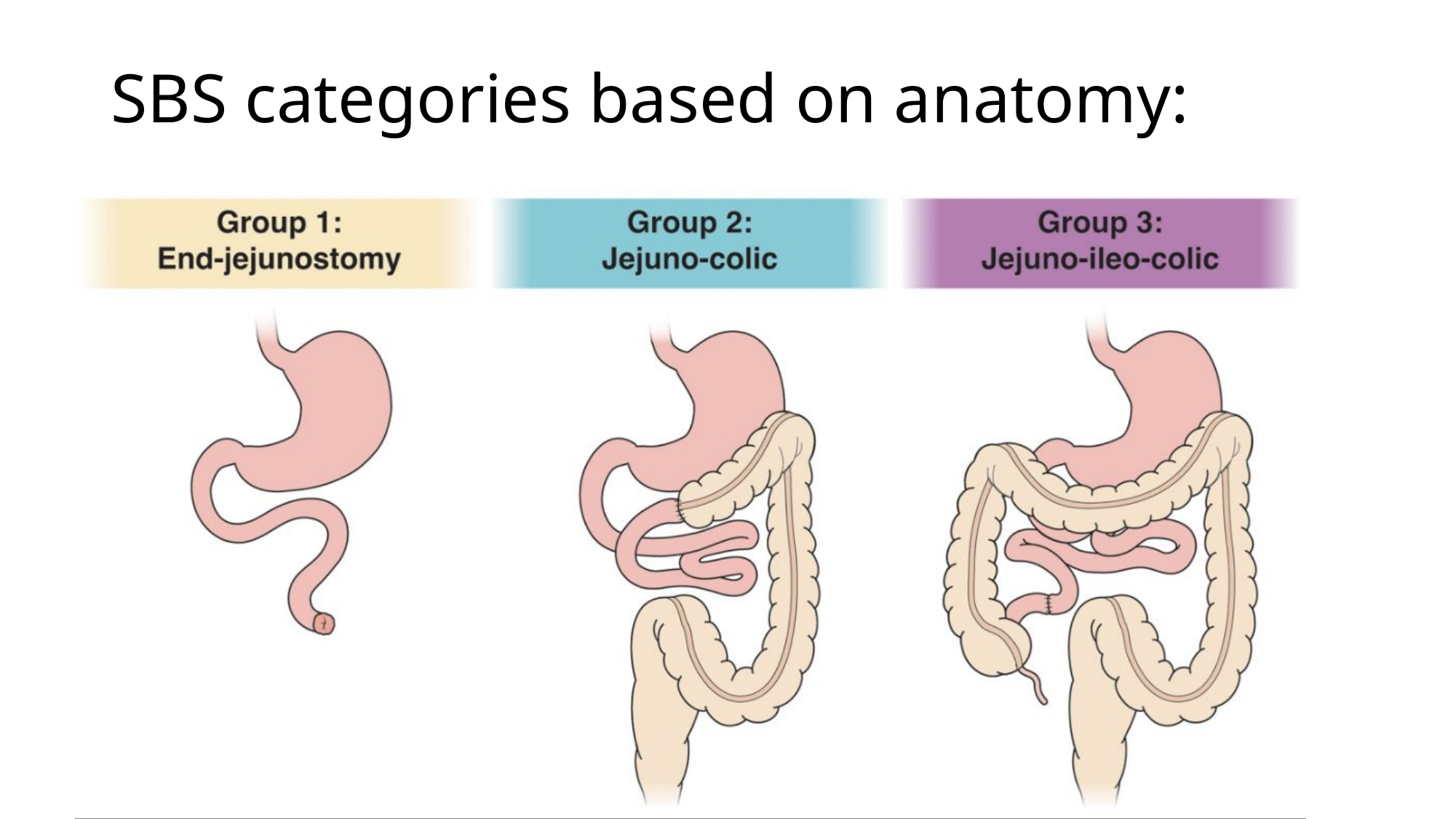

# SBS categories based on anatomy:

## Slide 5
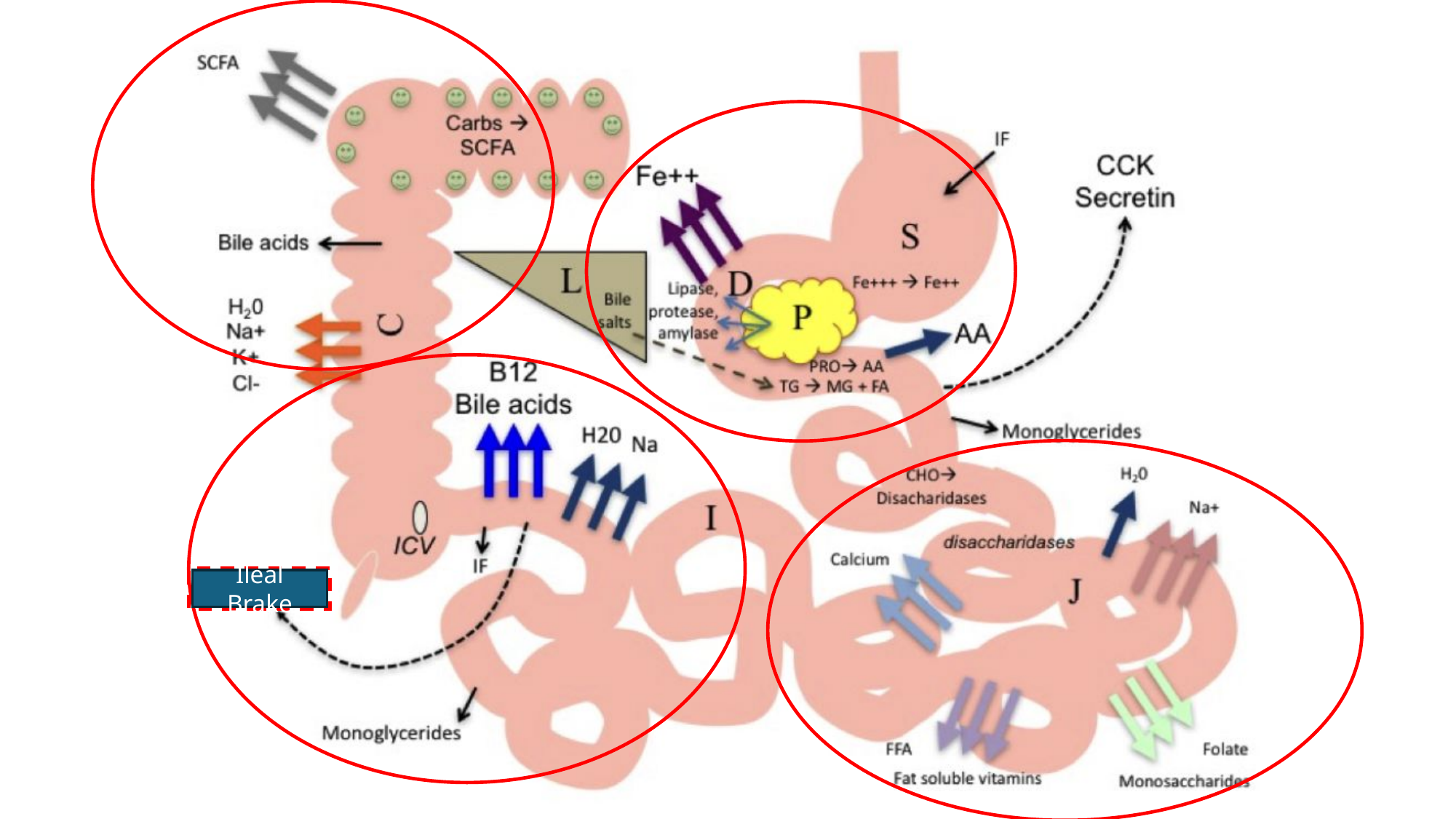

Ileal Brake

## Slide 6
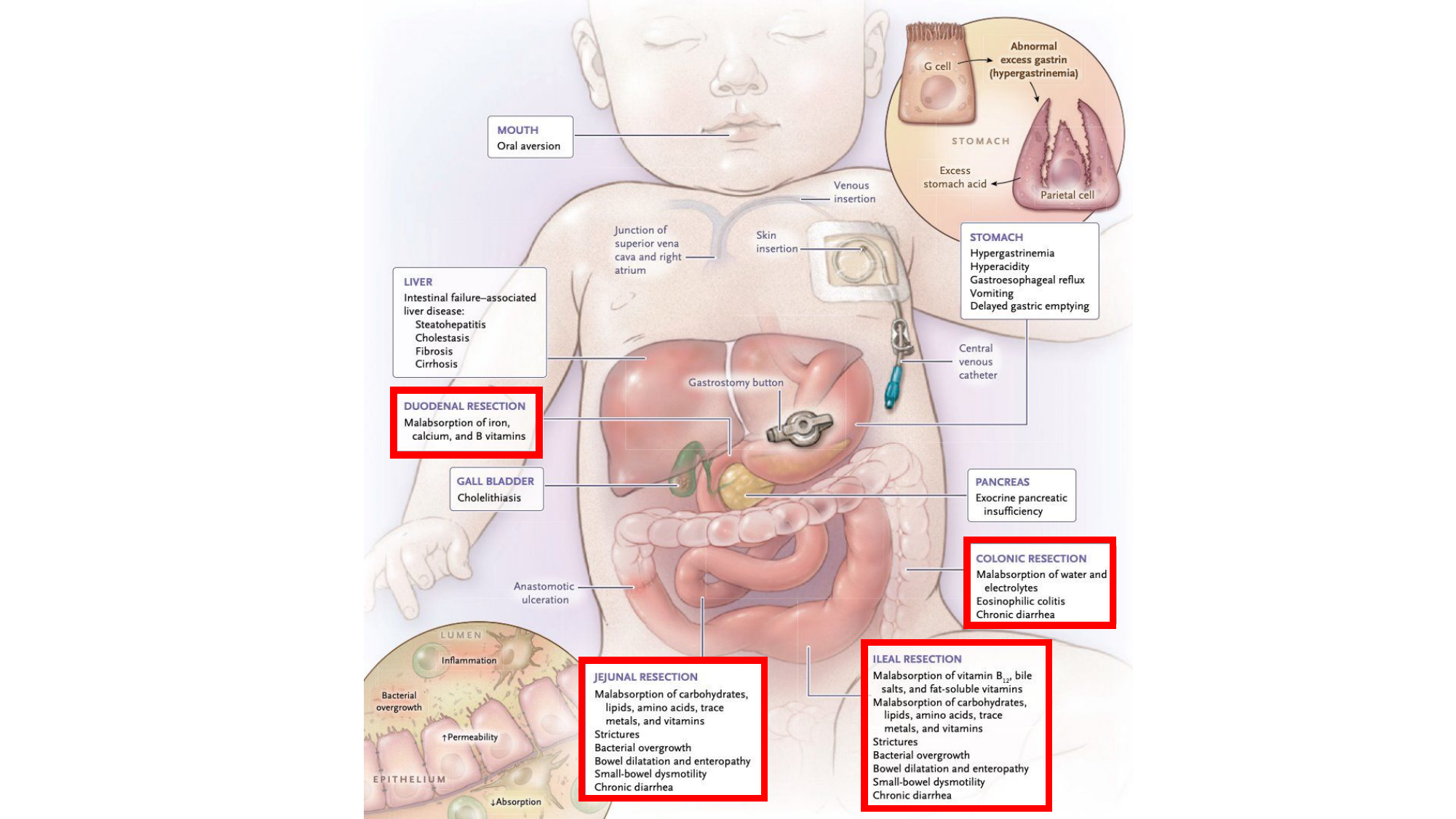

## Slide 7
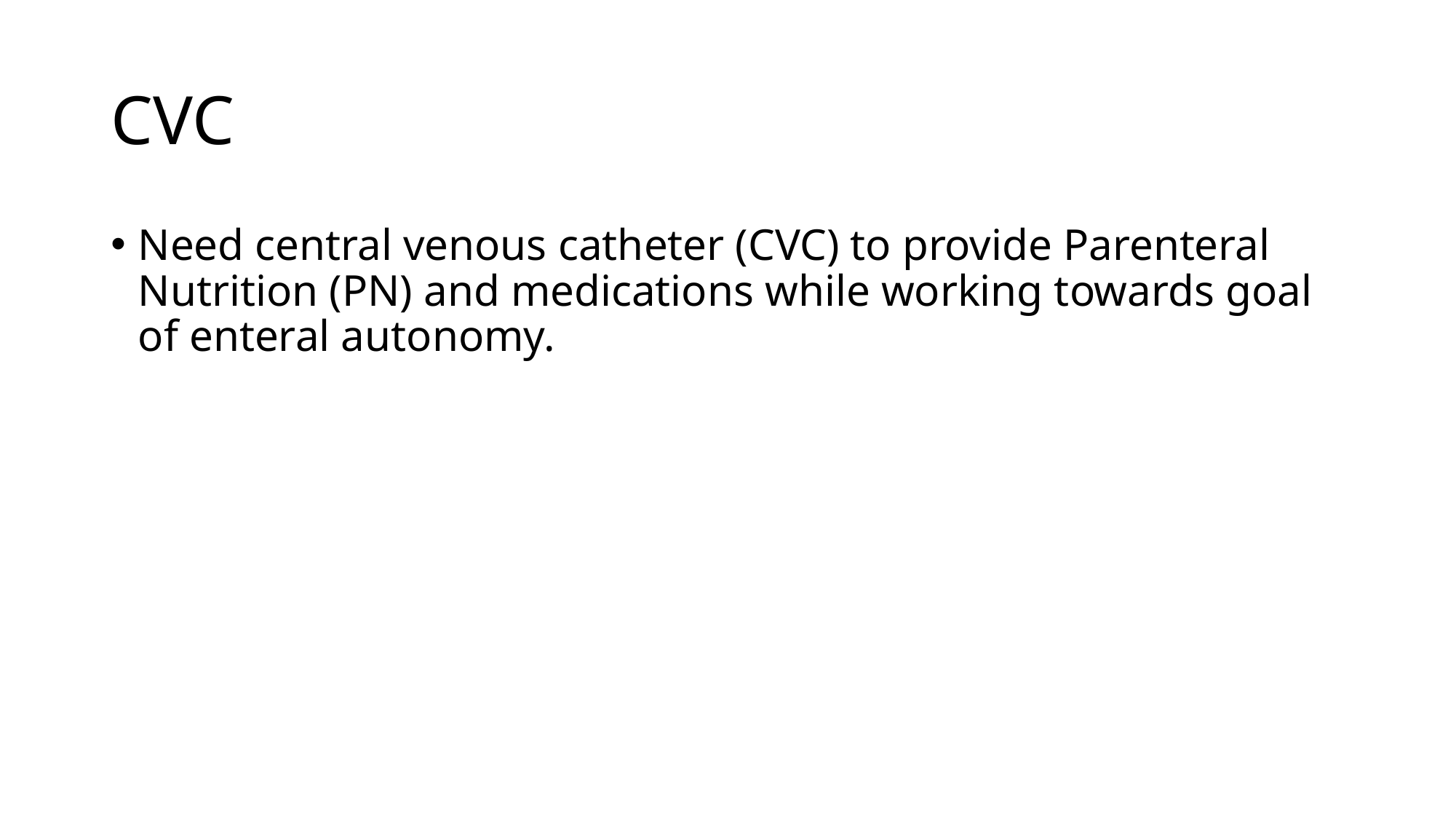

# CVC
Need central venous catheter (CVC) to provide Parenteral Nutrition (PN) and medications while working towards goal of enteral autonomy.

## Slide 8
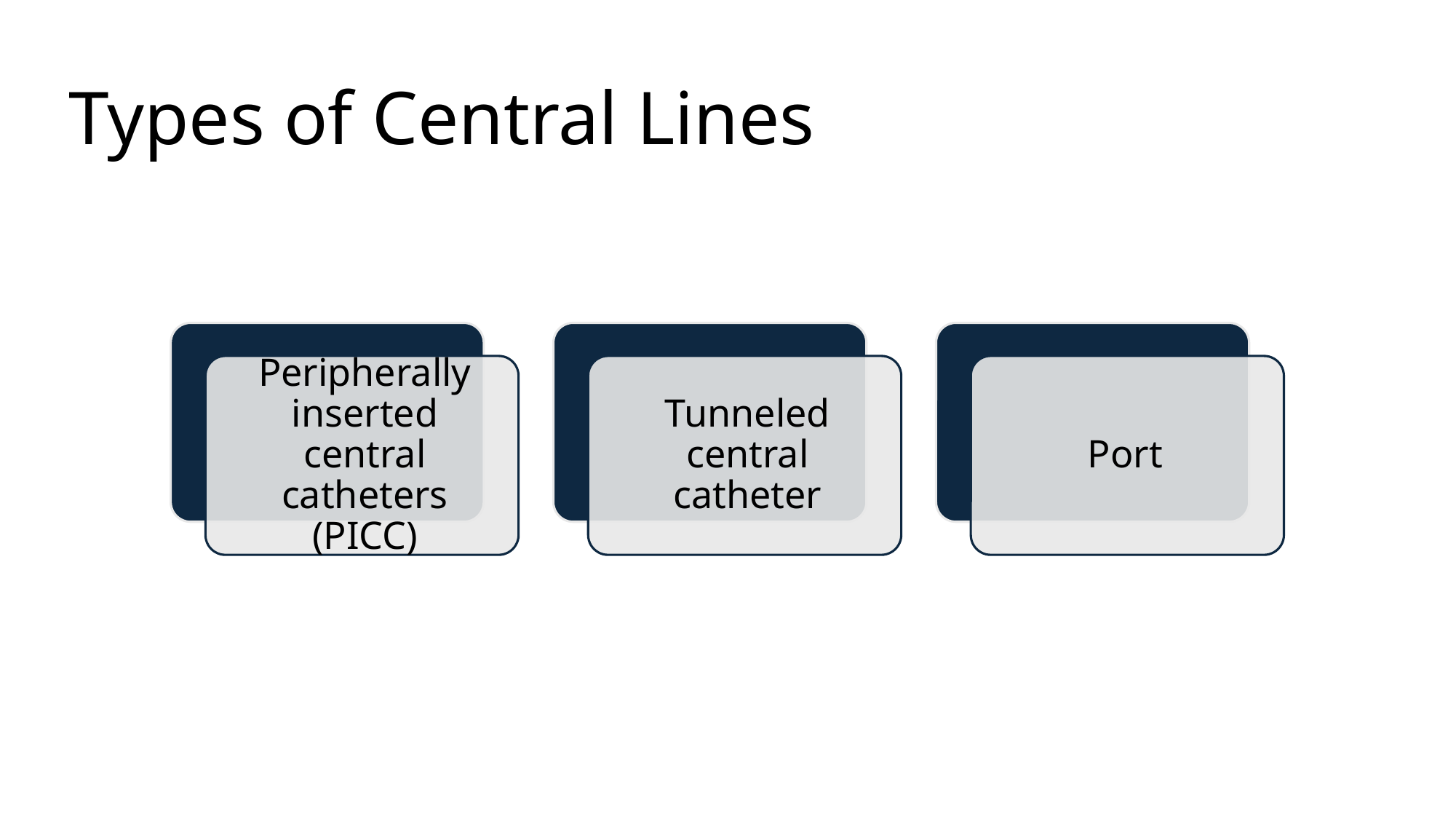

# Types of Central Lines

## Slide 9
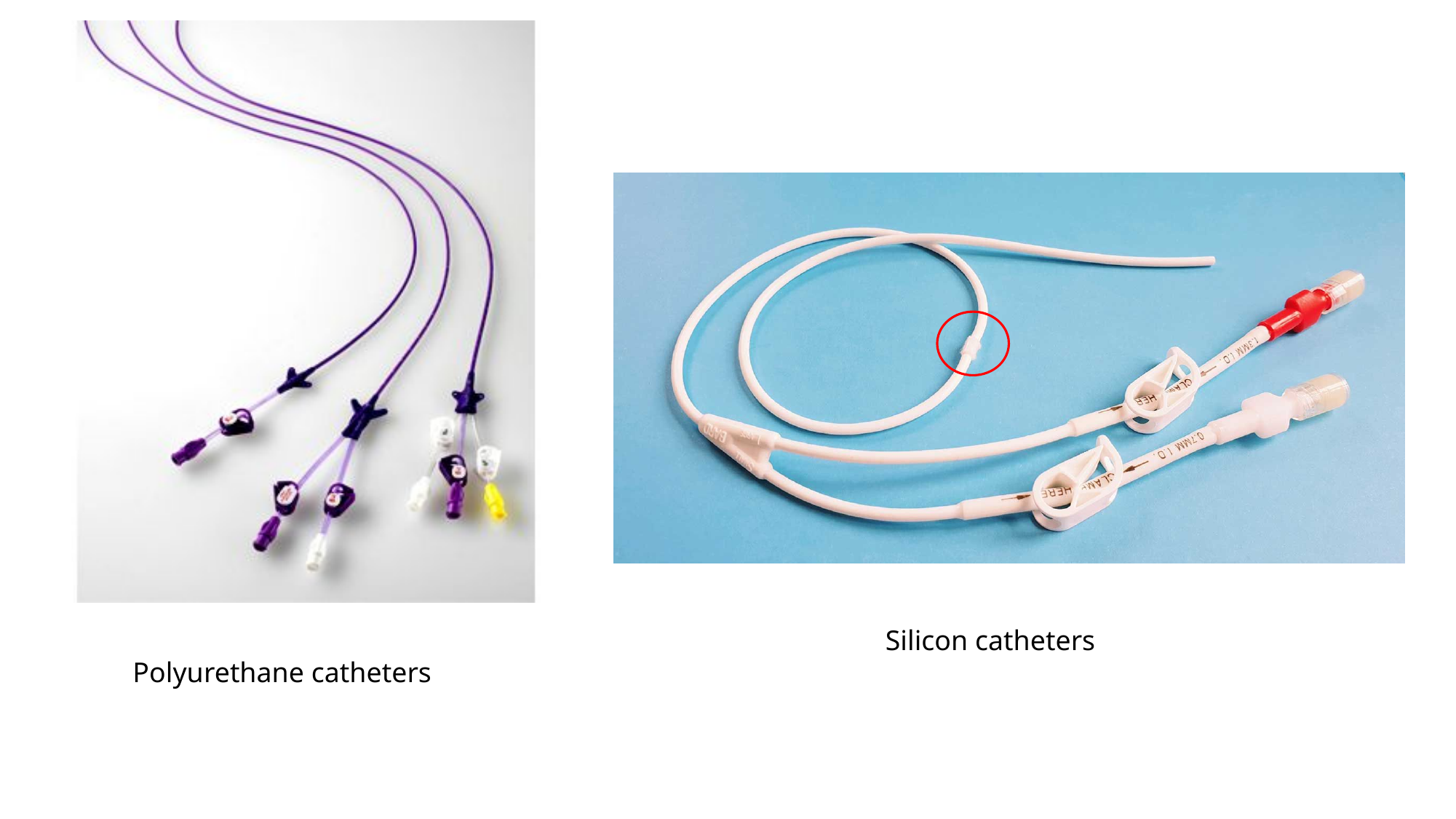

Silicon catheters
Polyurethane catheters

## Slide 10
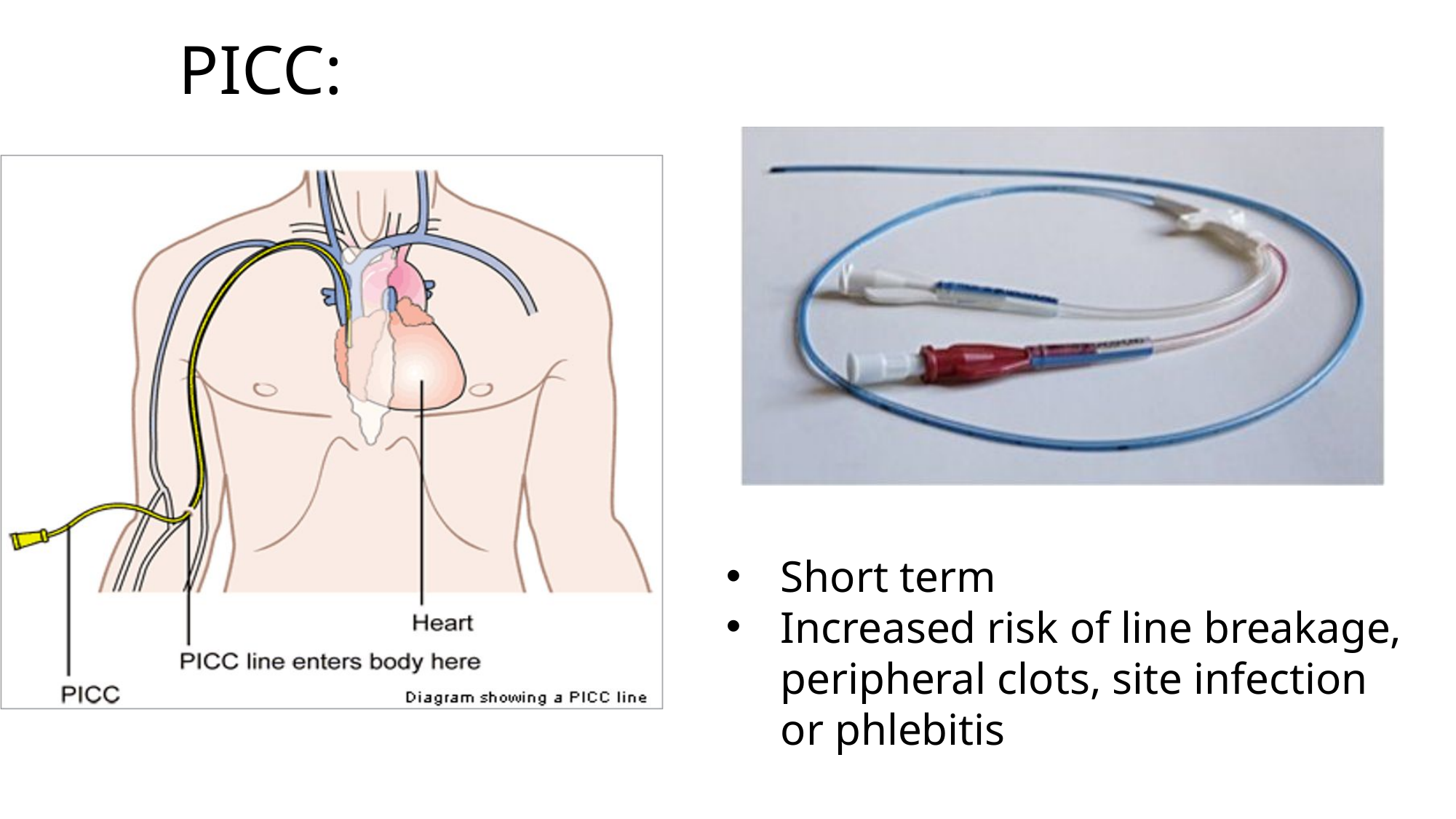

PICC:
Short term
Increased risk of line breakage, peripheral clots, site infection or phlebitis

## Slide 11
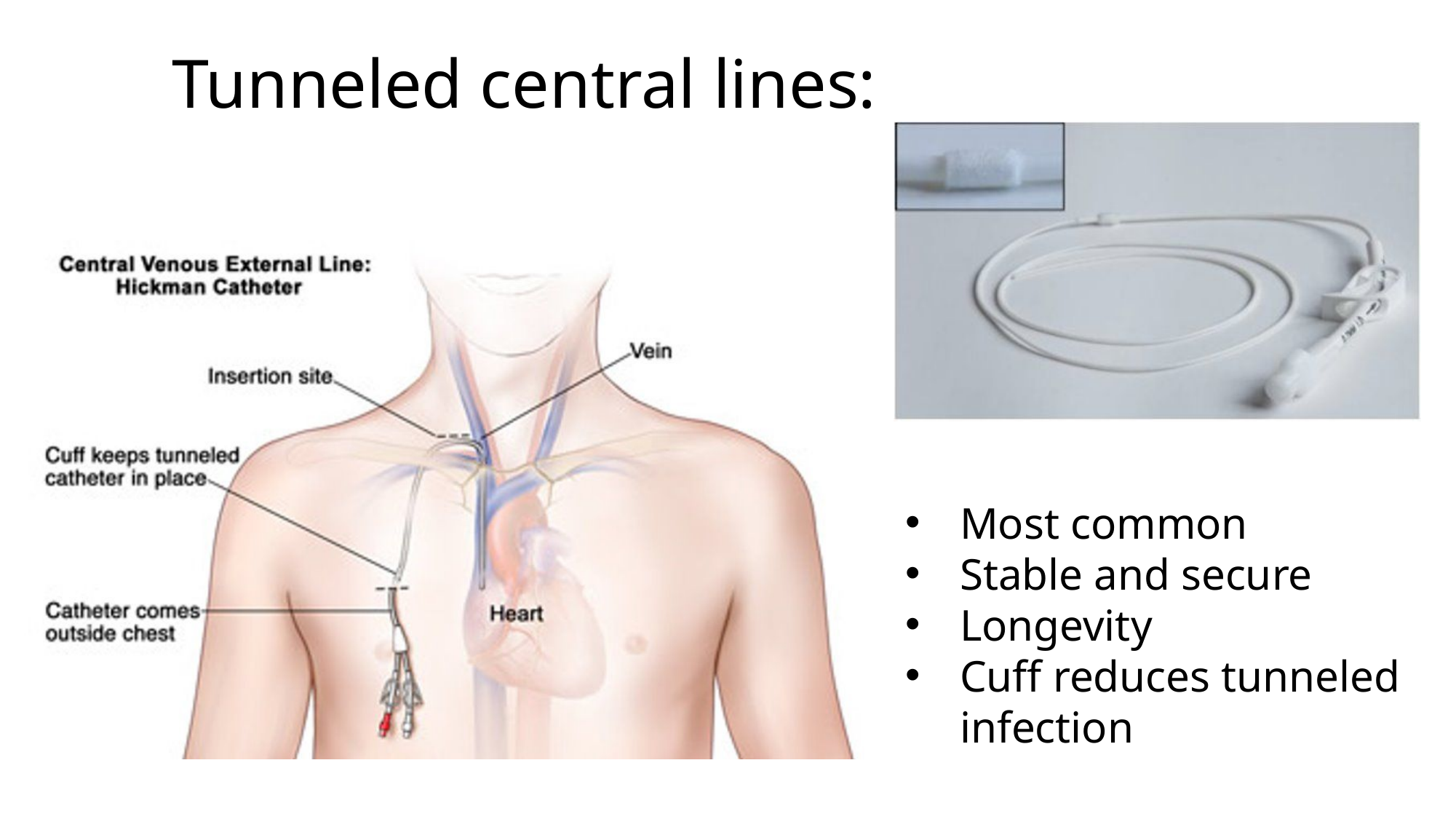

Tunneled central lines:
Most common
Stable and secure
Longevity
Cuff reduces tunneled infection

## Slide 12
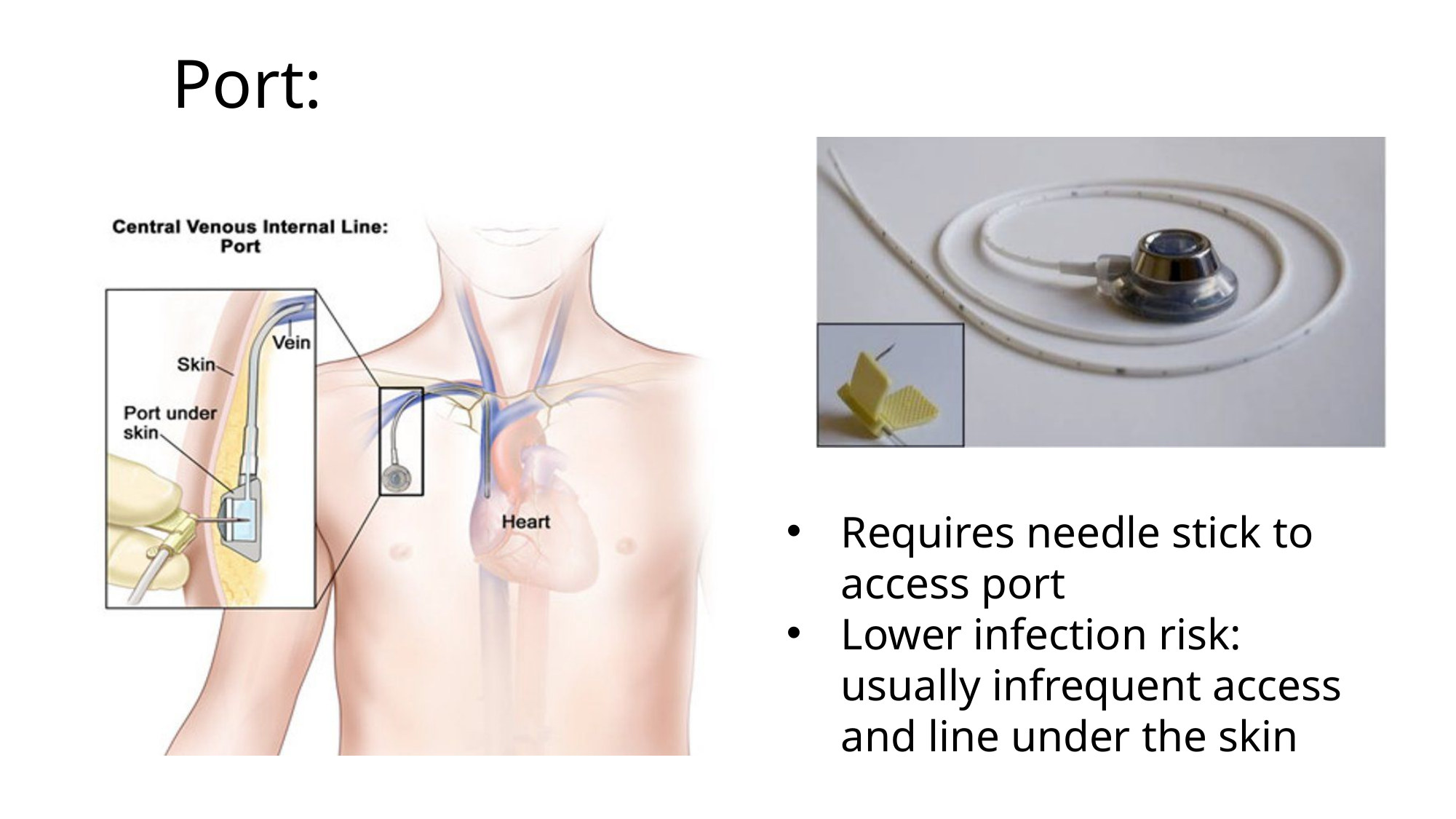

Port:
Requires needle stick to access port
Lower infection risk: usually infrequent access and line under the skin

## Slide 13
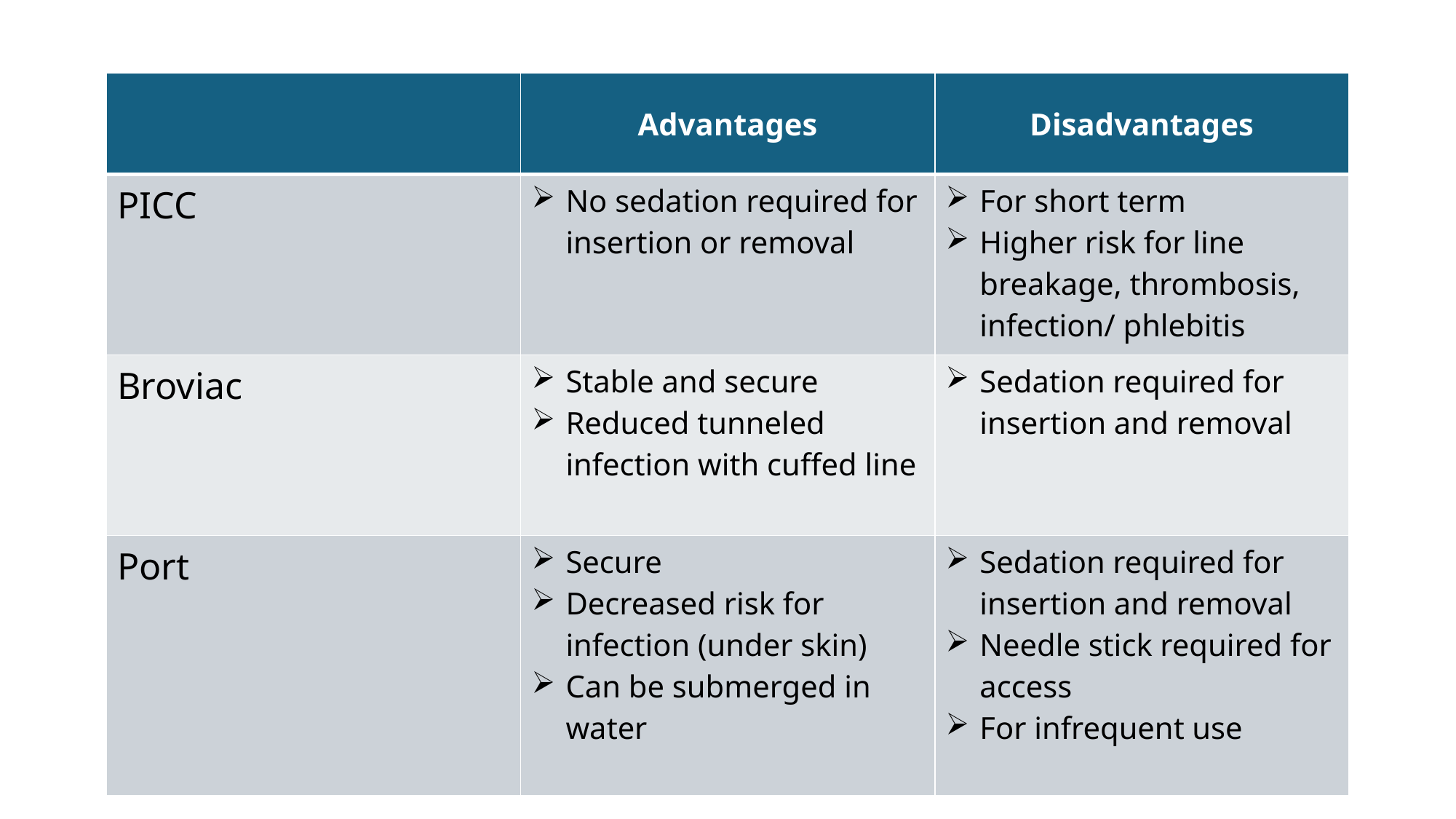

| | Advantages | Disadvantages |
| --- | --- | --- |
| PICC | No sedation required for insertion or removal | For short term Higher risk for line breakage, thrombosis, infection/ phlebitis |
| Broviac | Stable and secure Reduced tunneled infection with cuffed line | Sedation required for insertion and removal |
| Port | Secure Decreased risk for infection (under skin) Can be submerged in water | Sedation required for insertion and removal Needle stick required for access For infrequent use |

## Slide 14
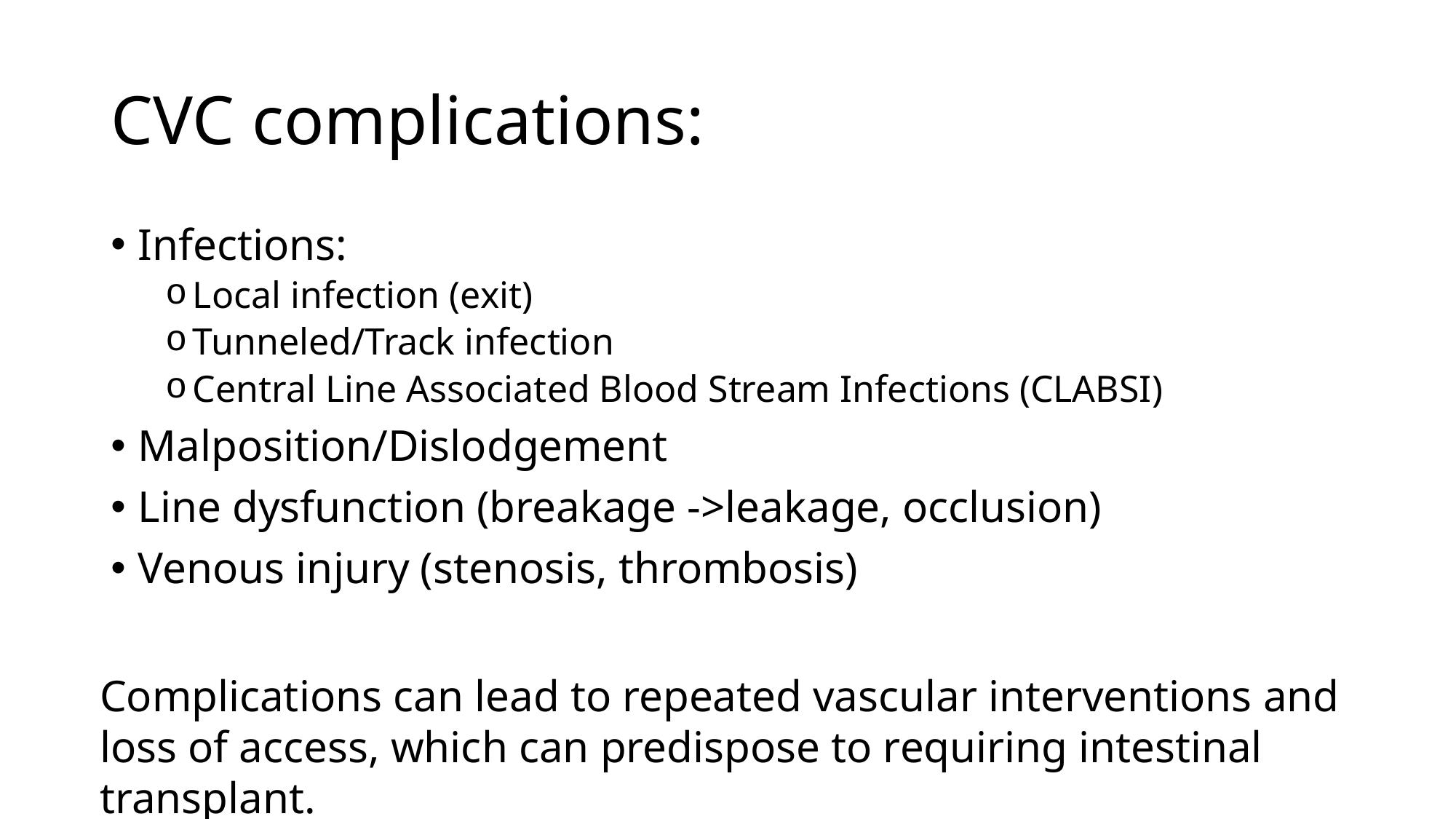

# CVC complications:
Infections:
Local infection (exit)
Tunneled/Track infection
Central Line Associated Blood Stream Infections (CLABSI)
Malposition/Dislodgement
Line dysfunction (breakage ->leakage, occlusion)
Venous injury (stenosis, thrombosis)
Complications can lead to repeated vascular interventions and loss of access, which can predispose to requiring intestinal transplant.

## Slide 15
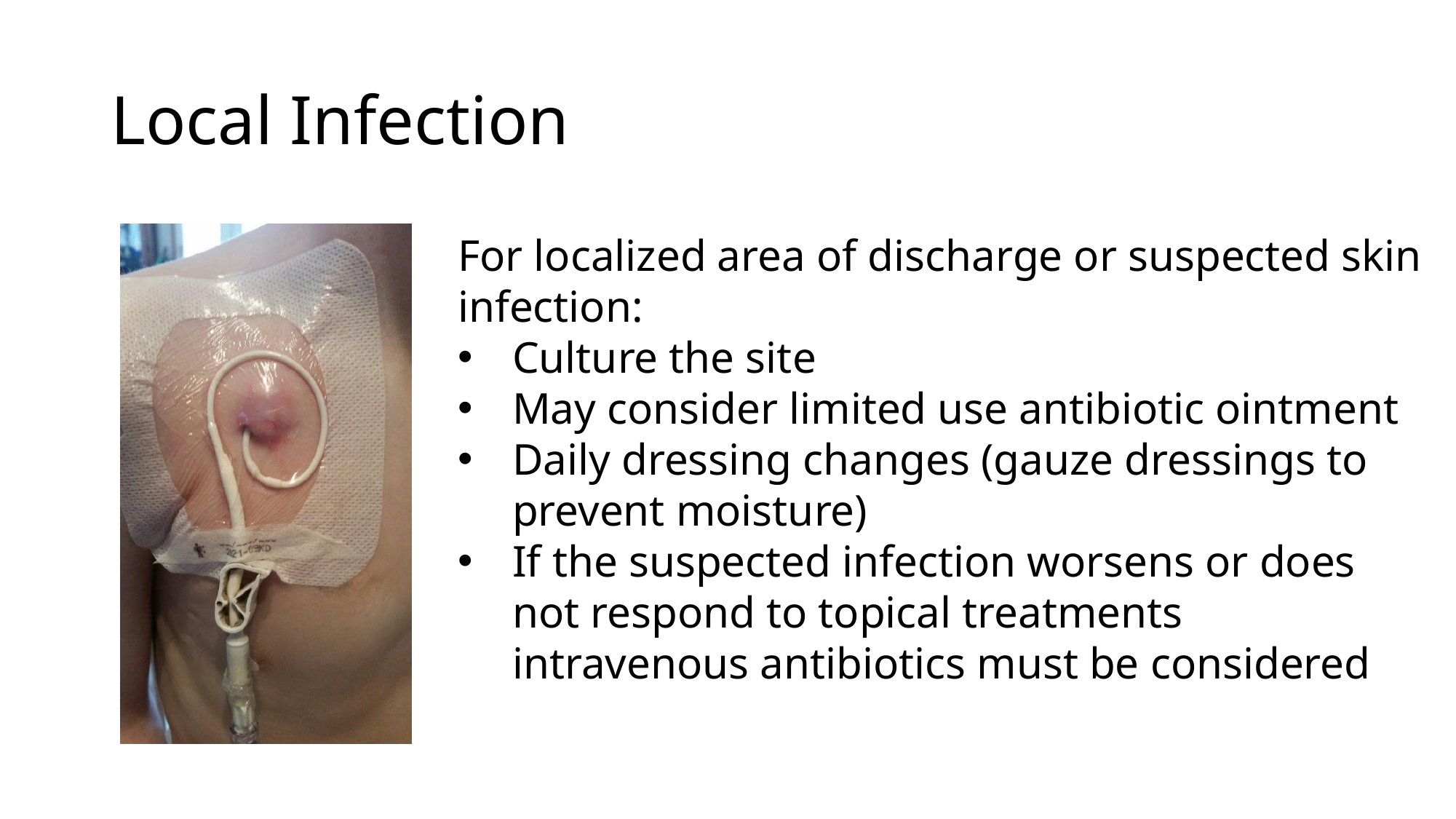

# Local Infection
For localized area of discharge or suspected skin infection:
Culture the site
May consider limited use antibiotic ointment
Daily dressing changes (gauze dressings to prevent moisture)
If the suspected infection worsens or does not respond to topical treatments intravenous antibiotics must be considered

## Slide 16
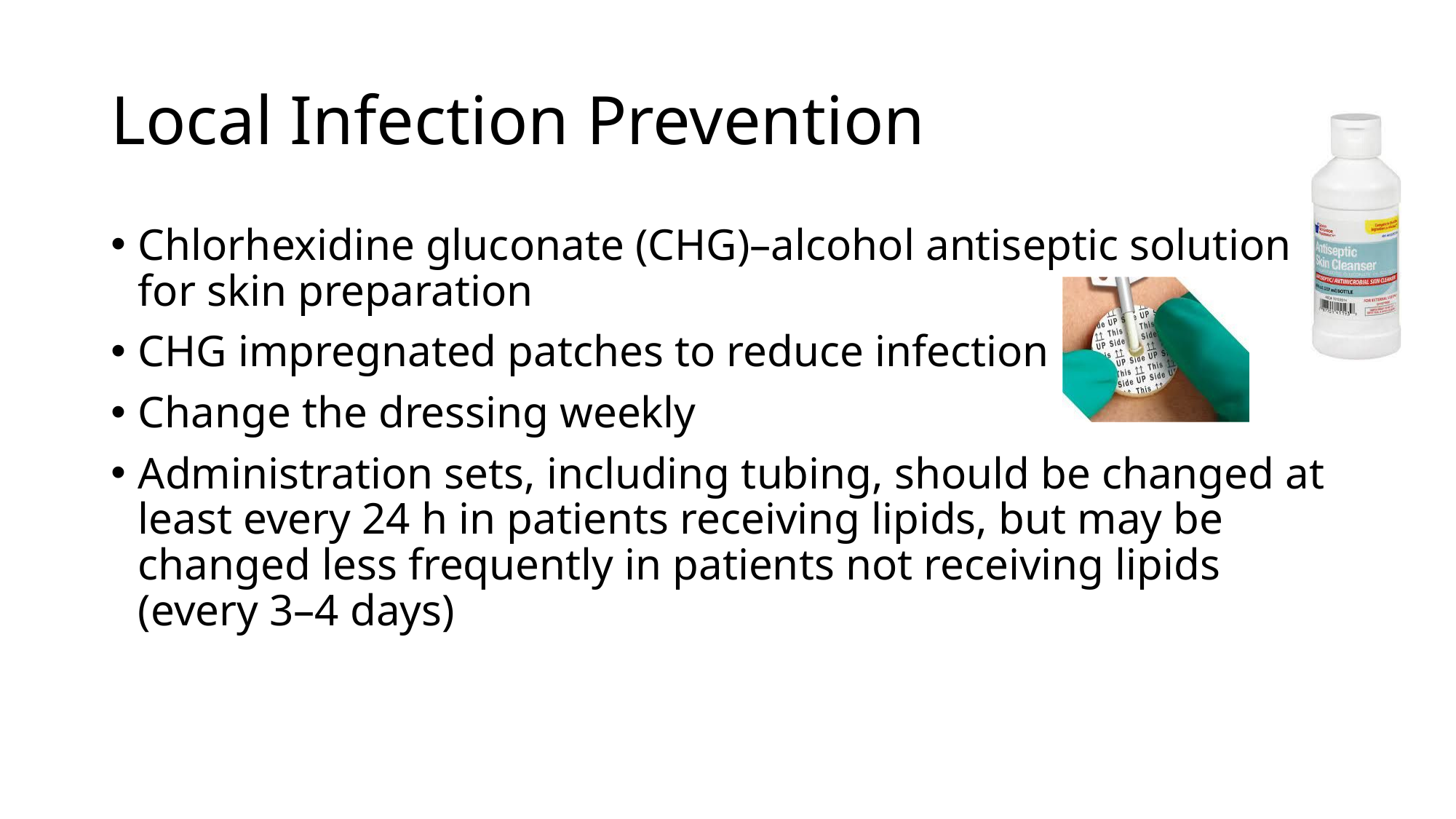

# Local Infection Prevention
Chlorhexidine gluconate (CHG)–alcohol antiseptic solution for skin preparation
CHG impregnated patches to reduce infection risk
Change the dressing weekly
Administration sets, including tubing, should be changed at least every 24 h in patients receiving lipids, but may be changed less frequently in patients not receiving lipids (every 3–4 days)

## Slide 17
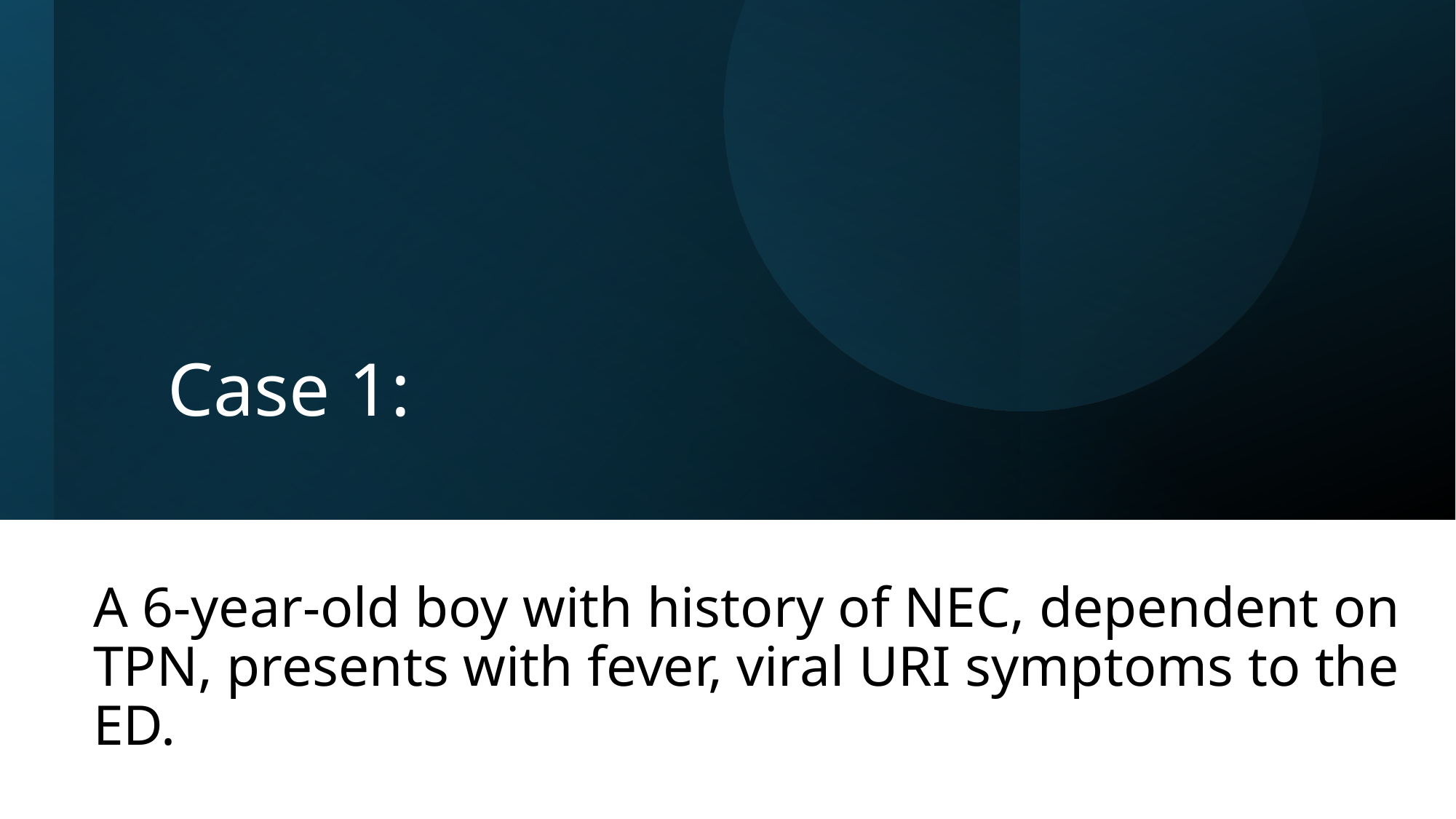

# Case 1:
A 6-year-old boy with history of NEC, dependent on TPN, presents with fever, viral URI symptoms to the ED.

## Slide 18
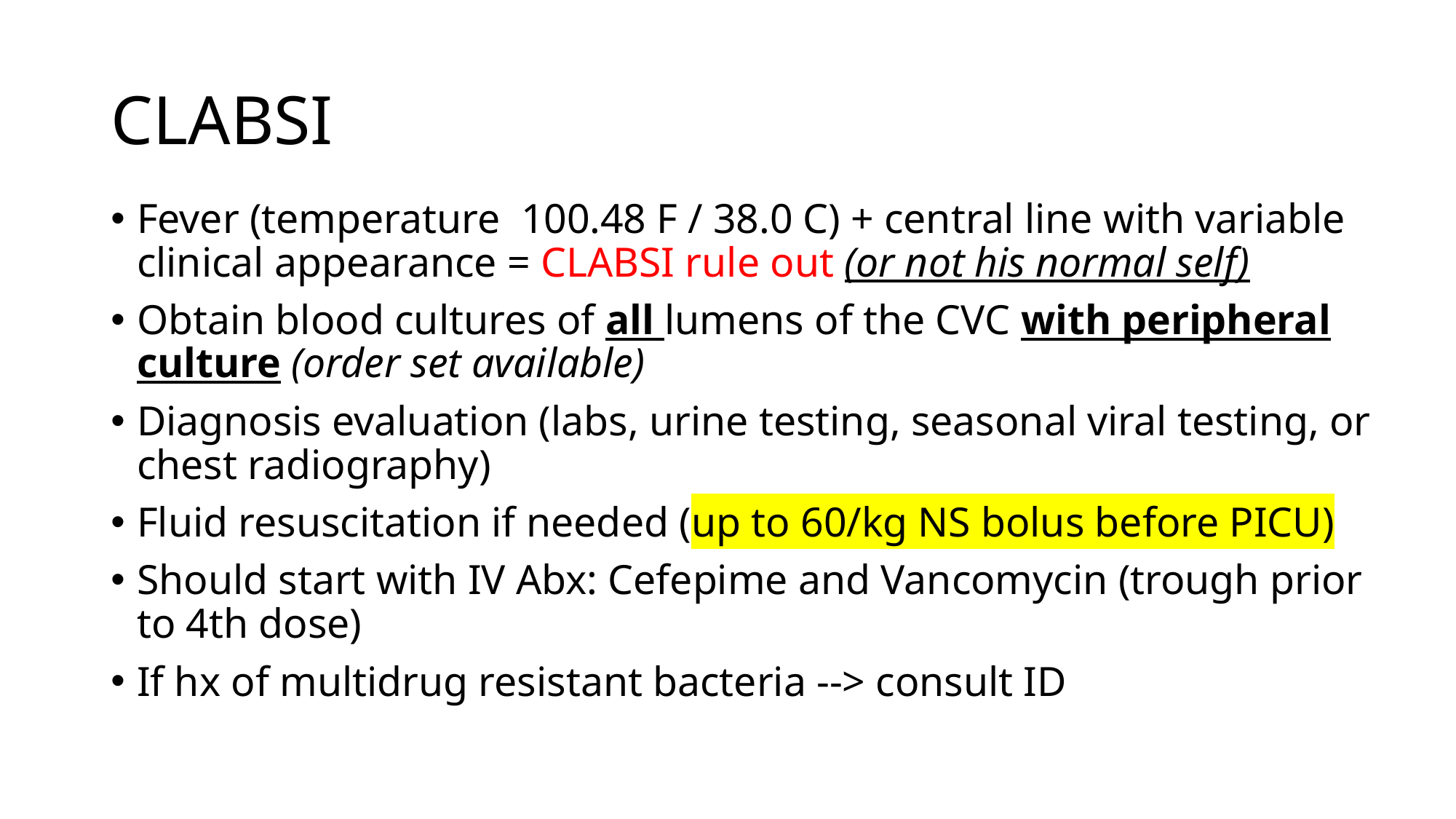

# CLABSI
Fever (temperature  100.48 F / 38.0 C) + central line with variable clinical appearance = CLABSI rule out (or not his normal self)
Obtain blood cultures of all lumens of the CVC with peripheral culture (order set available)
Diagnosis evaluation (labs, urine testing, seasonal viral testing, or chest radiography)
Fluid resuscitation if needed (up to 60/kg NS bolus before PICU)
Should start with IV Abx: Cefepime and Vancomycin (trough prior to 4th dose)
If hx of multidrug resistant bacteria --> consult ID

## Slide 19
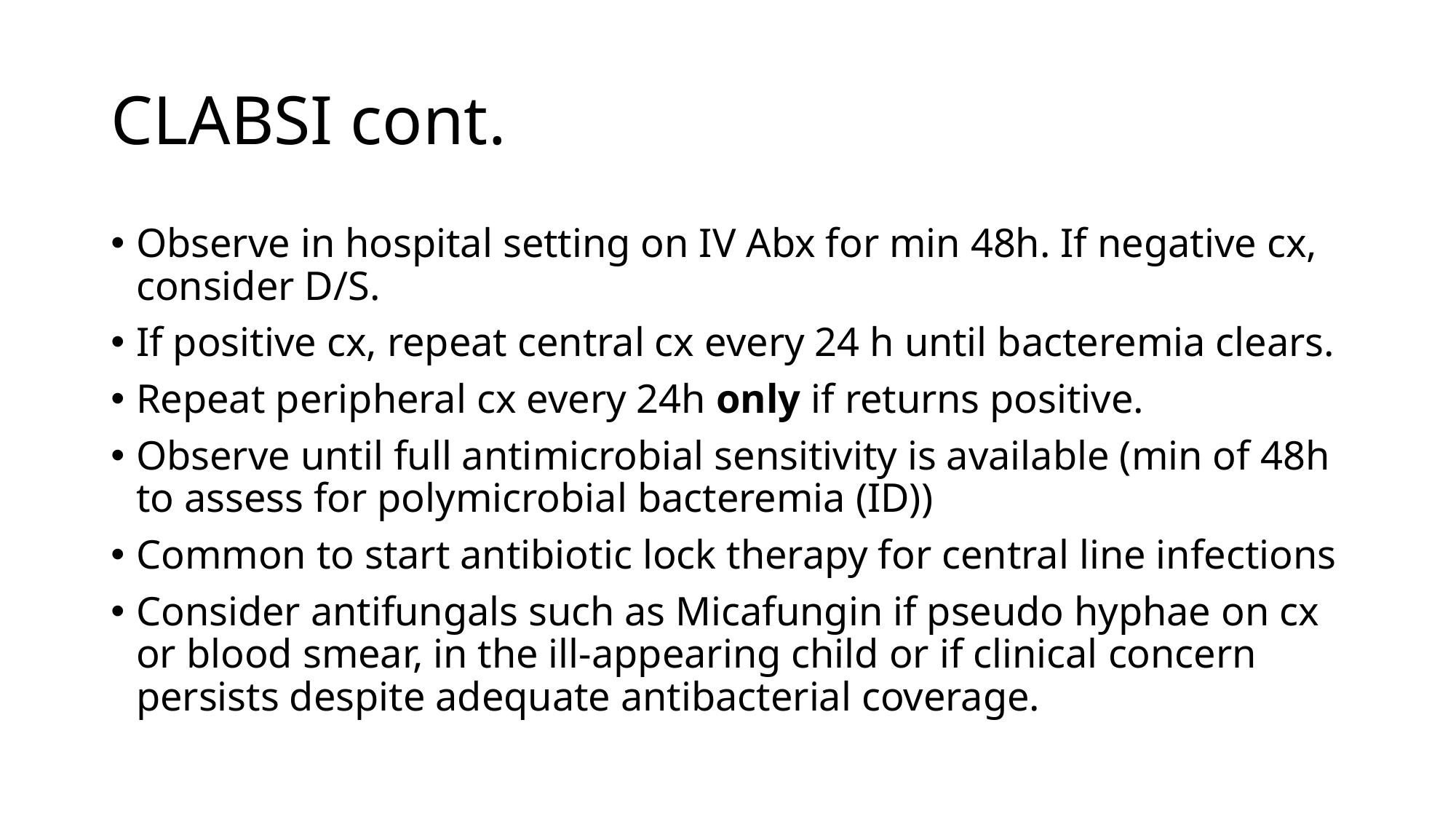

# CLABSI cont.
Observe in hospital setting on IV Abx for min 48h. If negative cx, consider D/S.
If positive cx, repeat central cx every 24 h until bacteremia clears.
Repeat peripheral cx every 24h only if returns positive.
Observe until full antimicrobial sensitivity is available (min of 48h to assess for polymicrobial bacteremia (ID))
Common to start antibiotic lock therapy for central line infections
Consider antifungals such as Micafungin if pseudo hyphae on cx or blood smear, in the ill-appearing child or if clinical concern persists despite adequate antibacterial coverage.

## Slide 20
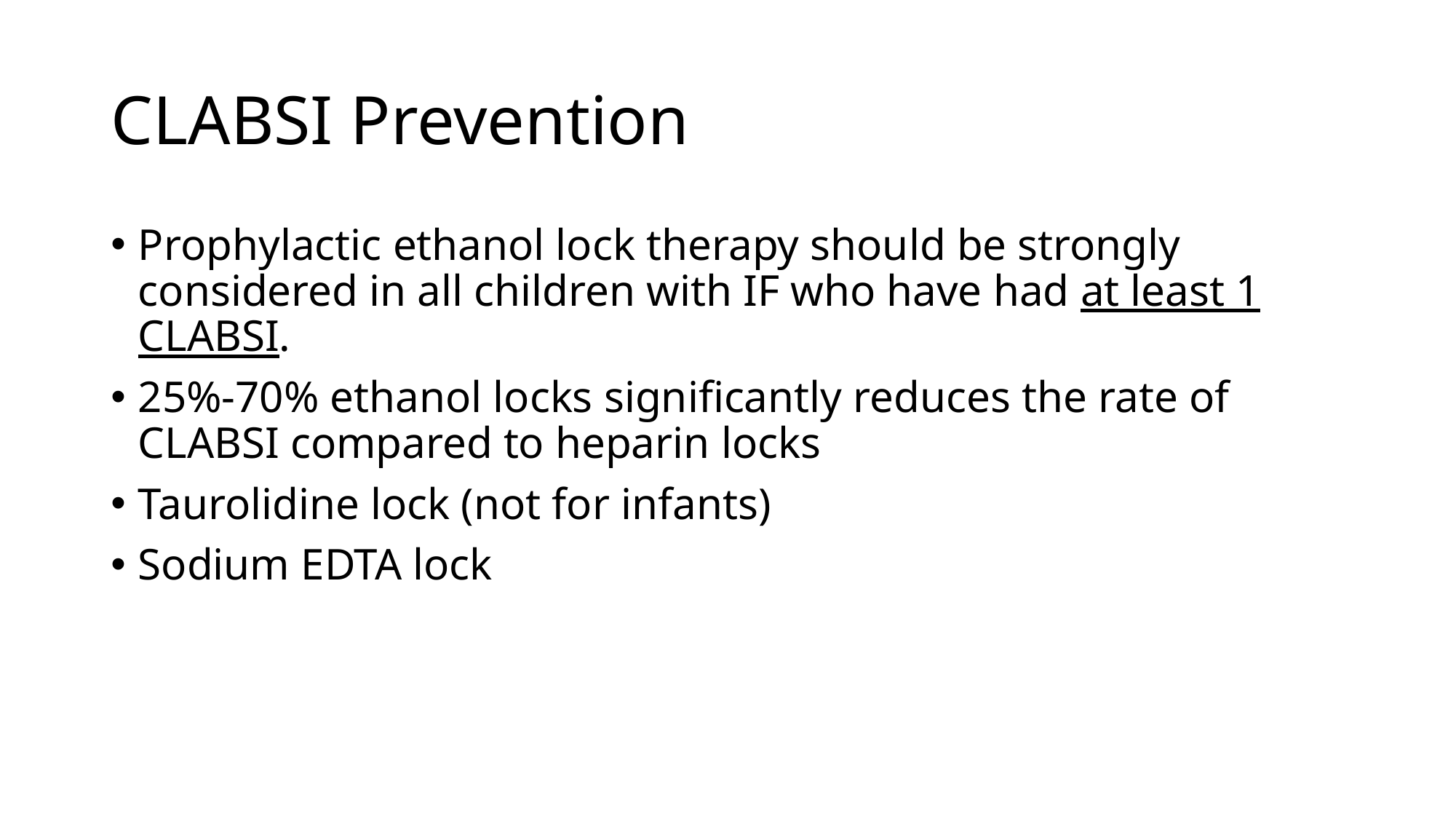

# CLABSI Prevention
Prophylactic ethanol lock therapy should be strongly considered in all children with IF who have had at least 1 CLABSI.
25%-70% ethanol locks significantly reduces the rate of CLABSI compared to heparin locks
Taurolidine lock (not for infants)
Sodium EDTA lock

## Slide 21
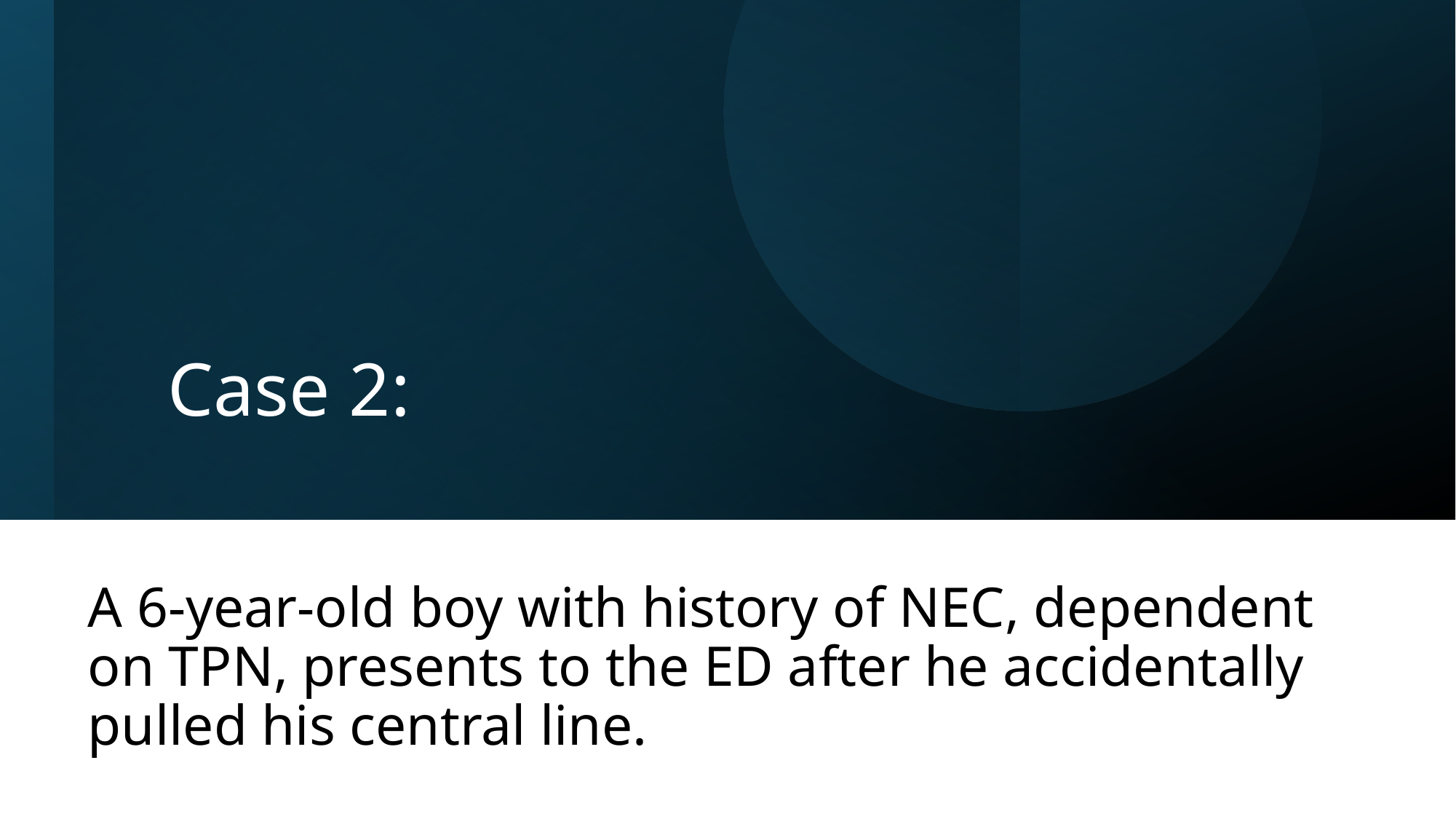

# Case 2:
A 6-year-old boy with history of NEC, dependent on TPN, presents to the ED after he accidentally pulled his central line.

## Slide 22
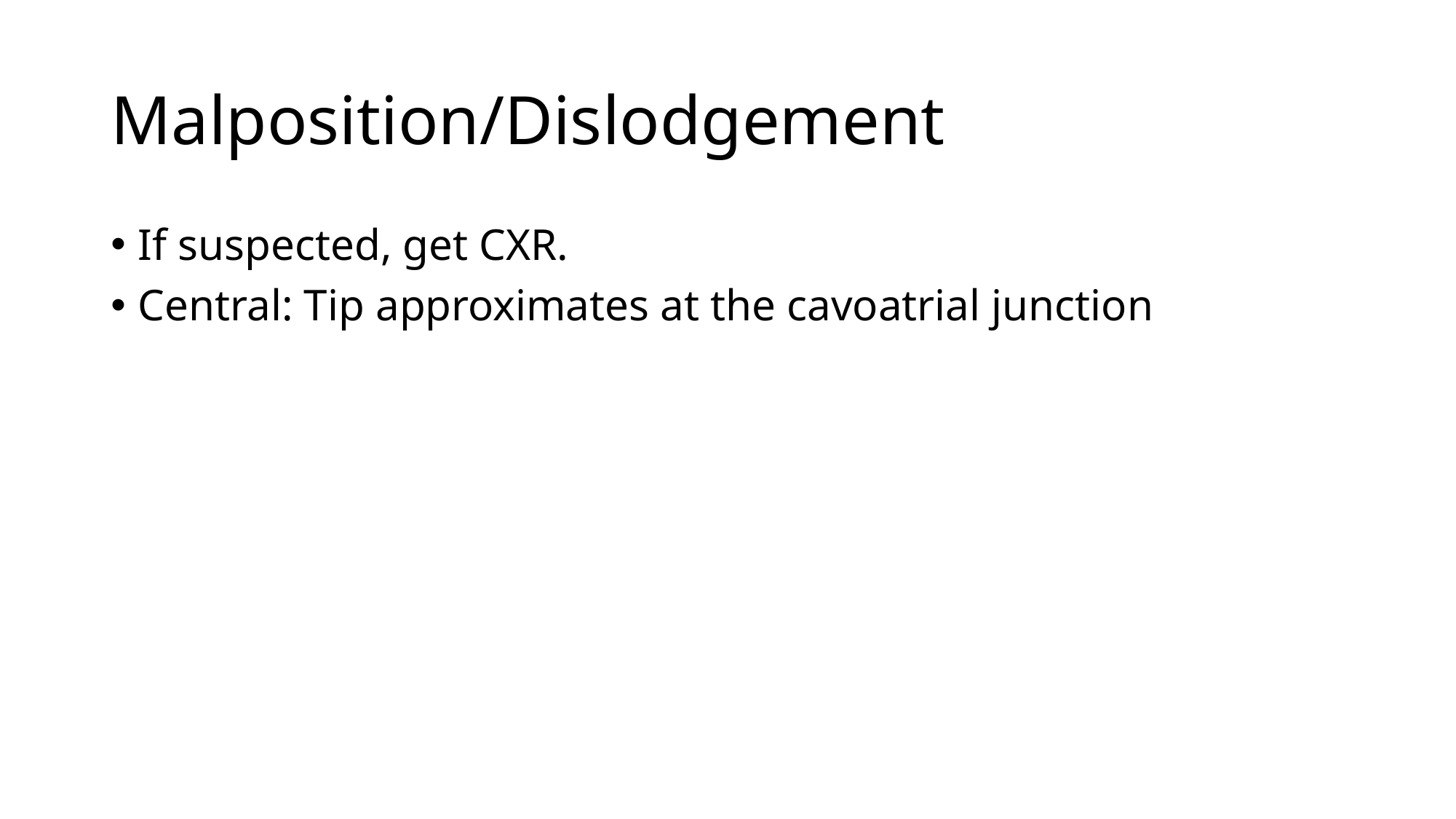

# Malposition/Dislodgement
If suspected, get CXR.
Central: Tip approximates at the cavoatrial junction

## Slide 23
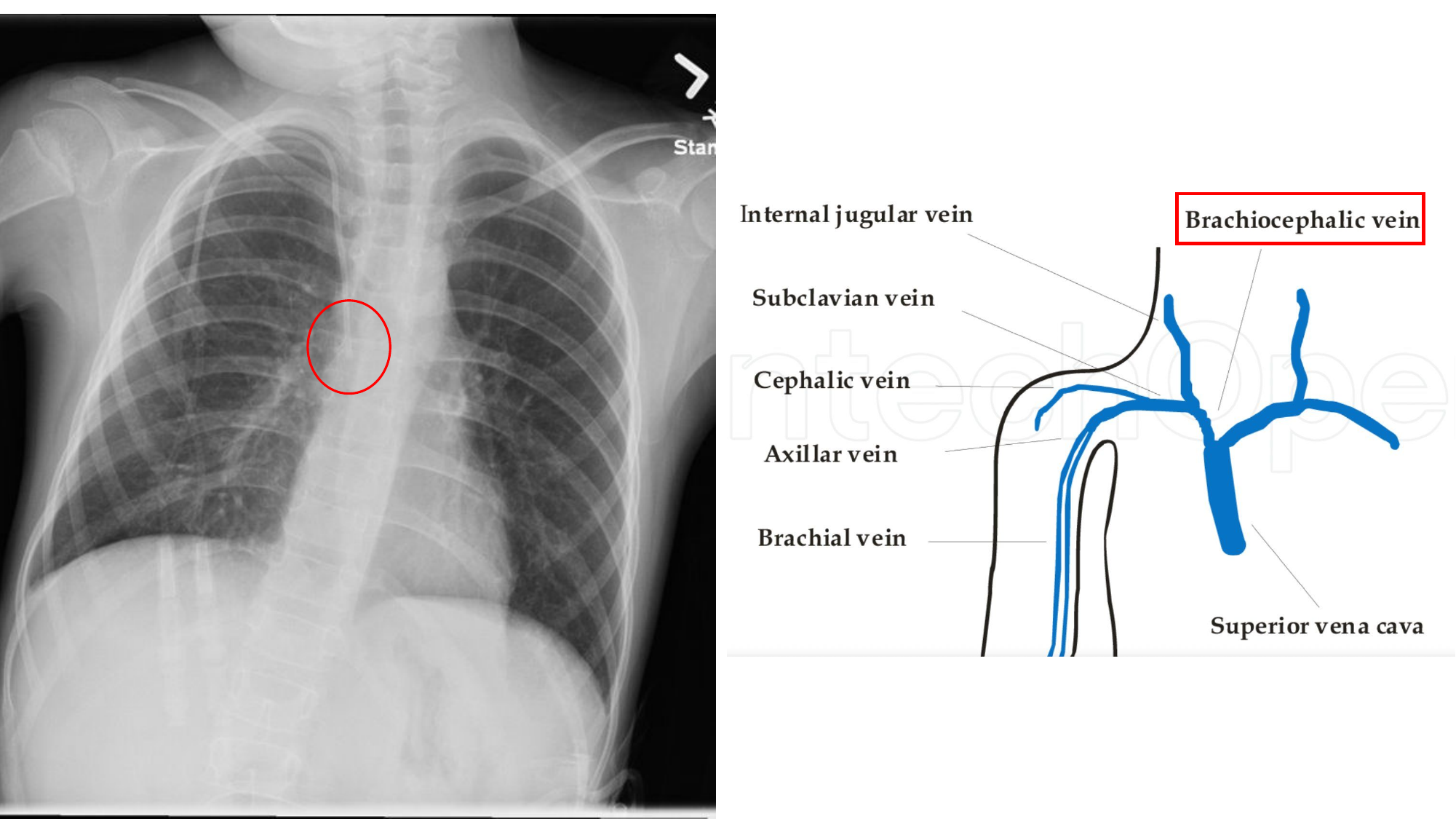

## Slide 24
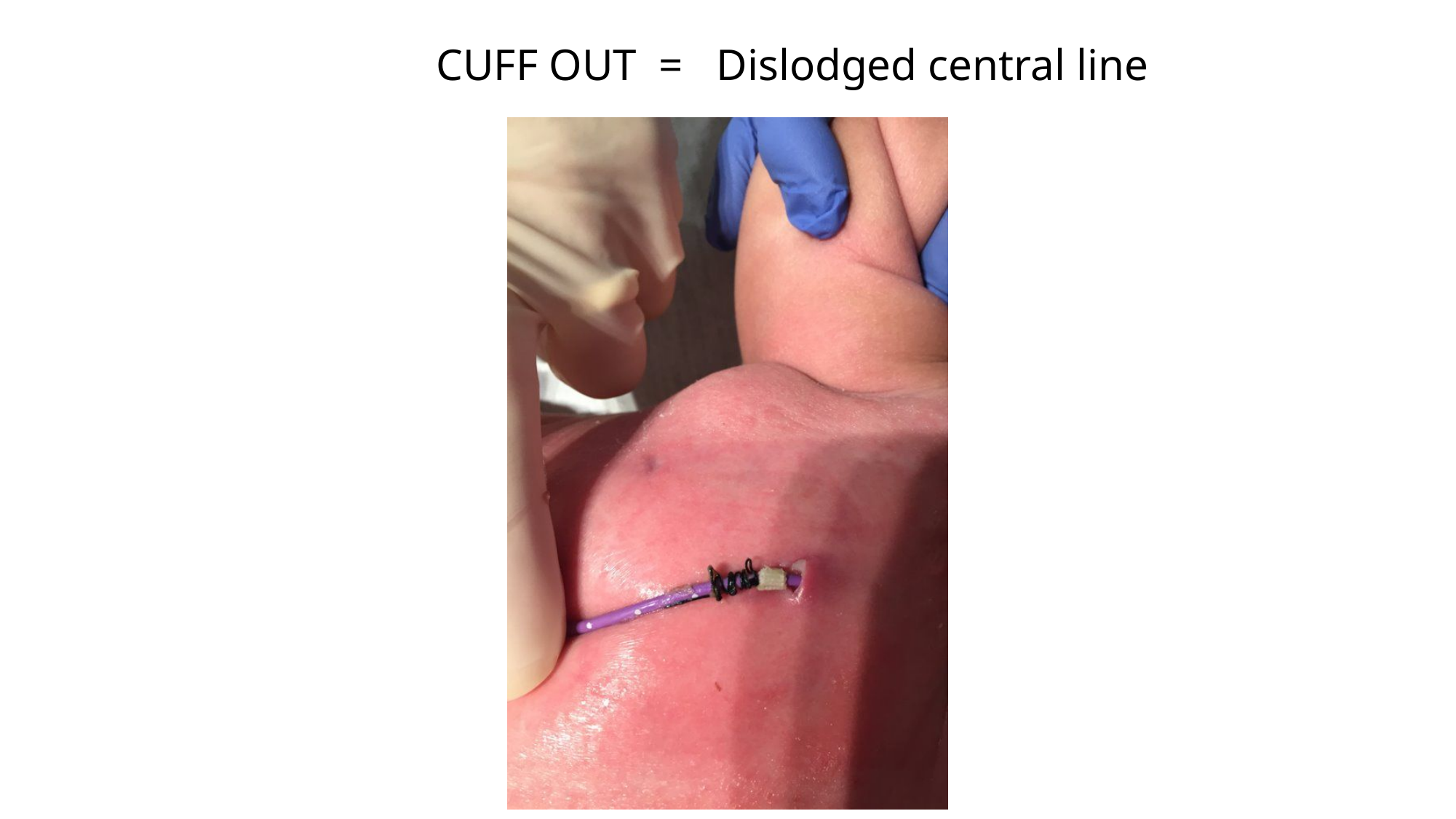

CUFF OUT =  Dislodged central line

## Slide 25
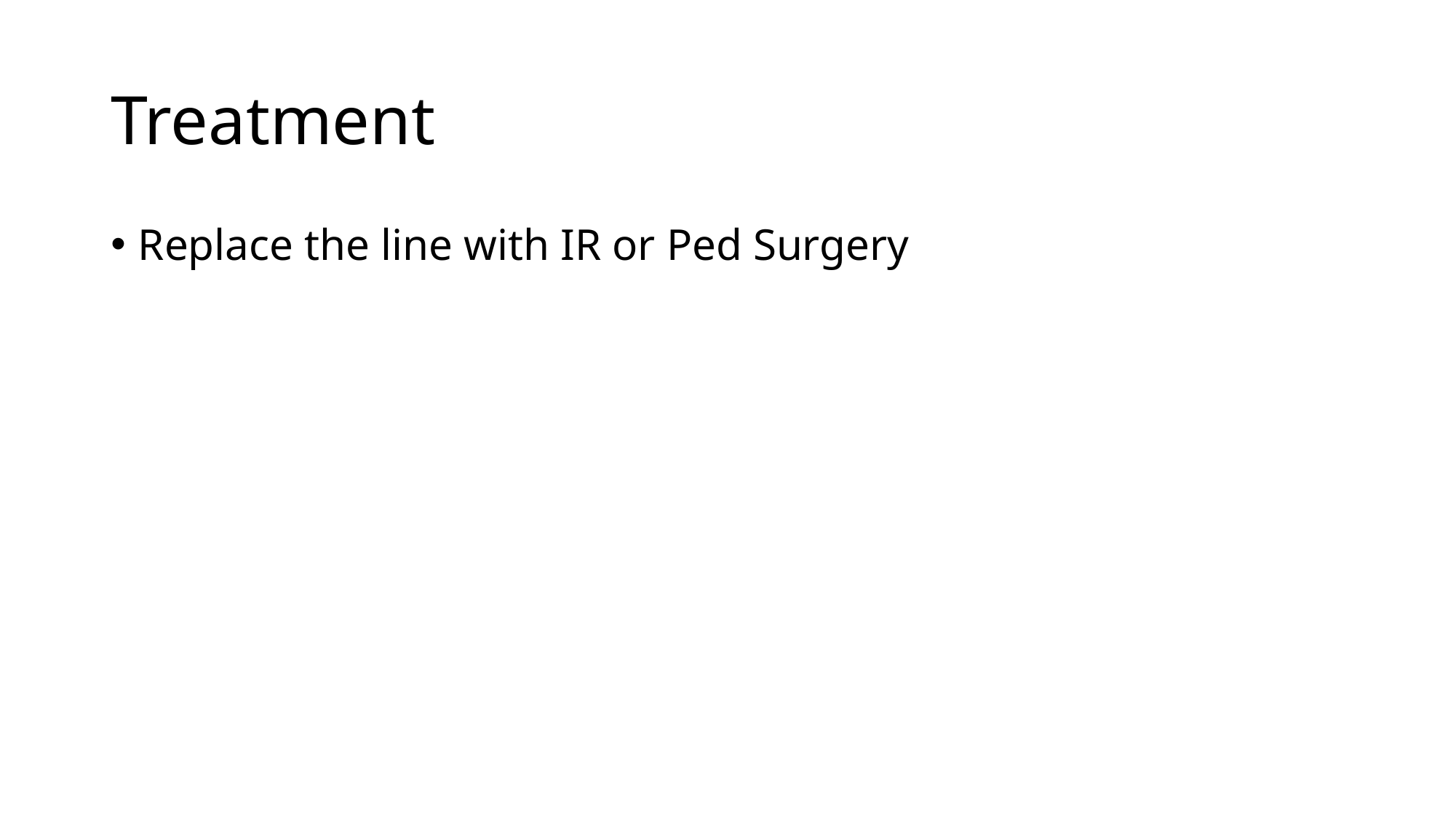

# Treatment
Replace the line with IR or Ped Surgery

## Slide 26
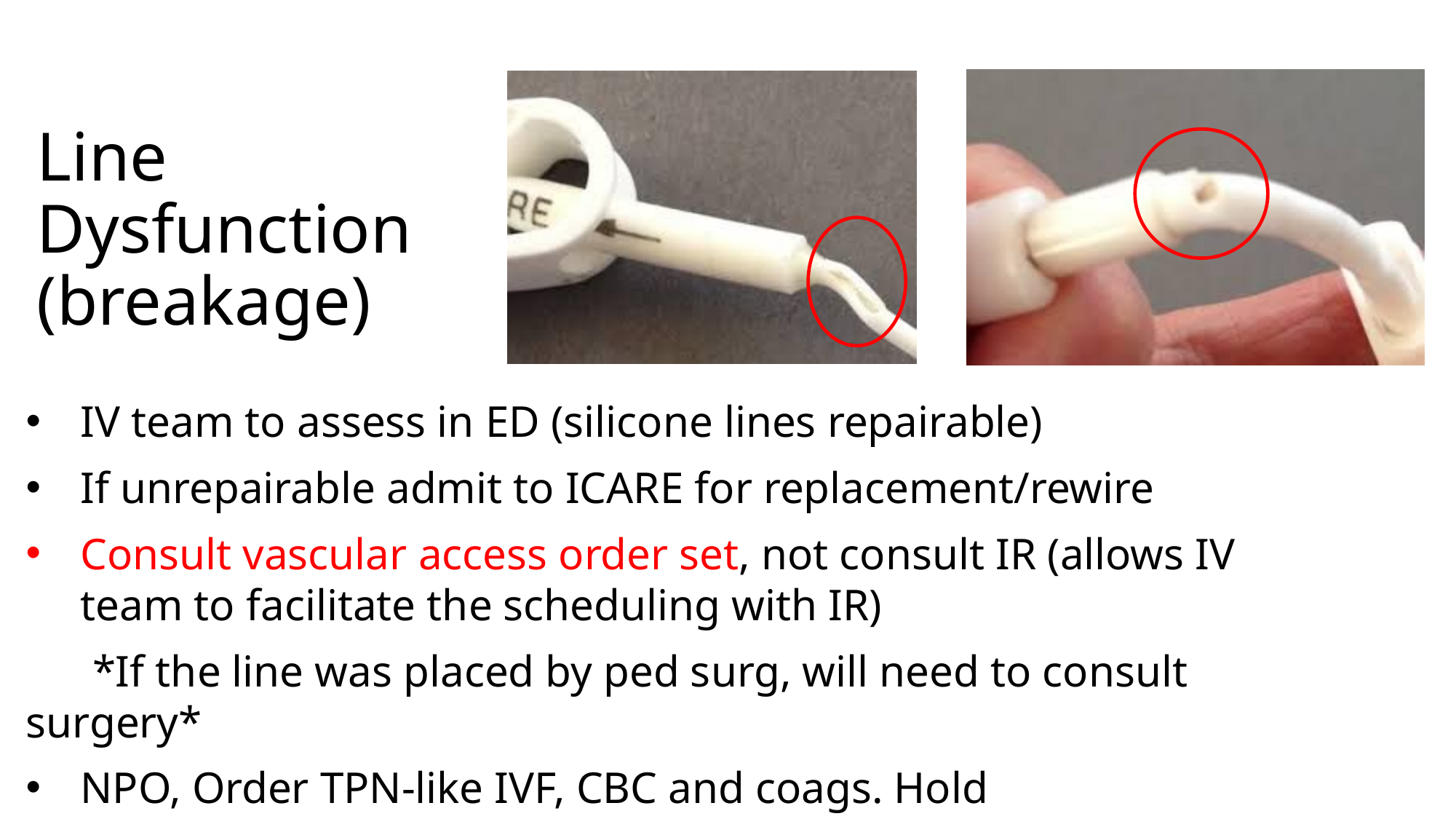

# Line Dysfunction (breakage)
IV team to assess in ED (silicone lines repairable)
If unrepairable admit to ICARE for replacement/rewire
Consult vascular access order set, not consult IR (allows IV team to facilitate the scheduling with IR)
 *If the line was placed by ped surg, will need to consult surgery*
NPO, Order TPN-like IVF, CBC and coags. Hold Lovenox/Xarelto

## Slide 27
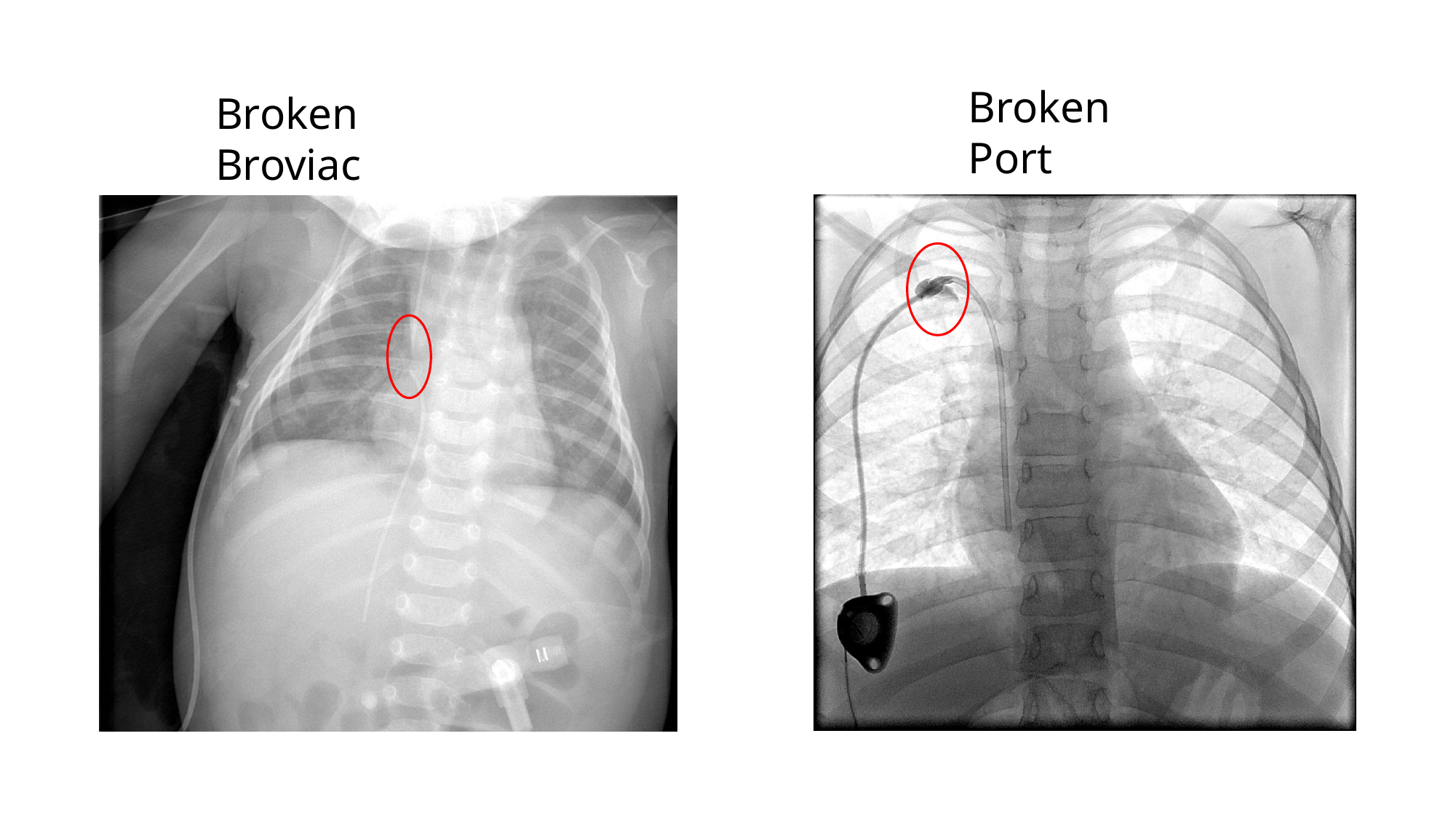

Broken Port
Broken Broviac

## Slide 28
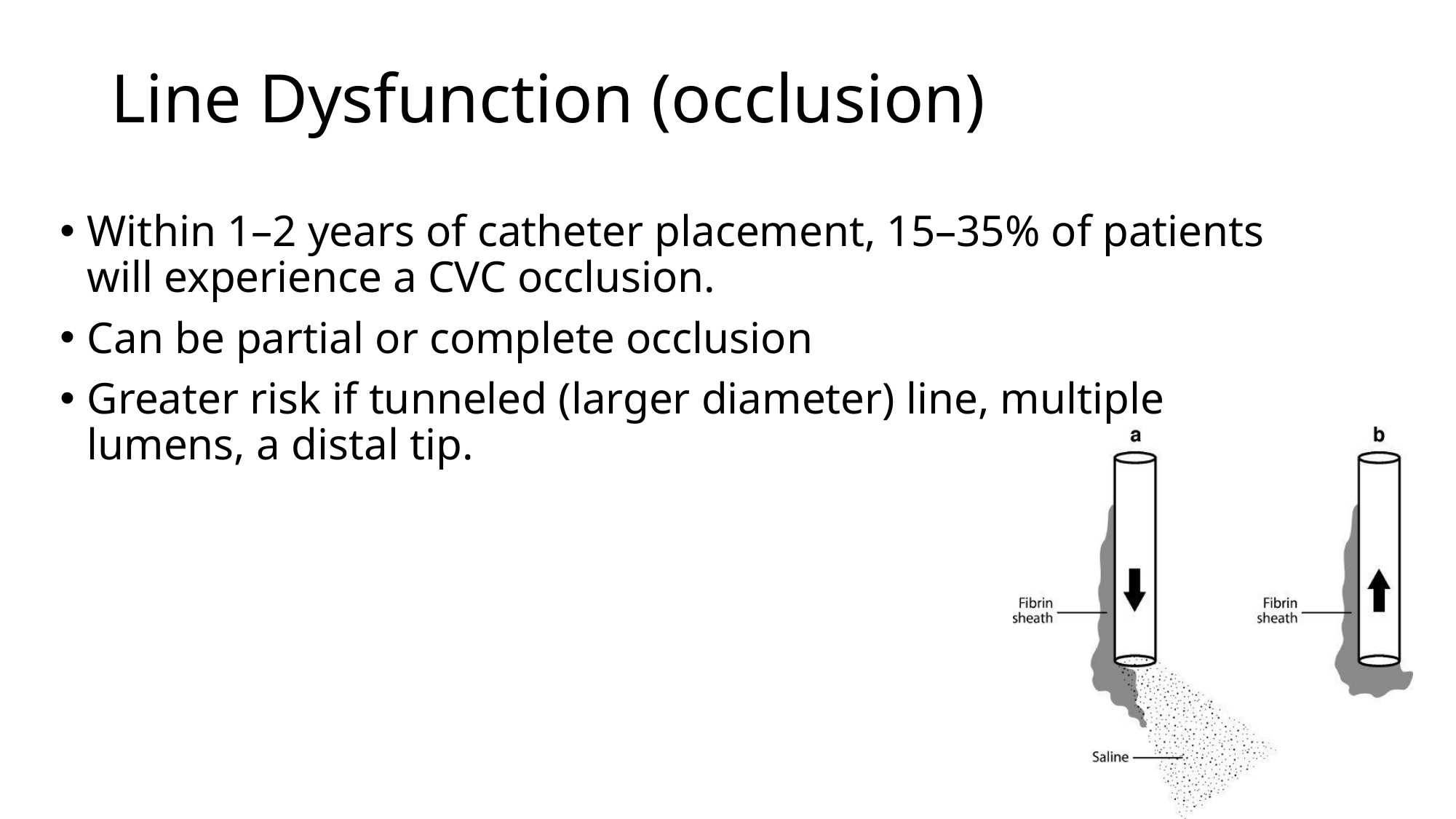

# Line Dysfunction (occlusion)
Within 1–2 years of catheter placement, 15–35% of patients will experience a CVC occlusion.
Can be partial or complete occlusion
Greater risk if tunneled (larger diameter) line, multiple lumens, a distal tip.

## Slide 29
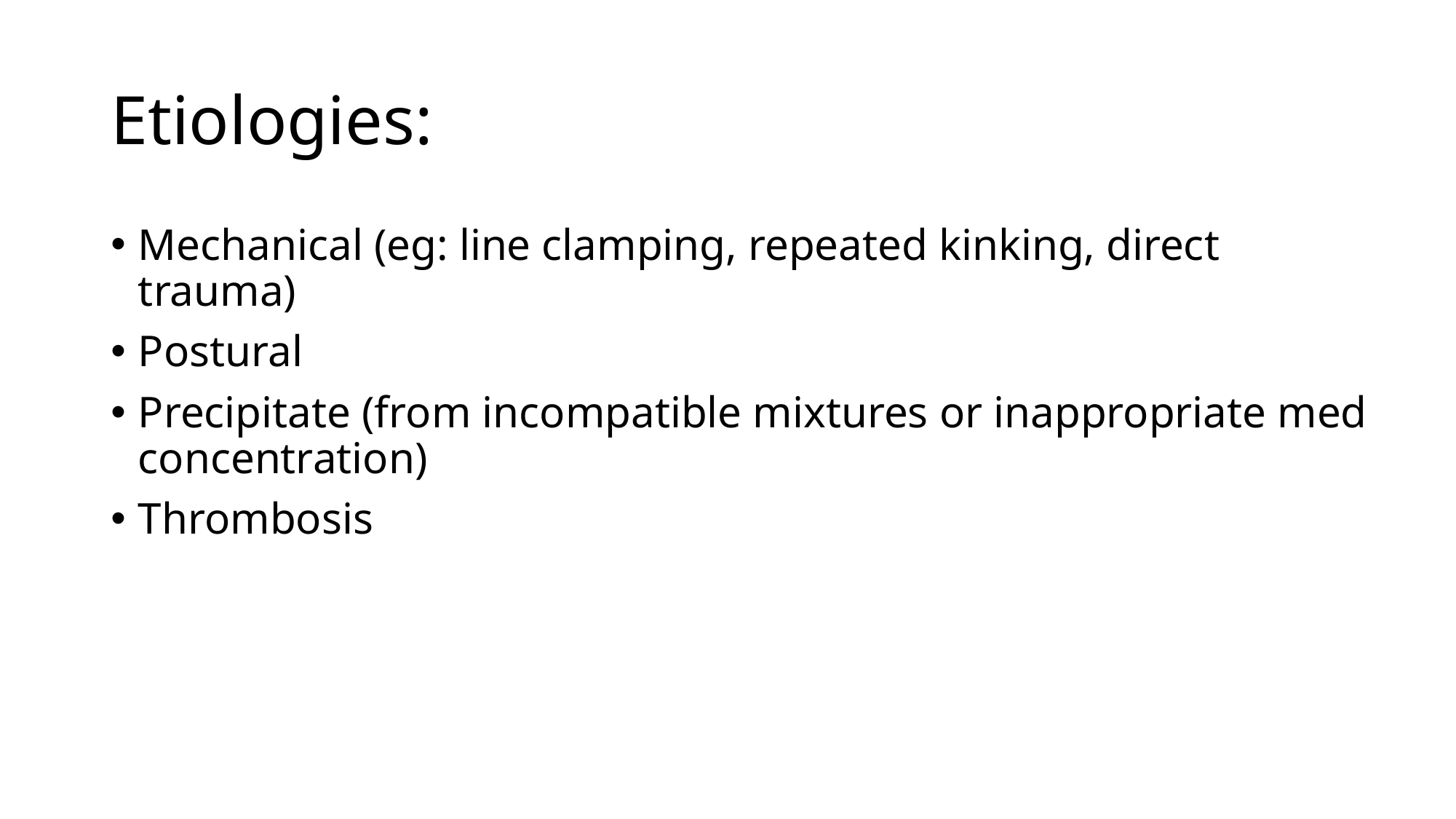

# Etiologies:
Mechanical (eg: line clamping, repeated kinking, direct trauma)
Postural
Precipitate (from incompatible mixtures or inappropriate med concentration)
Thrombosis

## Slide 30
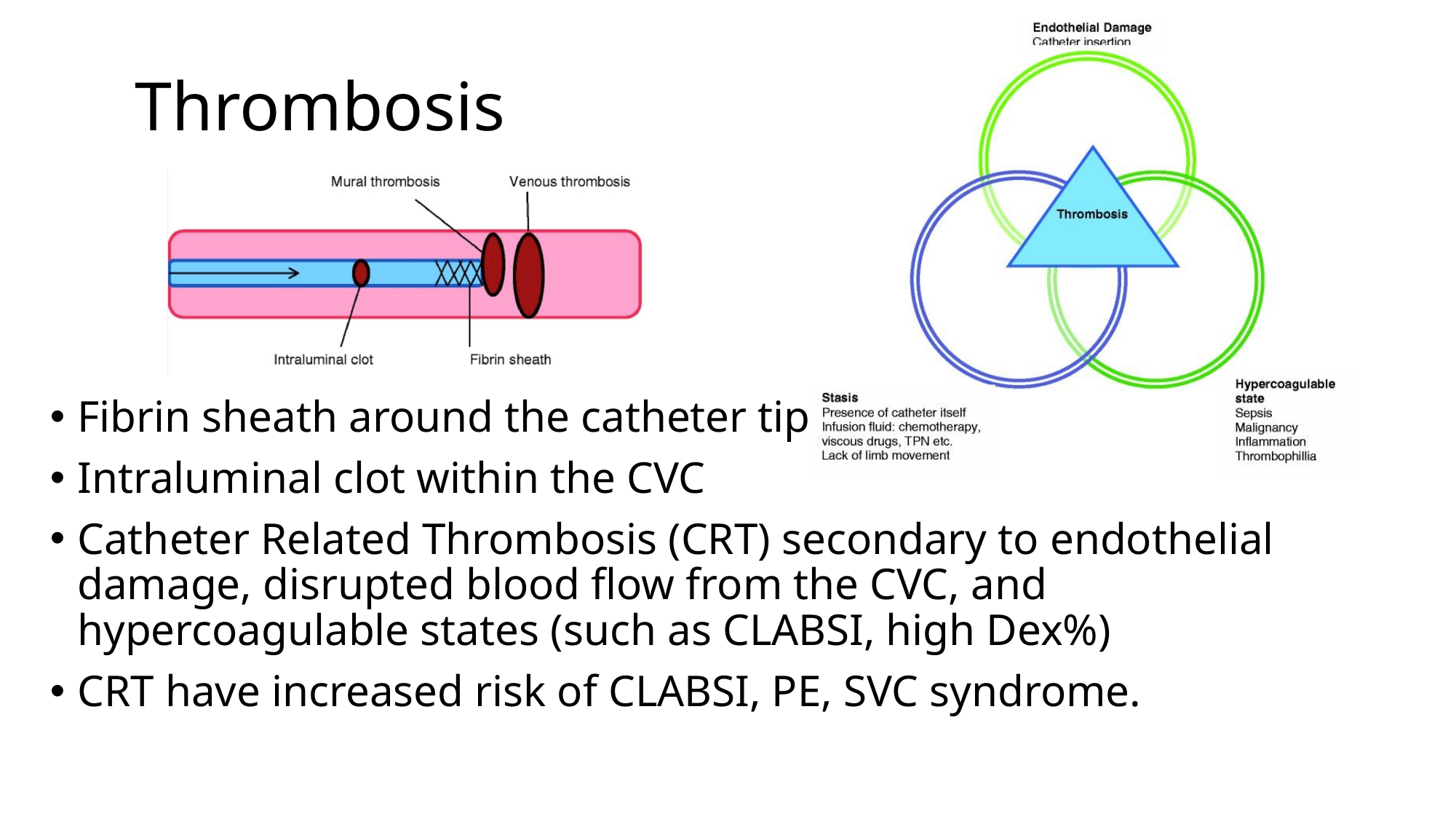

# Thrombosis
Fibrin sheath around the catheter tip
Intraluminal clot within the CVC
Catheter Related Thrombosis (CRT) secondary to endothelial damage, disrupted blood flow from the CVC, and hypercoagulable states (such as CLABSI, high Dex%)
CRT have increased risk of CLABSI, PE, SVC syndrome.

## Slide 31
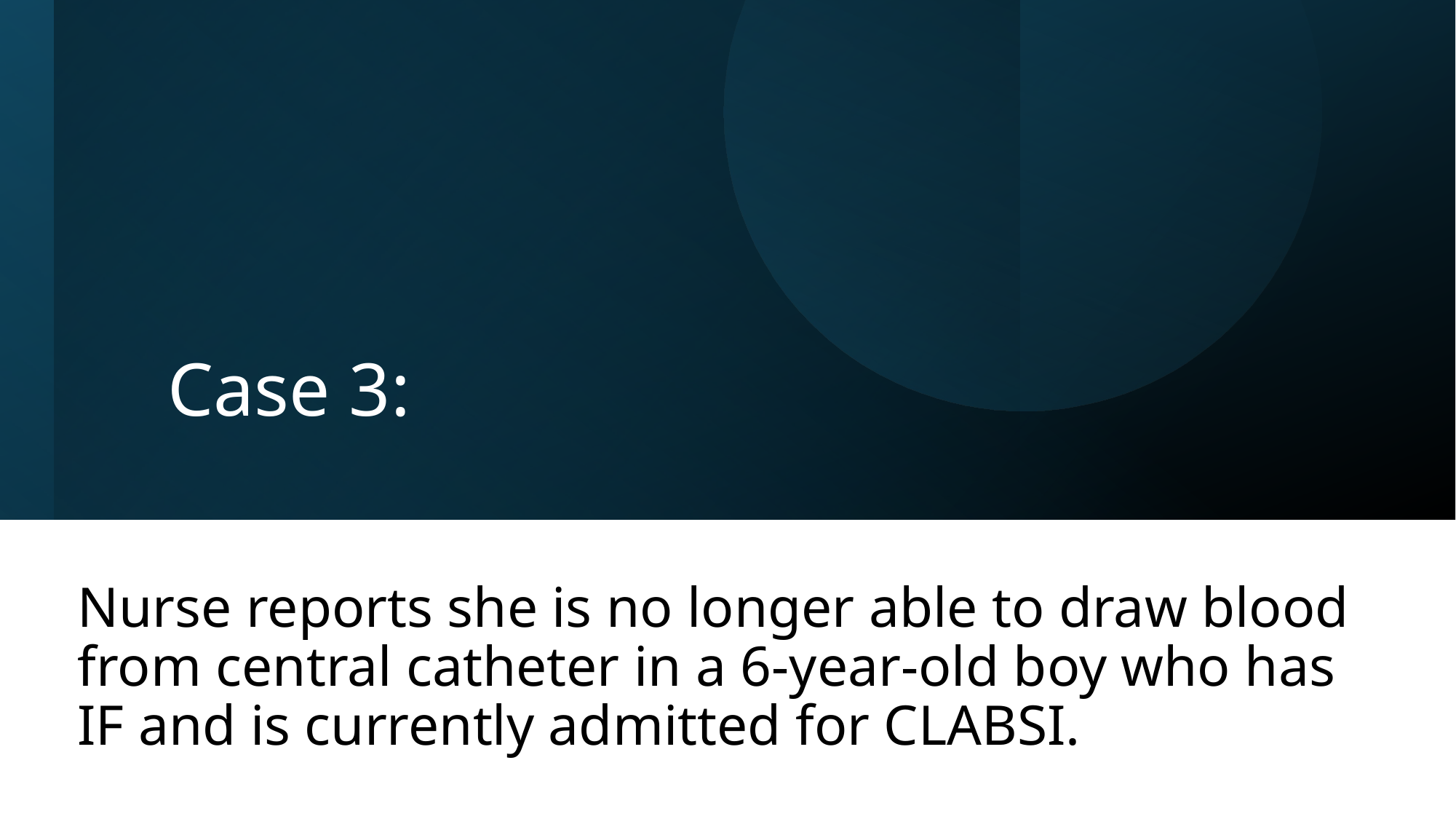

# Case 3:
Nurse reports she is no longer able to draw blood from central catheter in a 6-year-old boy who has IF and is currently admitted for CLABSI.

## Slide 32
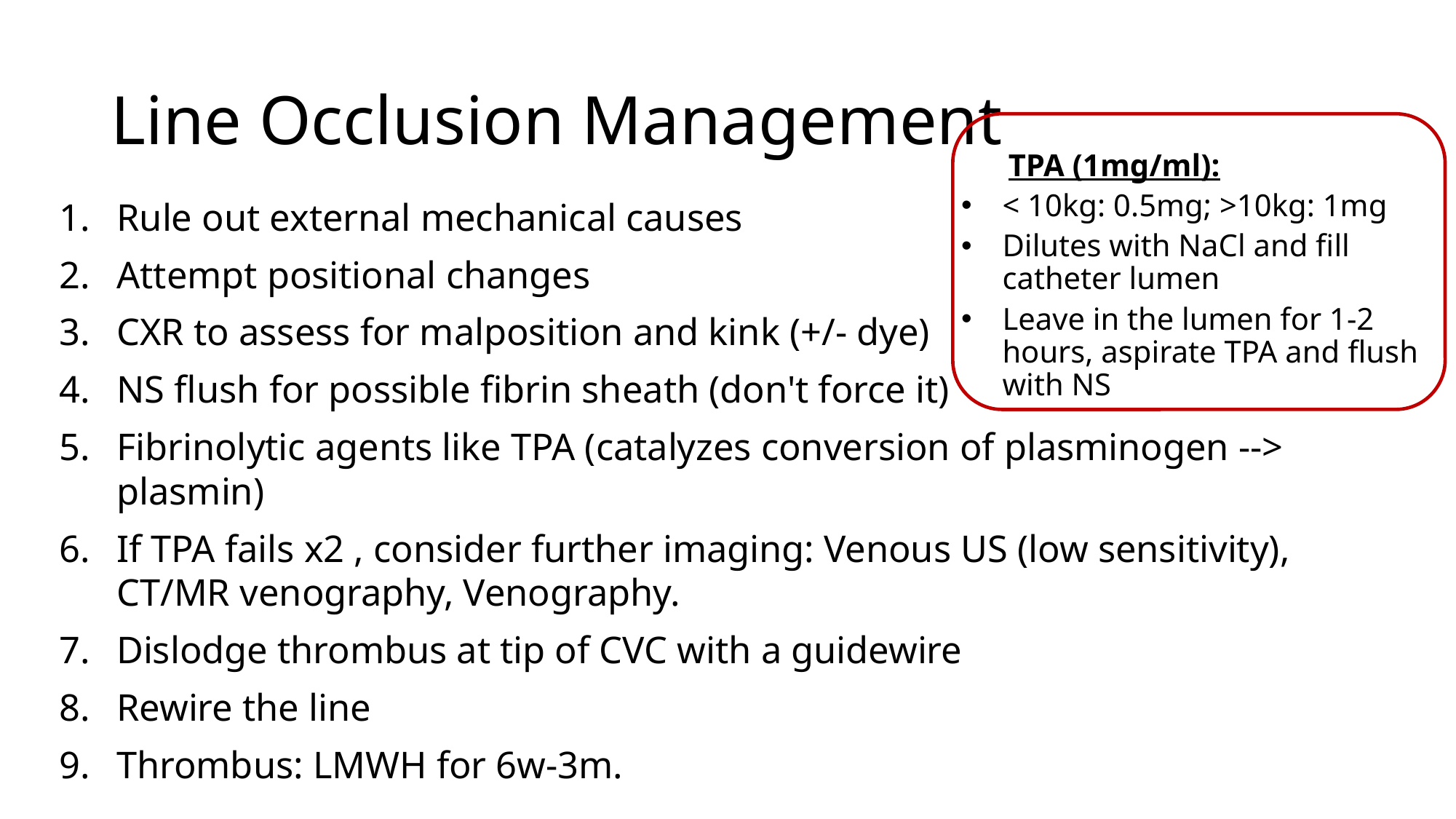

# Line Occlusion Management
 TPA (1mg/ml):
< 10kg: 0.5mg; >10kg: 1mg
Dilutes with NaCl and fill catheter lumen
Leave in the lumen for 1-2 hours, aspirate TPA and flush with NS
Rule out external mechanical causes
Attempt positional changes
CXR to assess for malposition and kink (+/- dye)
NS flush for possible fibrin sheath (don't force it)
Fibrinolytic agents like TPA (catalyzes conversion of plasminogen --> plasmin)
If TPA fails x2 , consider further imaging: Venous US (low sensitivity), CT/MR venography, Venography.
Dislodge thrombus at tip of CVC with a guidewire
Rewire the line
Thrombus: LMWH for 6w-3m.

## Slide 33
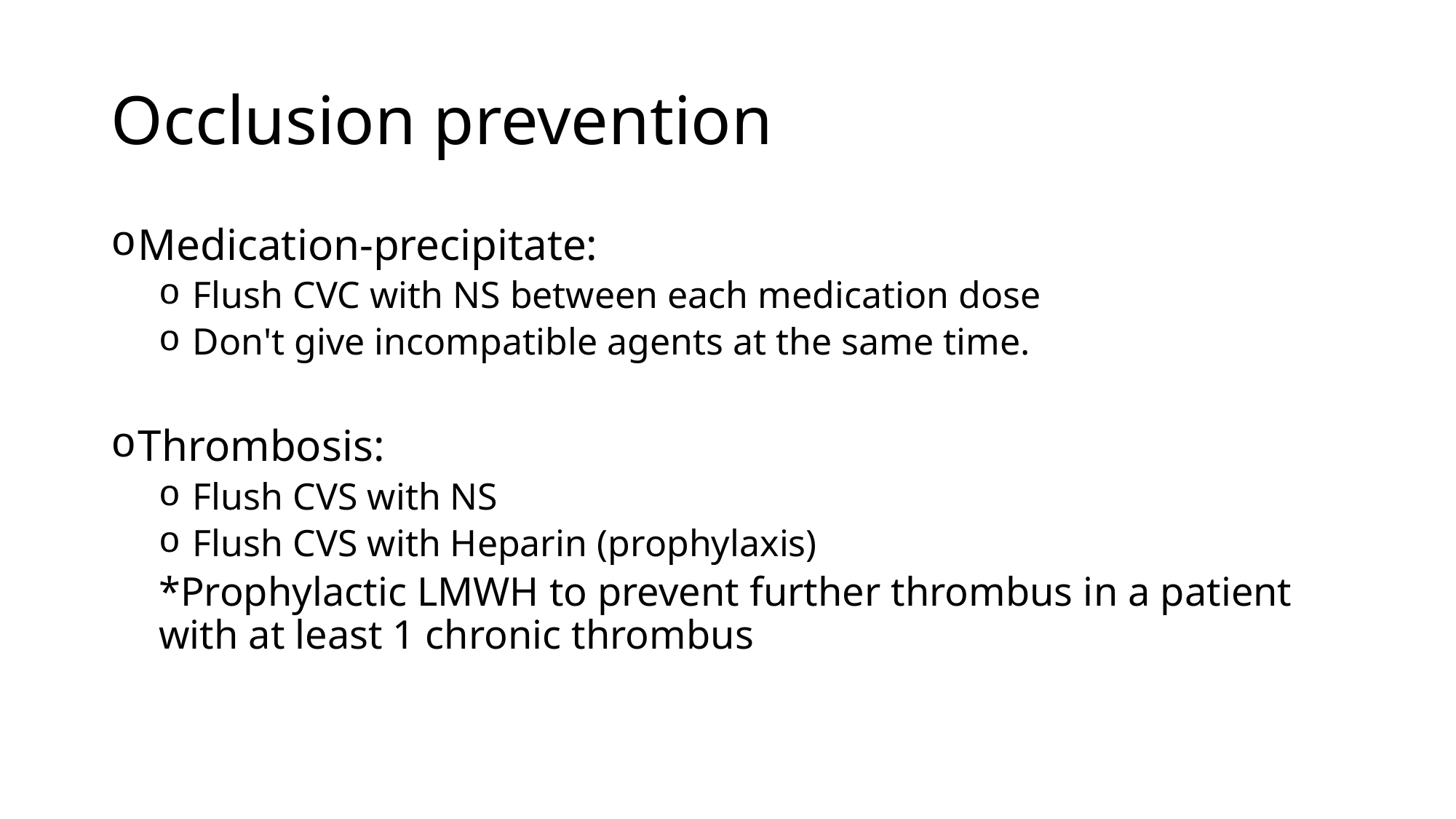

# Occlusion prevention
Medication-precipitate:
Flush CVC with NS between each medication dose
Don't give incompatible agents at the same time.
Thrombosis:
Flush CVS with NS
Flush CVS with Heparin (prophylaxis)
*Prophylactic LMWH to prevent further thrombus in a patient with at least 1 chronic thrombus

## Slide 34
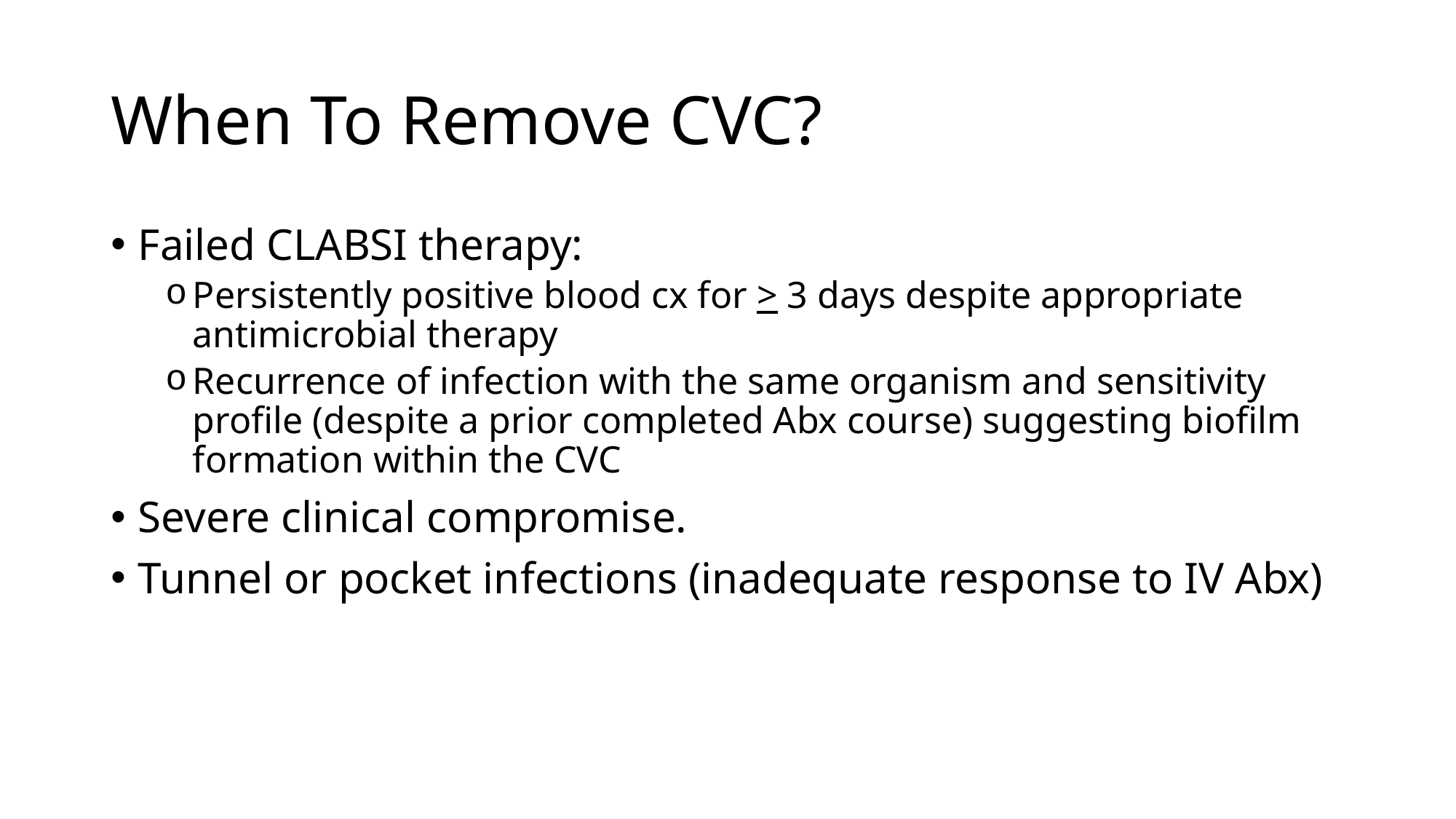

# When To Remove CVC?
Failed CLABSI therapy:
Persistently positive blood cx for > 3 days despite appropriate antimicrobial therapy
Recurrence of infection with the same organism and sensitivity profile (despite a prior completed Abx course) suggesting biofilm formation within the CVC
Severe clinical compromise.
Tunnel or pocket infections (inadequate response to IV Abx)

## Slide 35
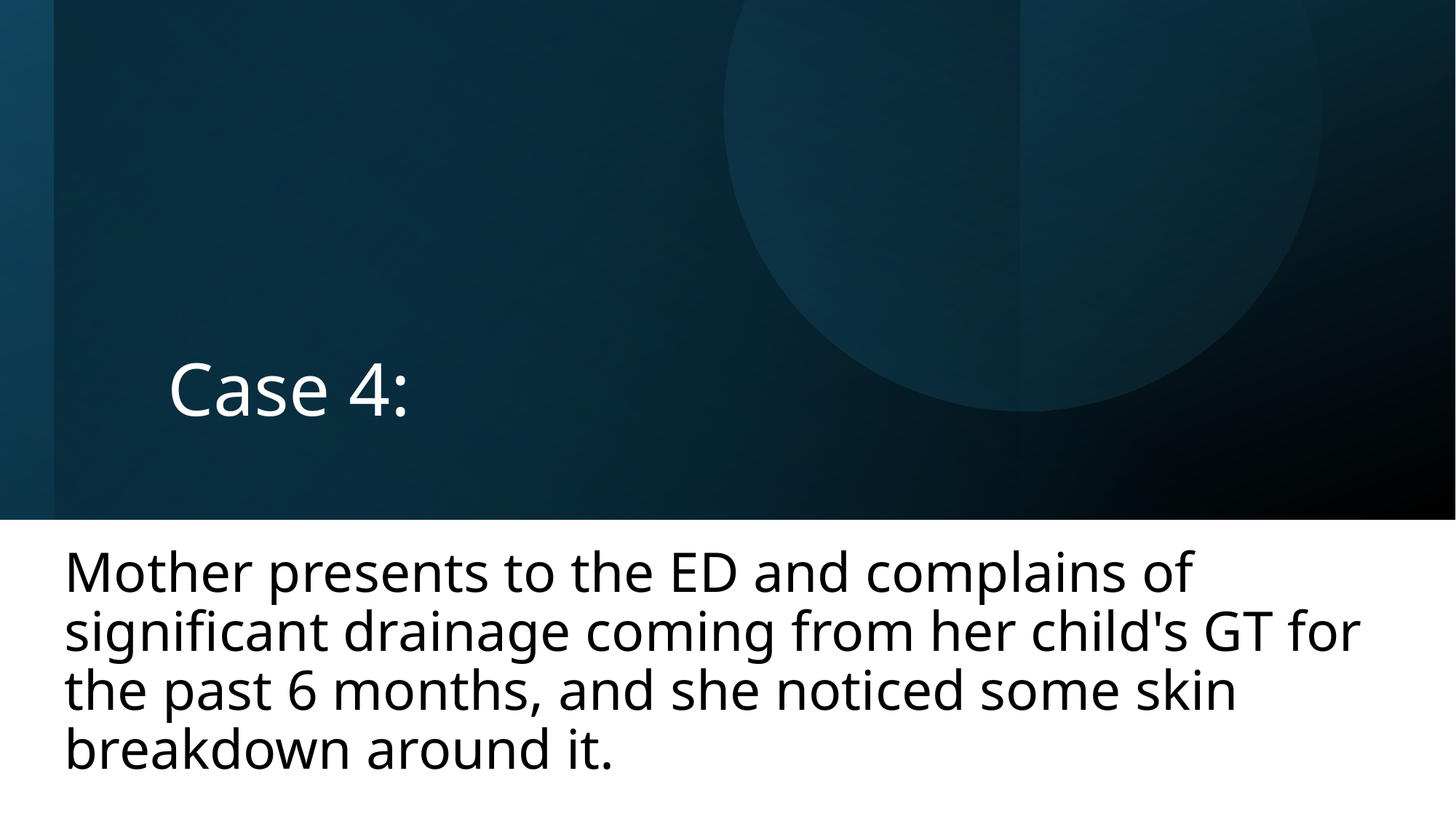

# Case 4:
Mother presents to the ED and complains of significant drainage coming from her child's GT for the past 6 months, and she noticed some skin breakdown around it.

## Slide 36
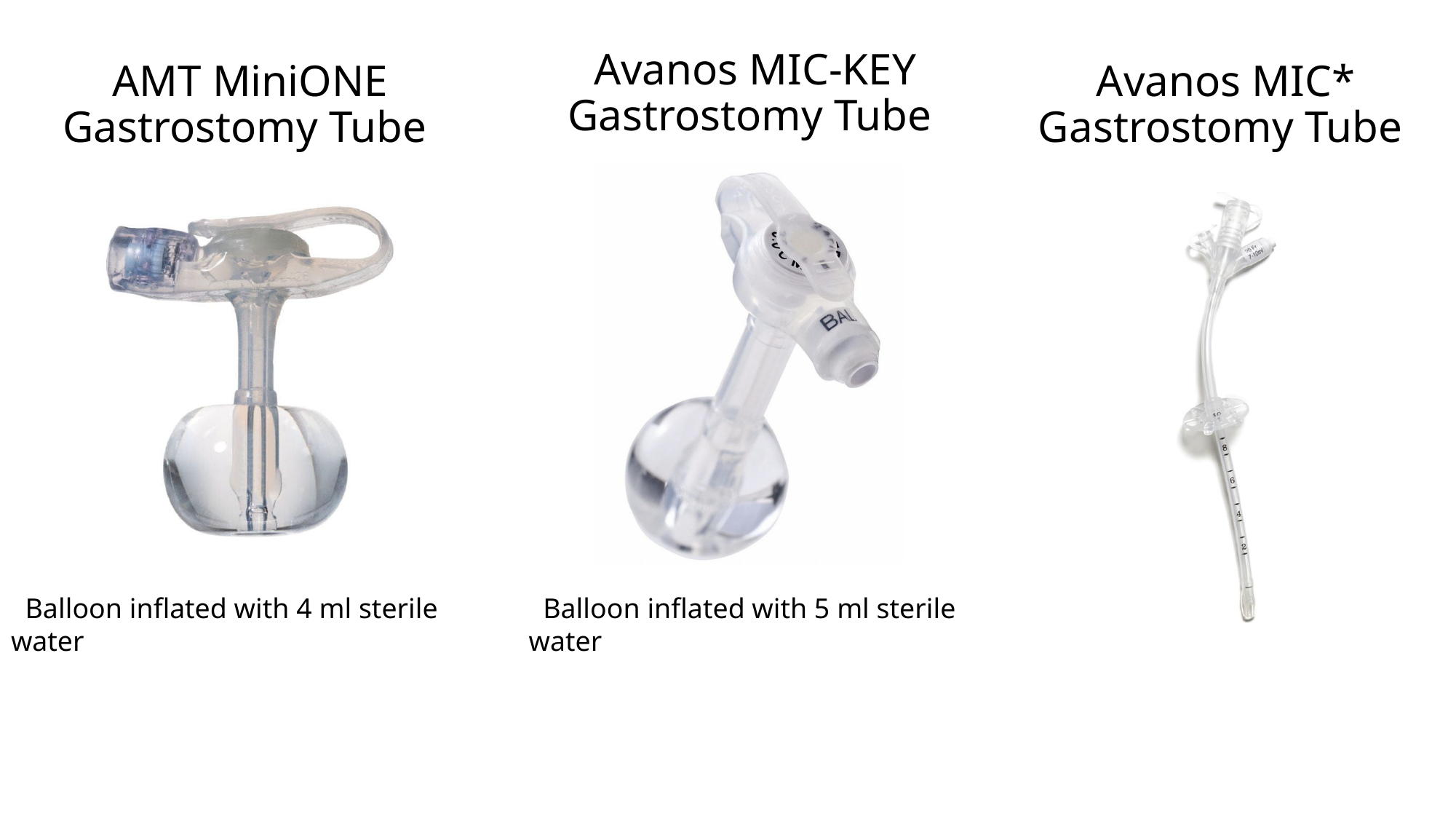

# Avanos MIC-KEY Gastrostomy Tube
Avanos MIC* Gastrostomy Tube
AMT MiniONE Gastrostomy Tube
 Balloon inflated with 4 ml sterile water
 Balloon inflated with 5 ml sterile water

## Slide 37
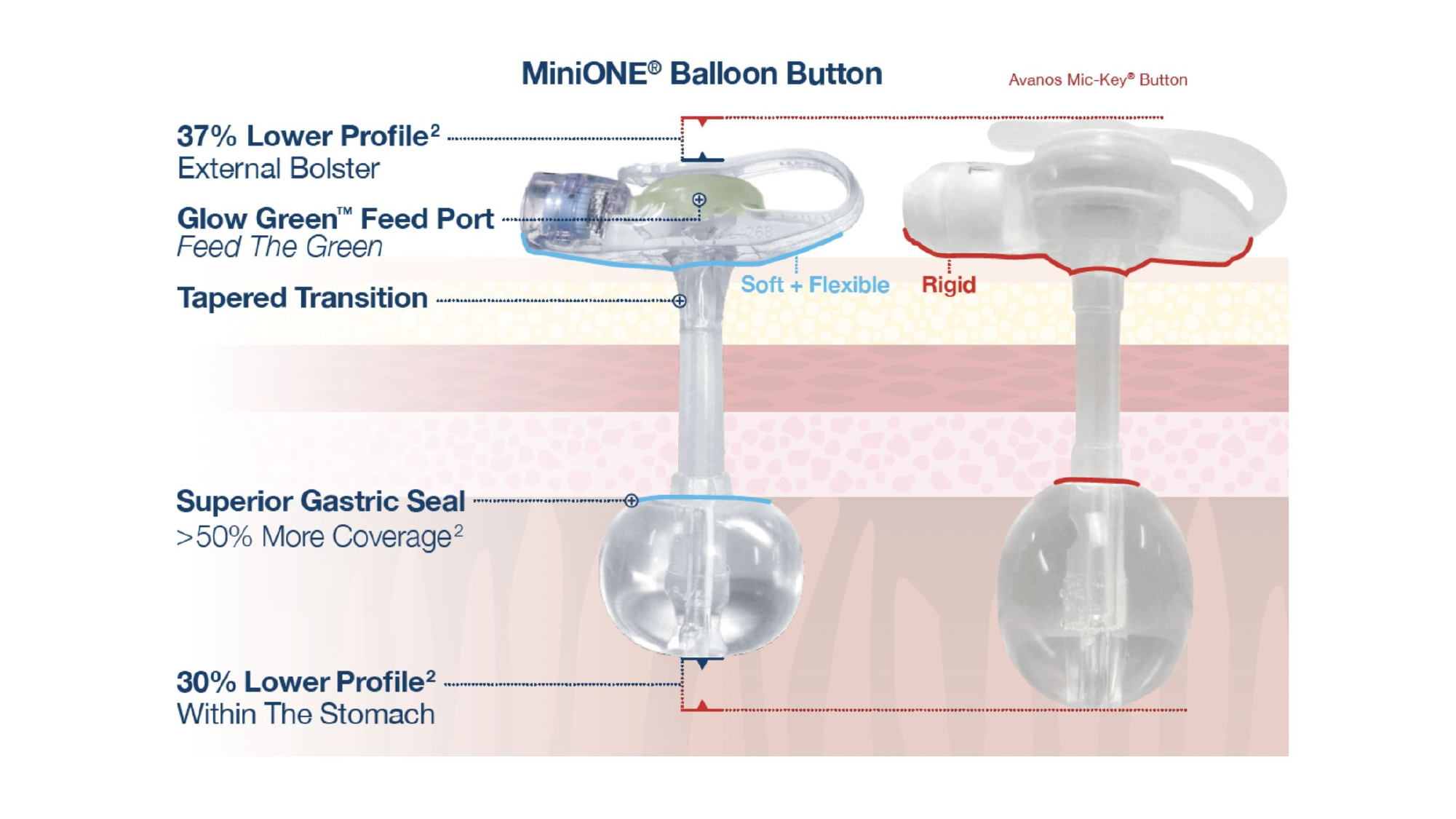

## Slide 38
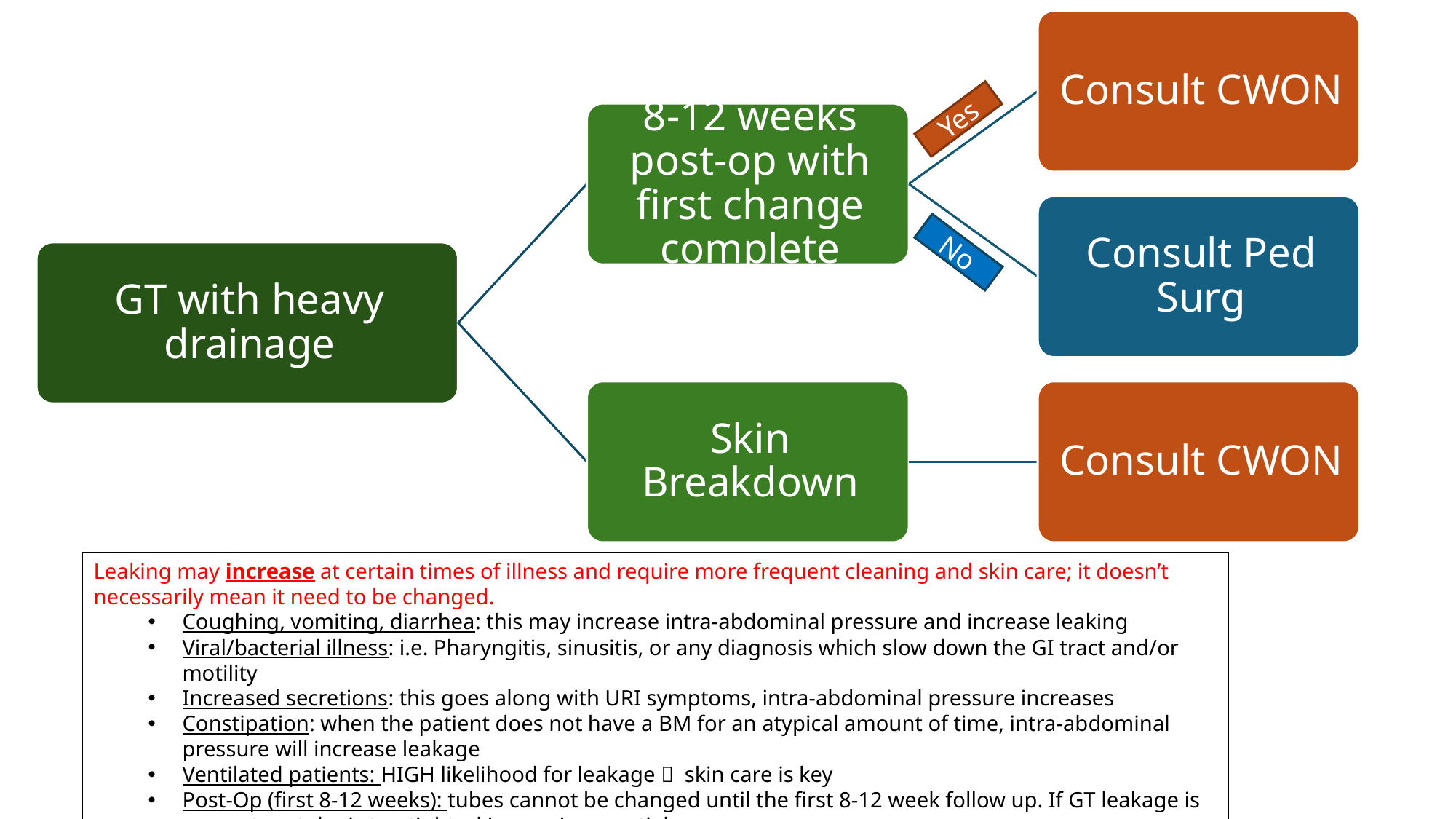

Yes
No
Leaking may increase at certain times of illness and require more frequent cleaning and skin care; it doesn’t necessarily mean it need to be changed.
Coughing, vomiting, diarrhea: this may increase intra-abdominal pressure and increase leaking
Viral/bacterial illness: i.e. Pharyngitis, sinusitis, or any diagnosis which slow down the GI tract and/or motility
Increased secretions: this goes along with URI symptoms, intra-abdominal pressure increases
Constipation: when the patient does not have a BM for an atypical amount of time, intra-abdominal pressure will increase leakage
Ventilated patients: HIGH likelihood for leakage  skin care is key
Post-Op (first 8-12 weeks): tubes cannot be changed until the first 8-12 week follow up. If GT leakage is present, or tube is too tight, skin care is essential

## Slide 39
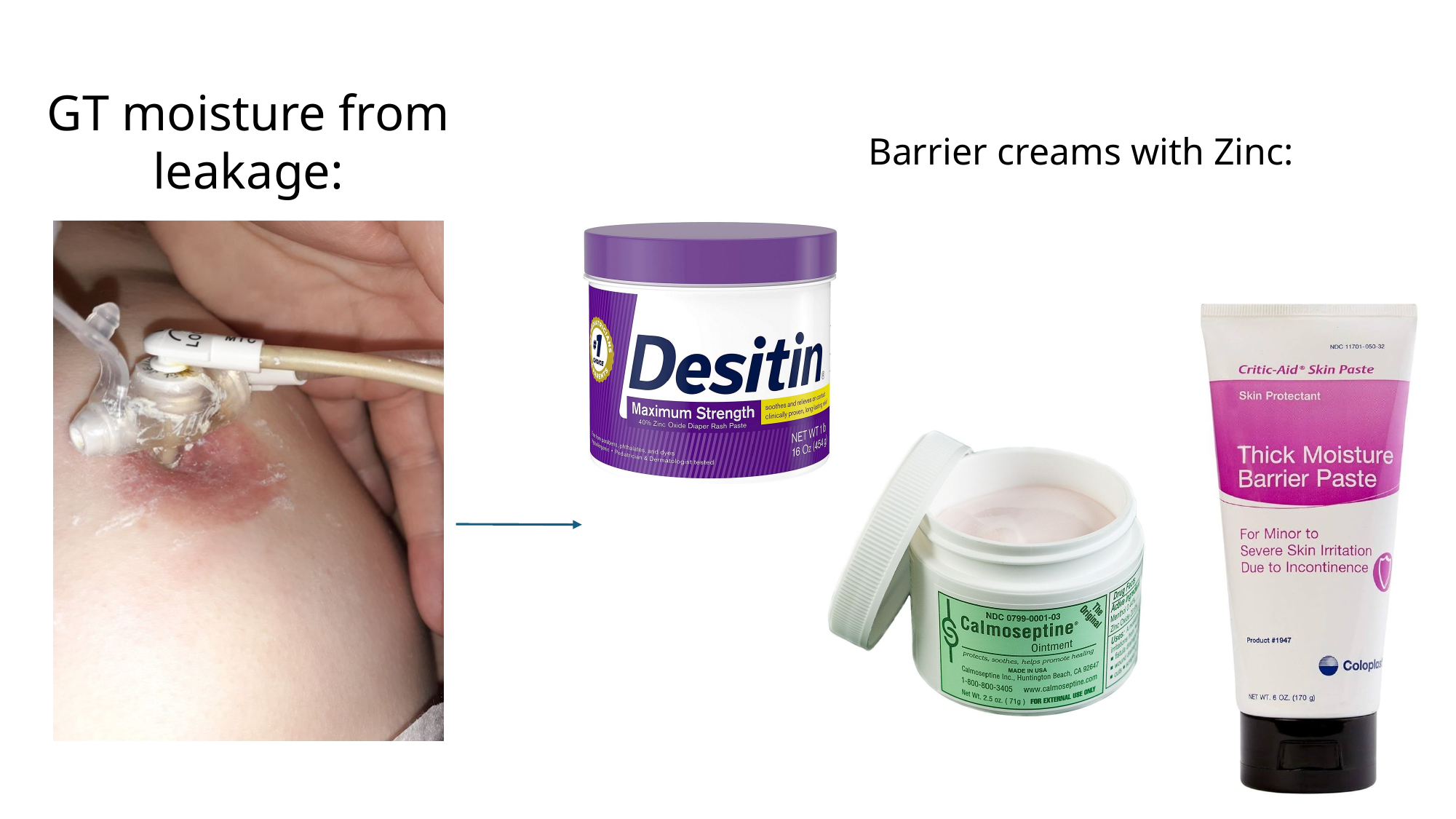

GT moisture from leakage:
Barrier creams with Zinc:

## Slide 40
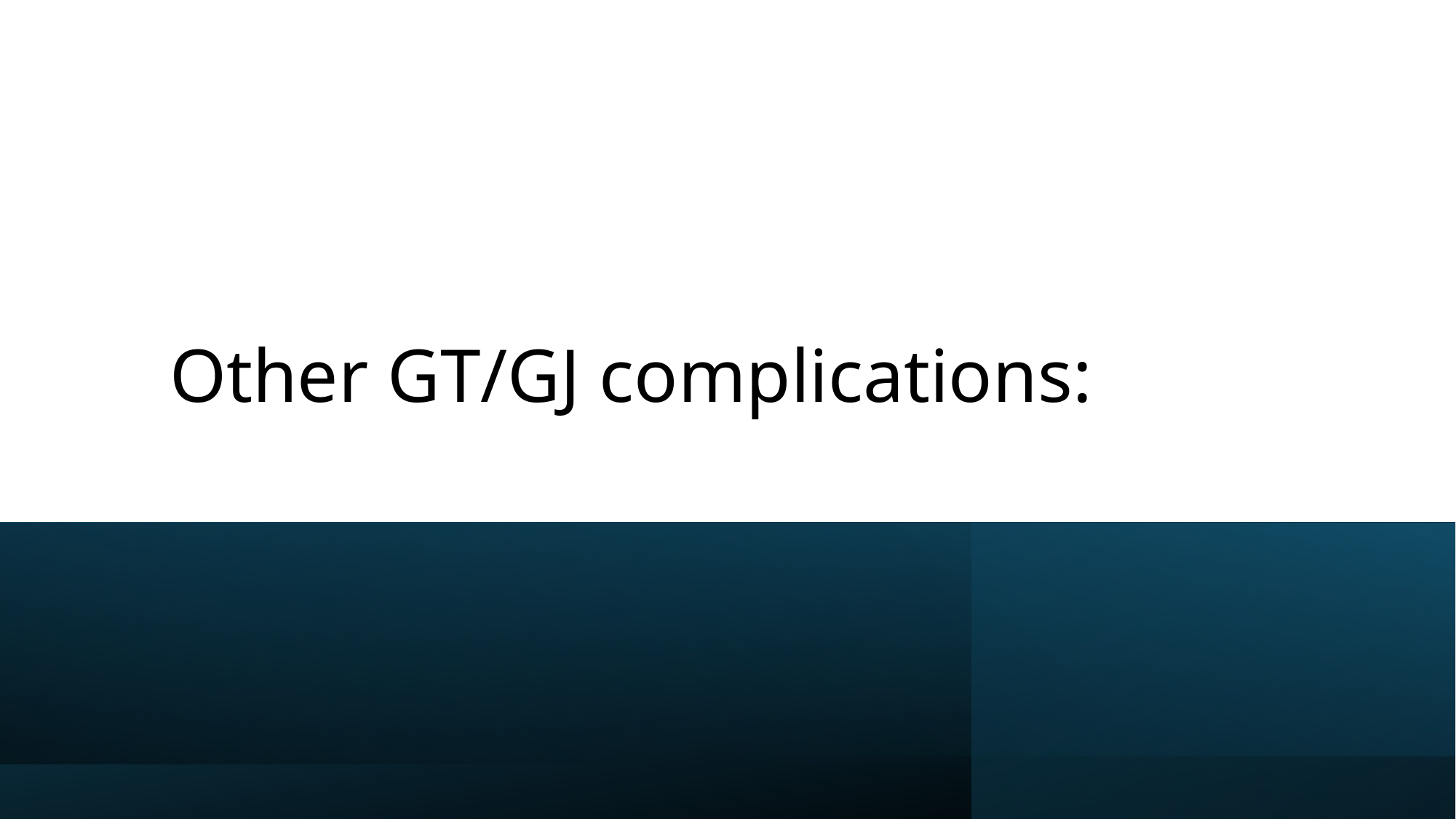

# Other GT/GJ complications:

## Slide 41
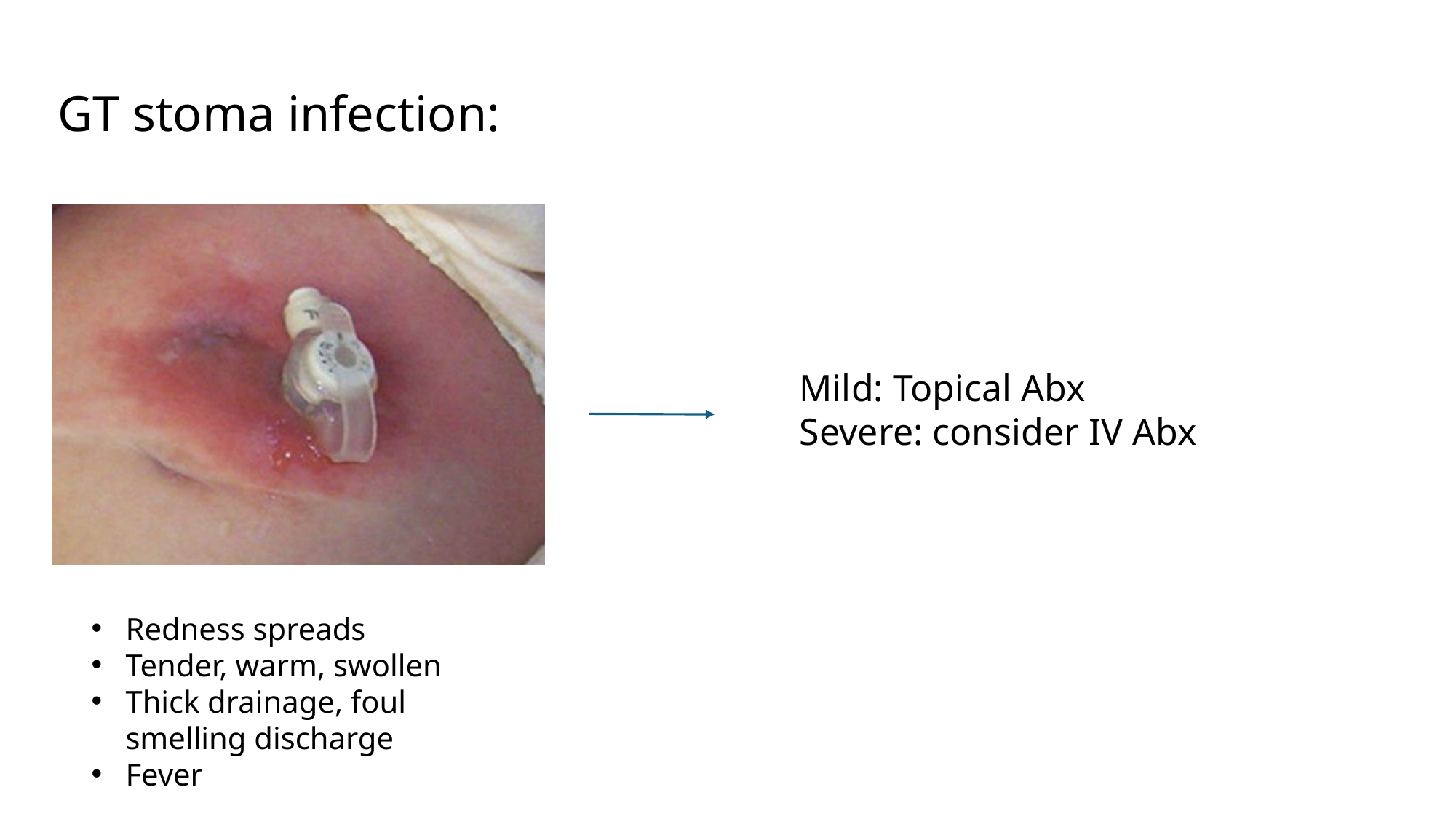

GT stoma infection:
Mild: Topical Abx
Severe: consider IV Abx
Redness spreads
Tender, warm, swollen
Thick drainage, foul smelling discharge
Fever

## Slide 42
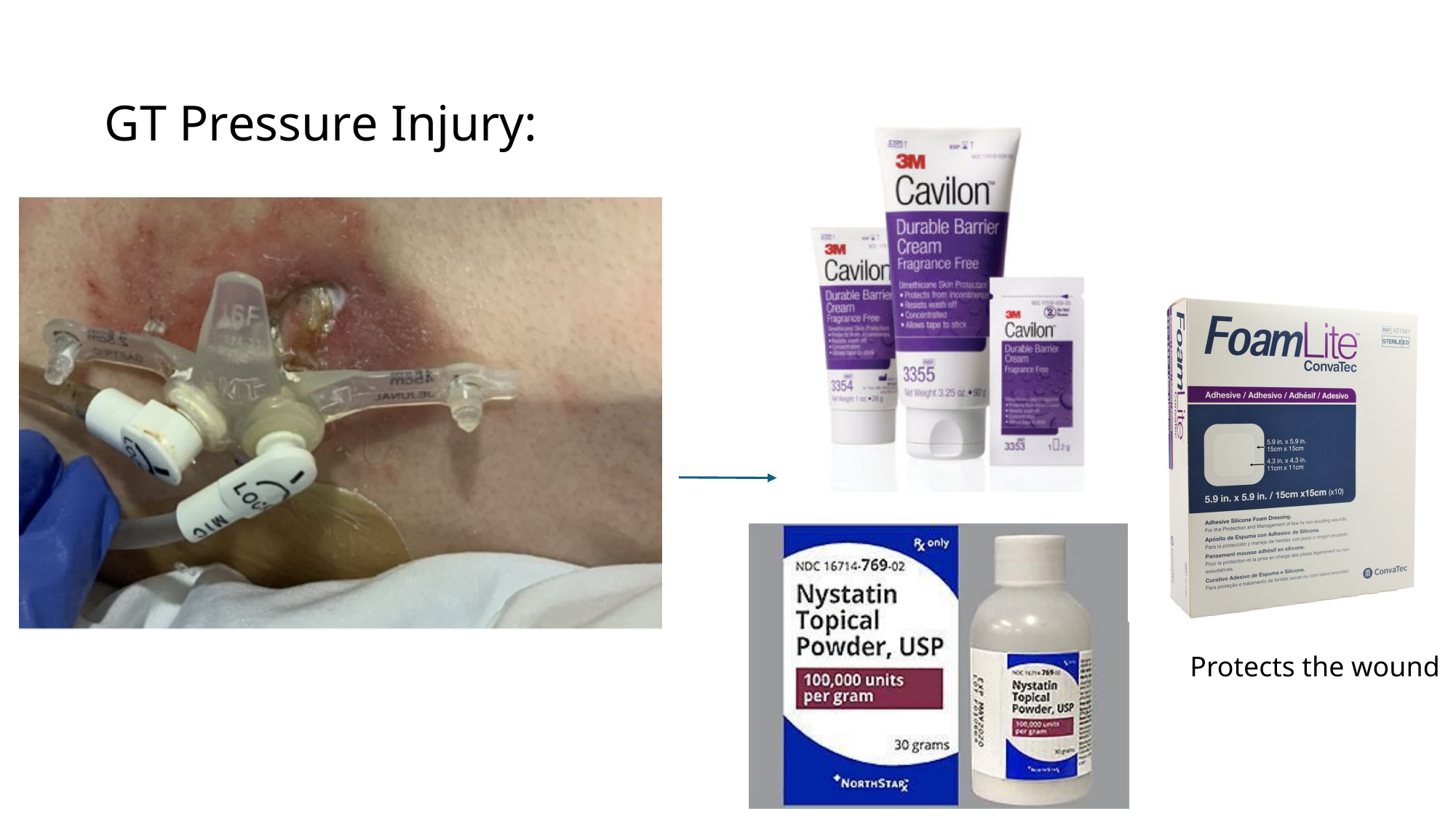

GT Pressure Injury:
Protects the wound

## Slide 43
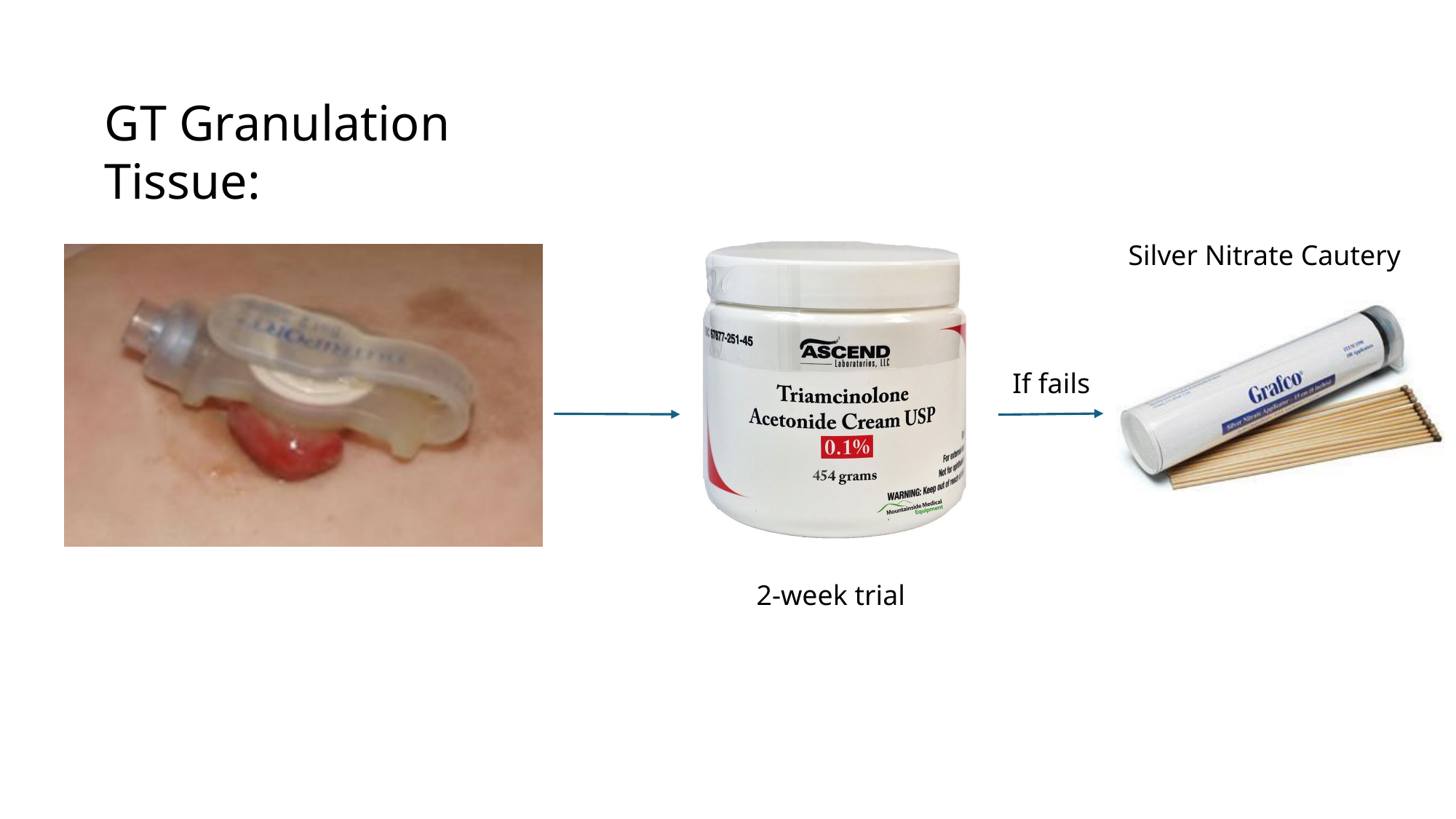

GT Granulation Tissue:
Silver Nitrate Cautery
If fails
2-week trial

## Slide 44
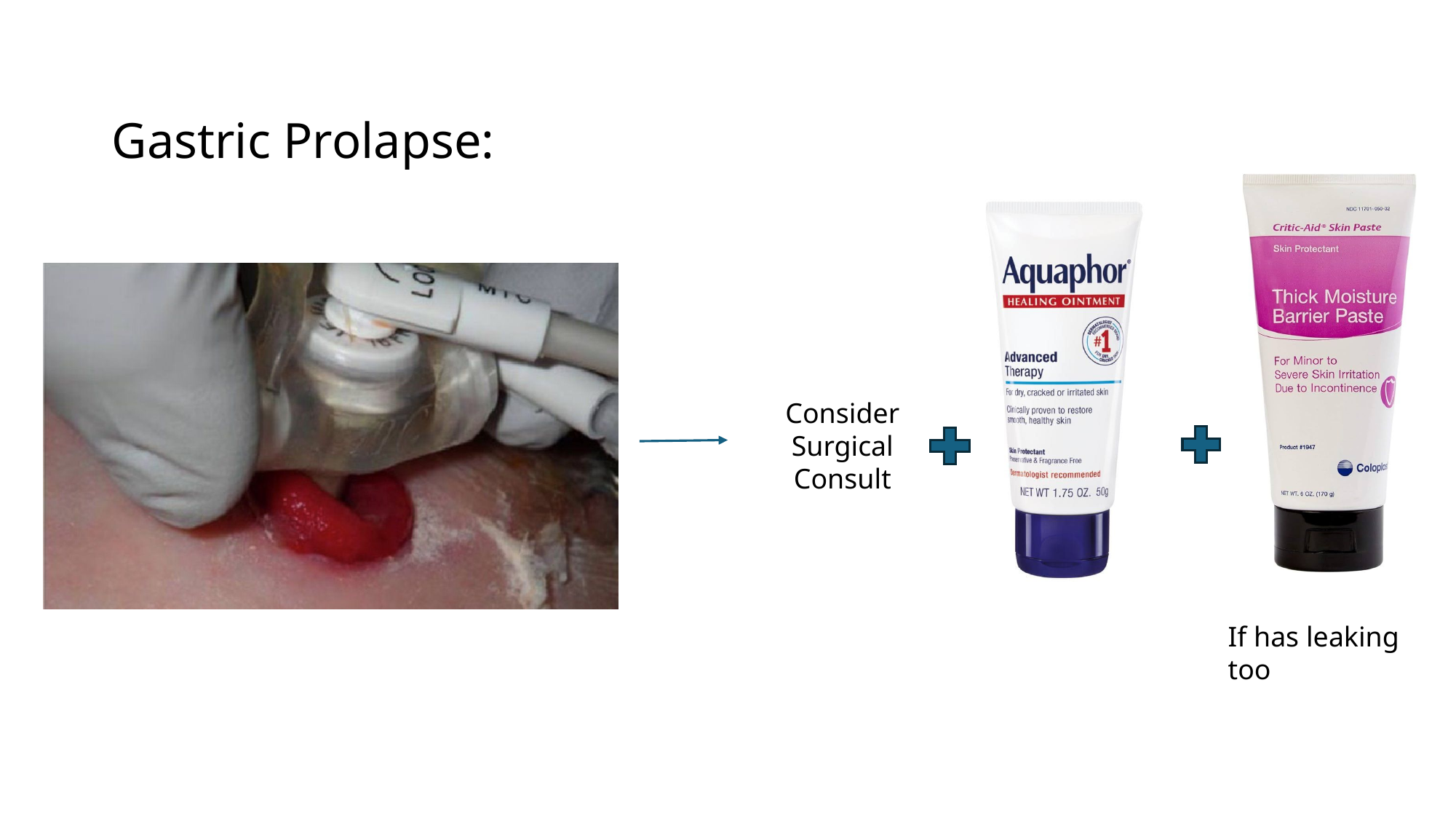

Gastric Prolapse:
Consider Surgical Consult
If has leaking too

## Slide 45
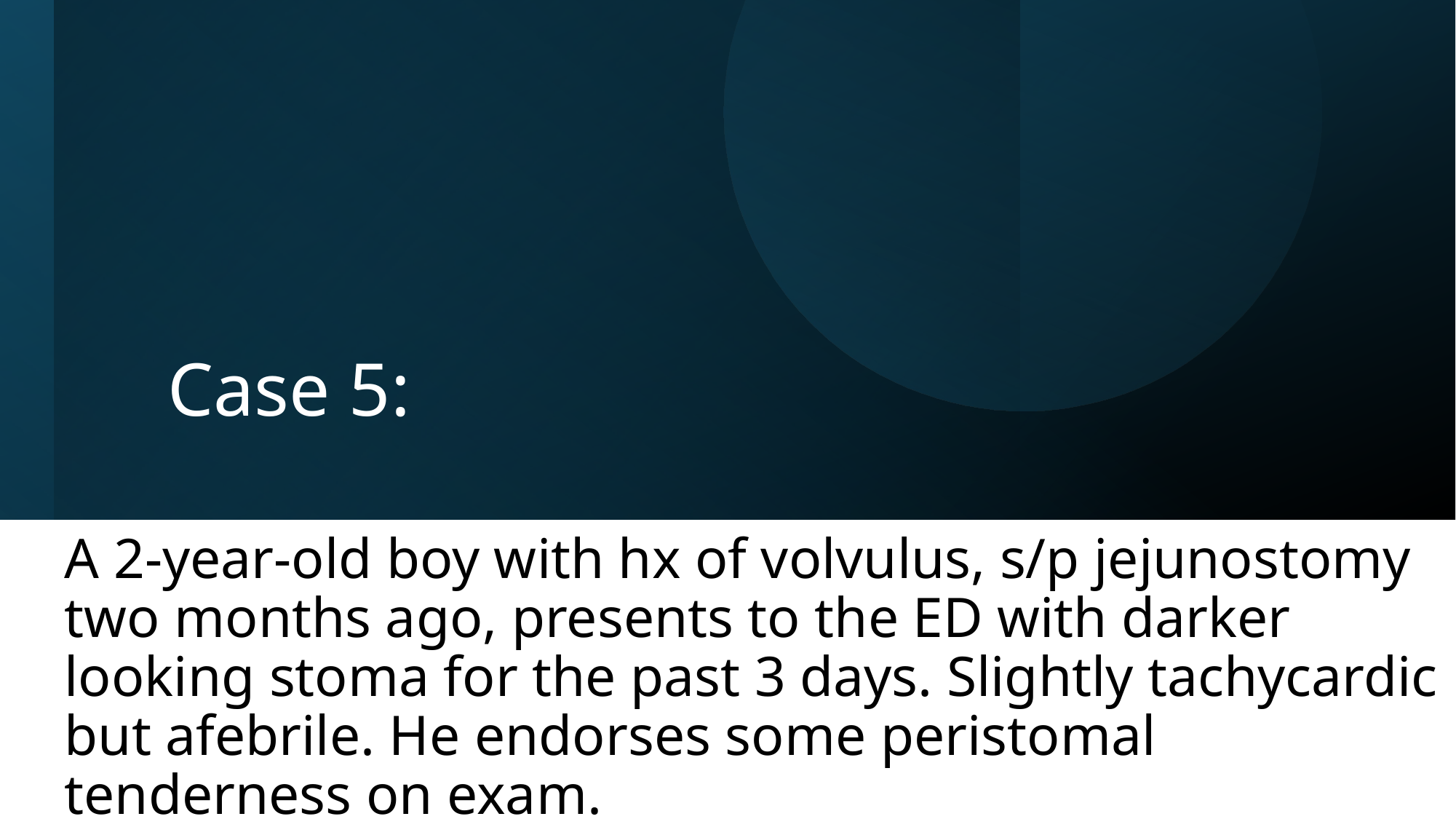

# Case 5:
A 2-year-old boy with hx of volvulus, s/p jejunostomy two months ago, presents to the ED with darker looking stoma for the past 3 days. Slightly tachycardic but afebrile. He endorses some peristomal tenderness on exam.

## Slide 46
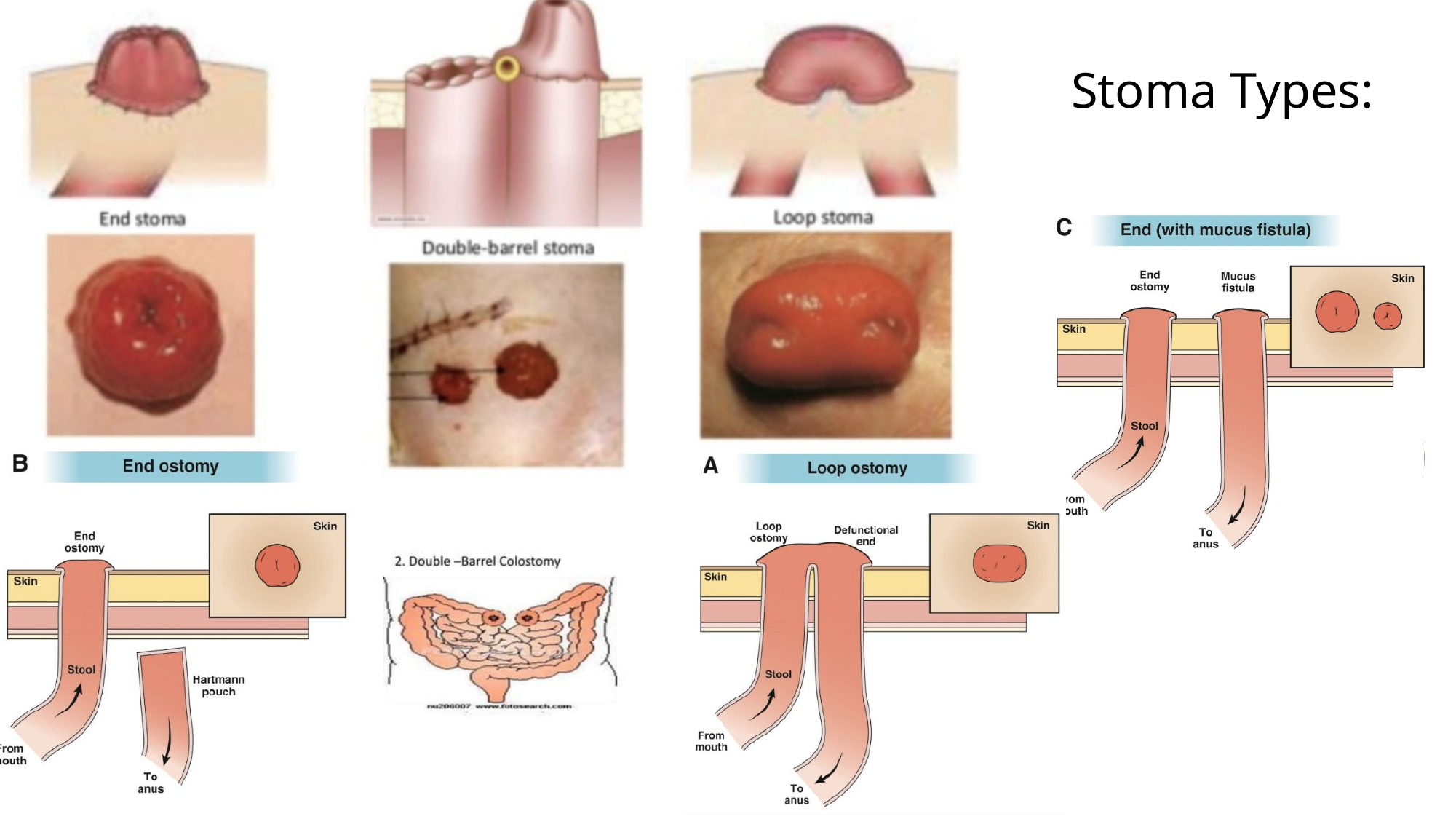

Stoma Types:

## Slide 47
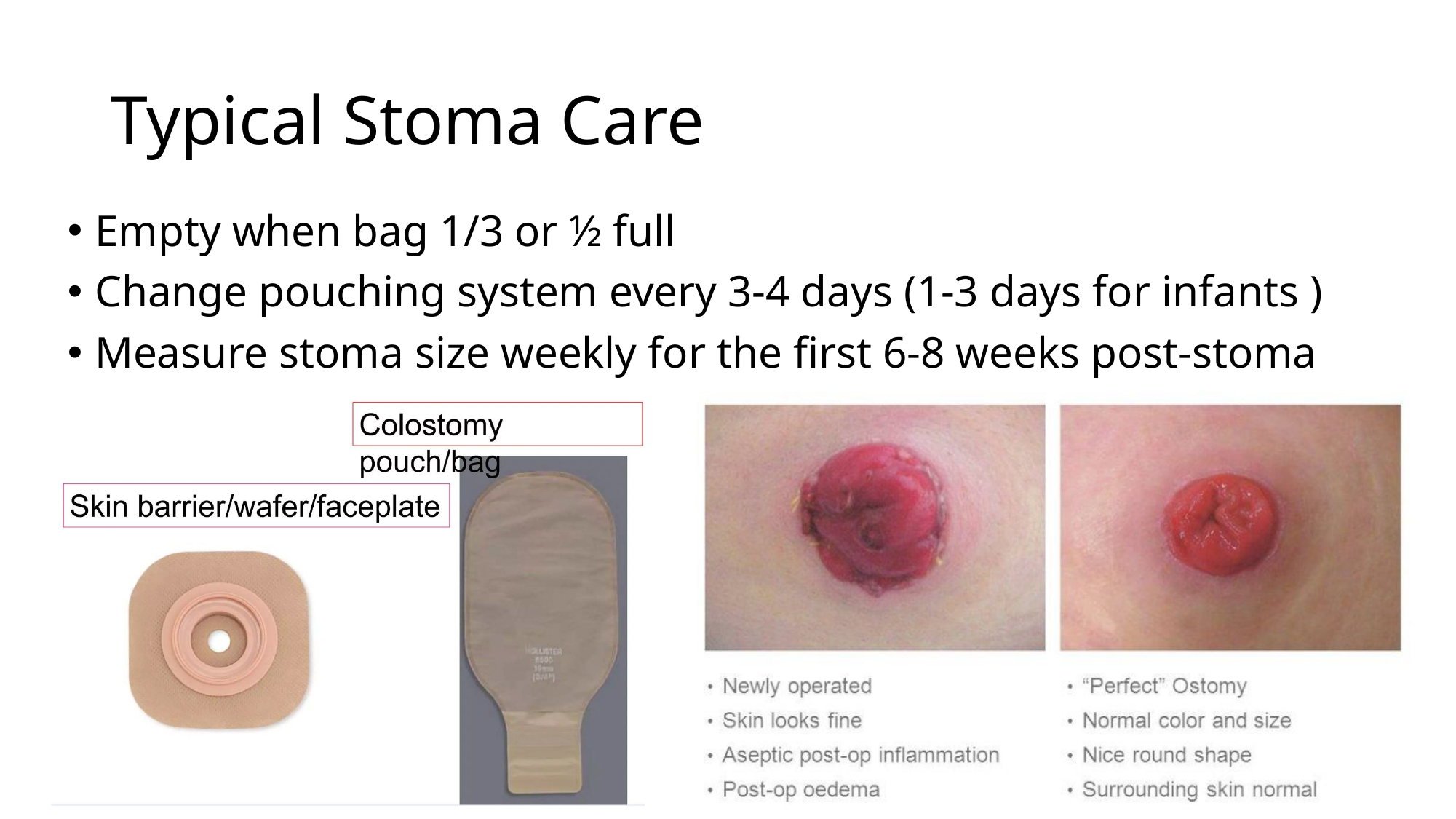

# Typical Stoma Care
Empty when bag 1/3 or ½ full
Change pouching system every 3-4 days (1-3 days for infants )
Measure stoma size weekly for the first 6-8 weeks post-stoma creation

## Slide 48
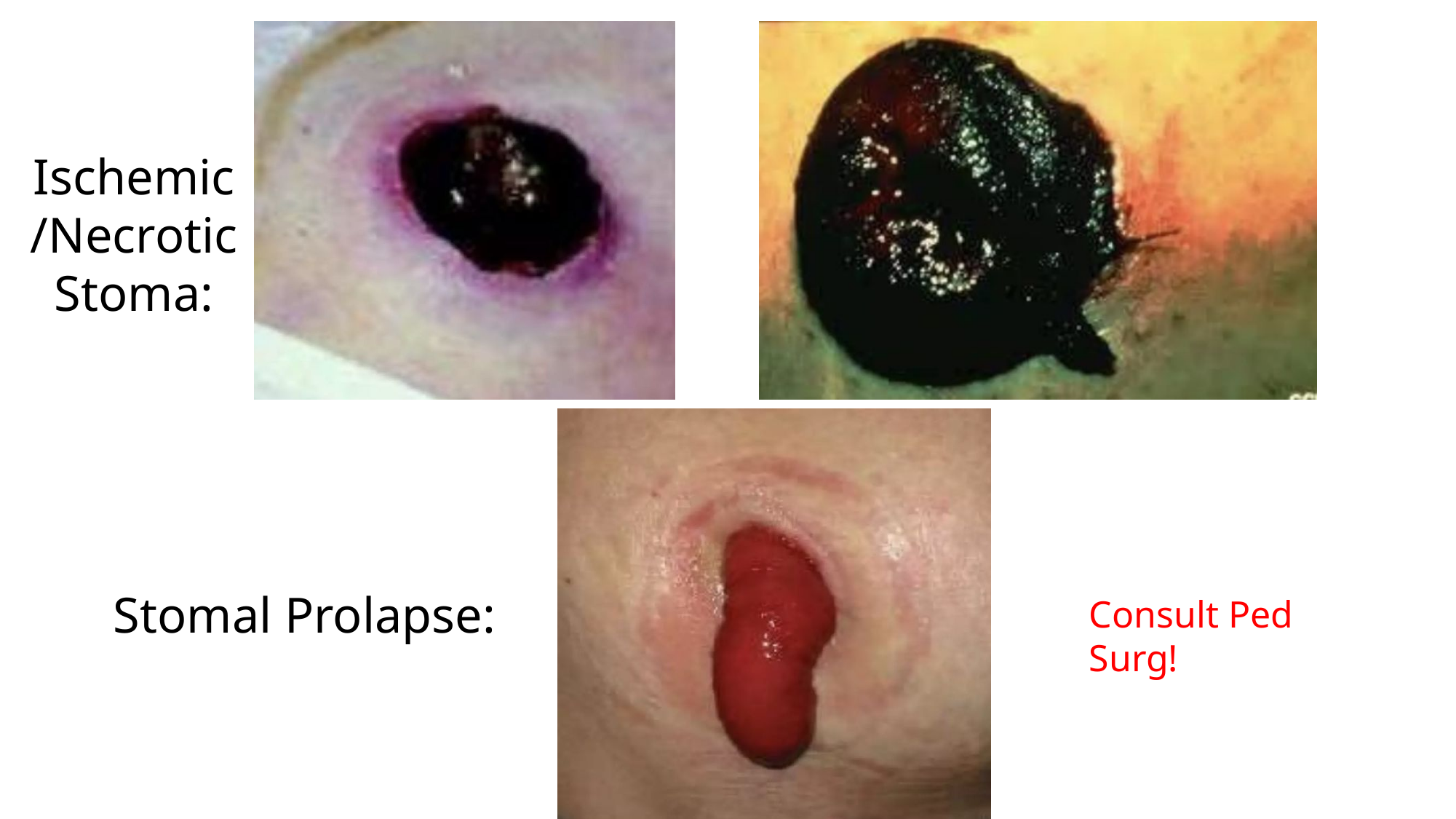

Ischemic/Necrotic Stoma:
Stomal Prolapse:
Consult Ped Surg!

## Slide 49
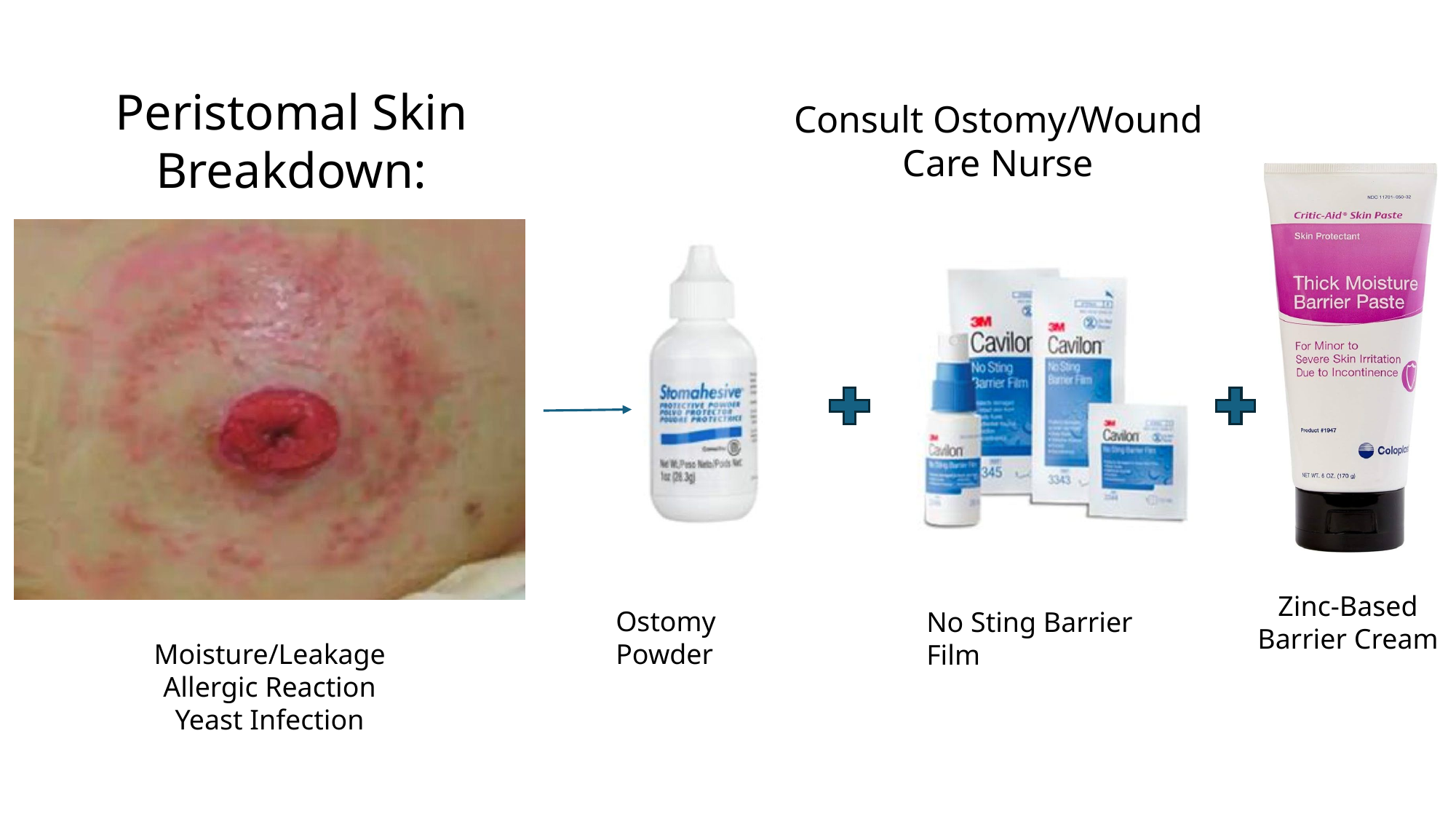

Peristomal Skin Breakdown:
Consult Ostomy/Wound Care Nurse
Zinc-Based Barrier Cream
Ostomy Powder
No Sting Barrier Film
Moisture/Leakage
Allergic Reaction
Yeast Infection

## Slide 50
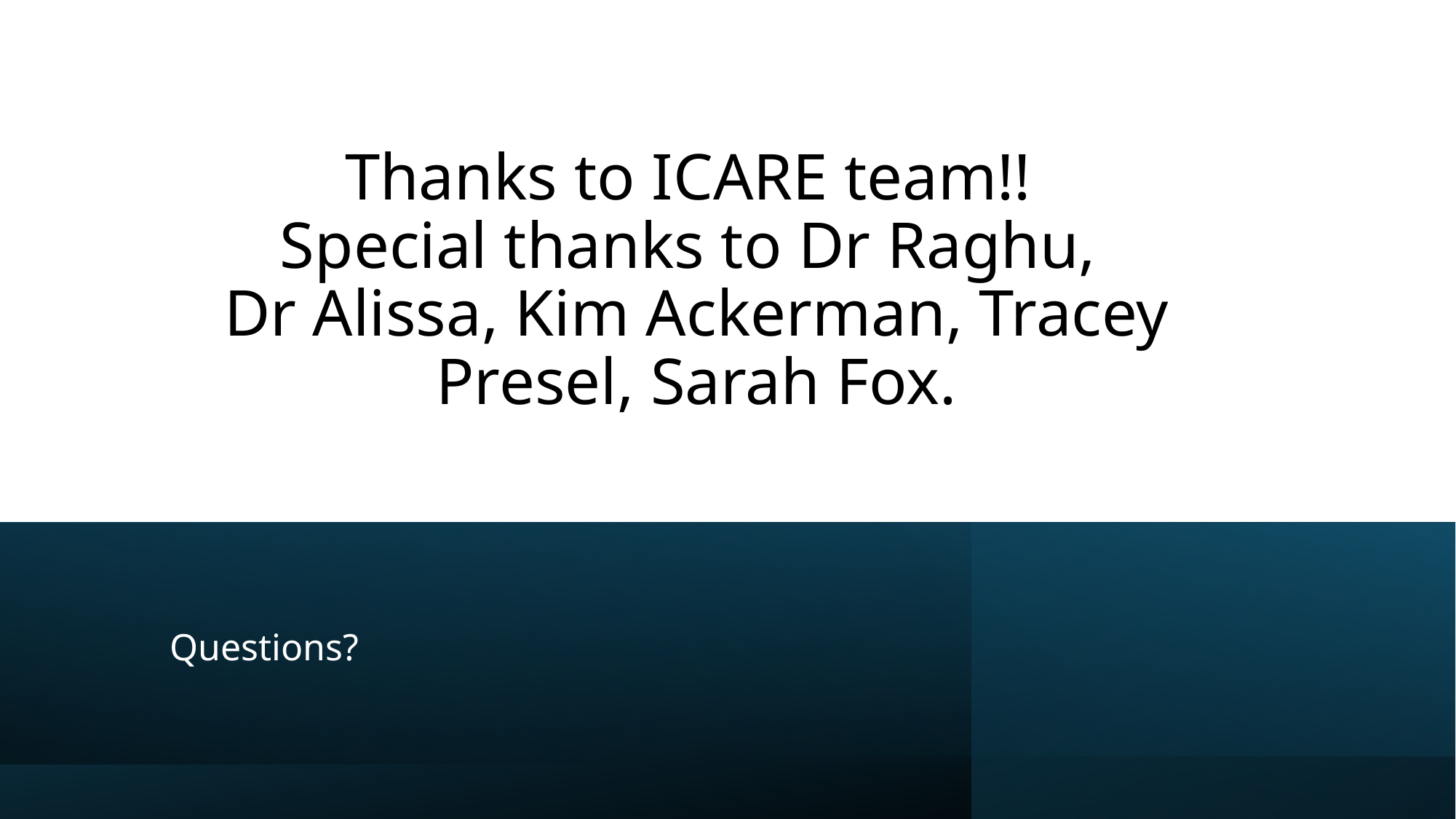

# Thanks to ICARE team!! Special thanks to Dr Raghu, Dr Alissa, Kim Ackerman, Tracey Presel, Sarah Fox.
Questions?

## Slide 51
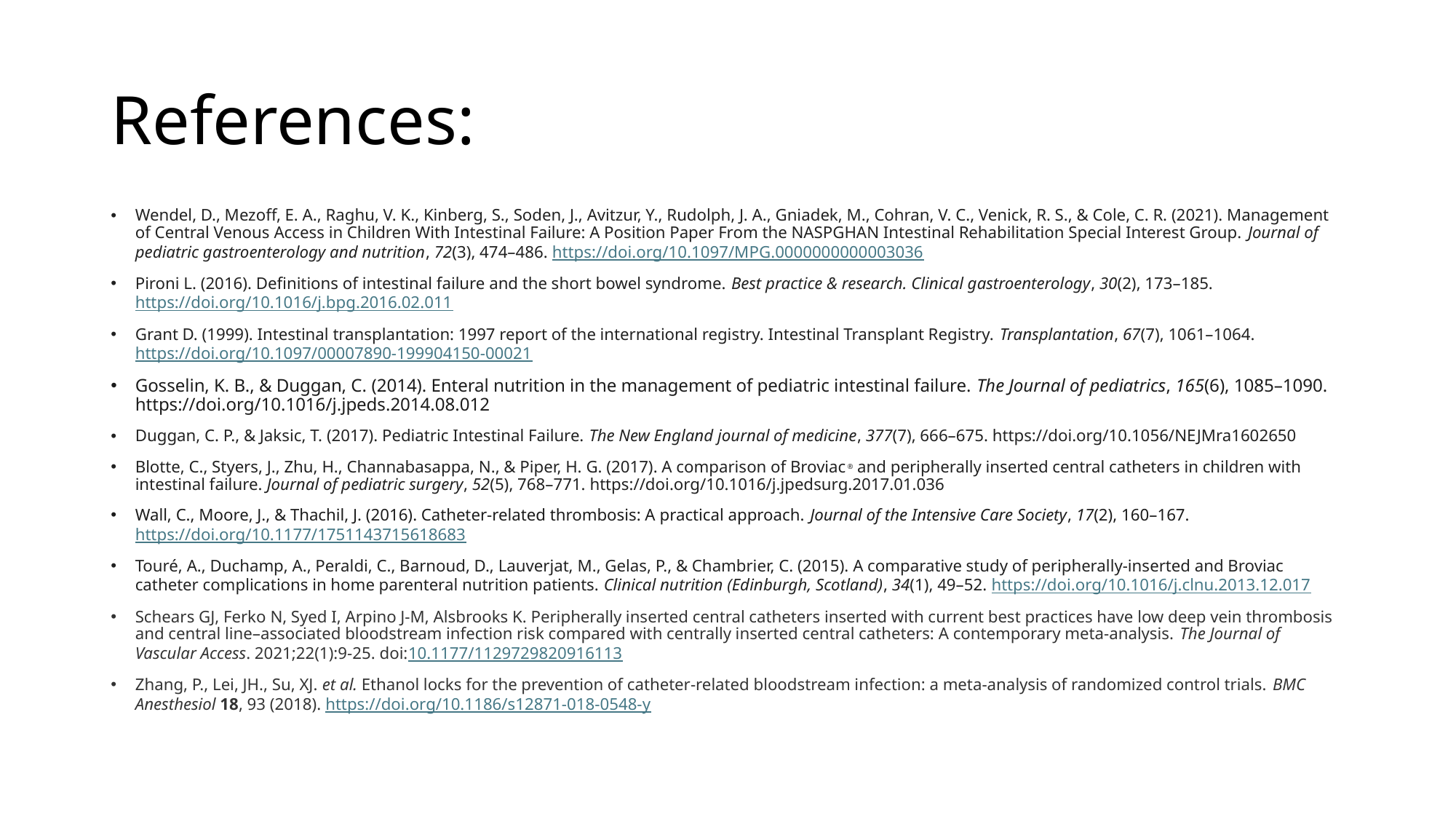

# References:
Wendel, D., Mezoff, E. A., Raghu, V. K., Kinberg, S., Soden, J., Avitzur, Y., Rudolph, J. A., Gniadek, M., Cohran, V. C., Venick, R. S., & Cole, C. R. (2021). Management of Central Venous Access in Children With Intestinal Failure: A Position Paper From the NASPGHAN Intestinal Rehabilitation Special Interest Group. Journal of pediatric gastroenterology and nutrition, 72(3), 474–486. https://doi.org/10.1097/MPG.0000000000003036
Pironi L. (2016). Definitions of intestinal failure and the short bowel syndrome. Best practice & research. Clinical gastroenterology, 30(2), 173–185. https://doi.org/10.1016/j.bpg.2016.02.011
Grant D. (1999). Intestinal transplantation: 1997 report of the international registry. Intestinal Transplant Registry. Transplantation, 67(7), 1061–1064. https://doi.org/10.1097/00007890-199904150-00021
Gosselin, K. B., & Duggan, C. (2014). Enteral nutrition in the management of pediatric intestinal failure. The Journal of pediatrics, 165(6), 1085–1090. https://doi.org/10.1016/j.jpeds.2014.08.012
Duggan, C. P., & Jaksic, T. (2017). Pediatric Intestinal Failure. The New England journal of medicine, 377(7), 666–675. https://doi.org/10.1056/NEJMra1602650
Blotte, C., Styers, J., Zhu, H., Channabasappa, N., & Piper, H. G. (2017). A comparison of Broviac® and peripherally inserted central catheters in children with intestinal failure. Journal of pediatric surgery, 52(5), 768–771. https://doi.org/10.1016/j.jpedsurg.2017.01.036
Wall, C., Moore, J., & Thachil, J. (2016). Catheter-related thrombosis: A practical approach. Journal of the Intensive Care Society, 17(2), 160–167. https://doi.org/10.1177/1751143715618683
Touré, A., Duchamp, A., Peraldi, C., Barnoud, D., Lauverjat, M., Gelas, P., & Chambrier, C. (2015). A comparative study of peripherally-inserted and Broviac catheter complications in home parenteral nutrition patients. Clinical nutrition (Edinburgh, Scotland), 34(1), 49–52. https://doi.org/10.1016/j.clnu.2013.12.017
Schears GJ, Ferko N, Syed I, Arpino J-M, Alsbrooks K. Peripherally inserted central catheters inserted with current best practices have low deep vein thrombosis and central line–associated bloodstream infection risk compared with centrally inserted central catheters: A contemporary meta-analysis. The Journal of Vascular Access. 2021;22(1):9-25. doi:10.1177/1129729820916113
Zhang, P., Lei, JH., Su, XJ. et al. Ethanol locks for the prevention of catheter-related bloodstream infection: a meta-analysis of randomized control trials. BMC Anesthesiol 18, 93 (2018). https://doi.org/10.1186/s12871-018-0548-y
