# Supplementary material for: Addressing gaps in pediatric resident education on the management of intestinal failure in the United States: Creation and implementation of a targeted curriculum
Source: Intest Fail. 2026 Apr 11;10:100368. doi: 10.1016/j.intf.2026.100368 (PMC13092194; doi:10.1016/j.intf.2026.100368)
Supplement: Supplementary file 3 — Supplementary material [file mmc3.pptx]

## Slide 1
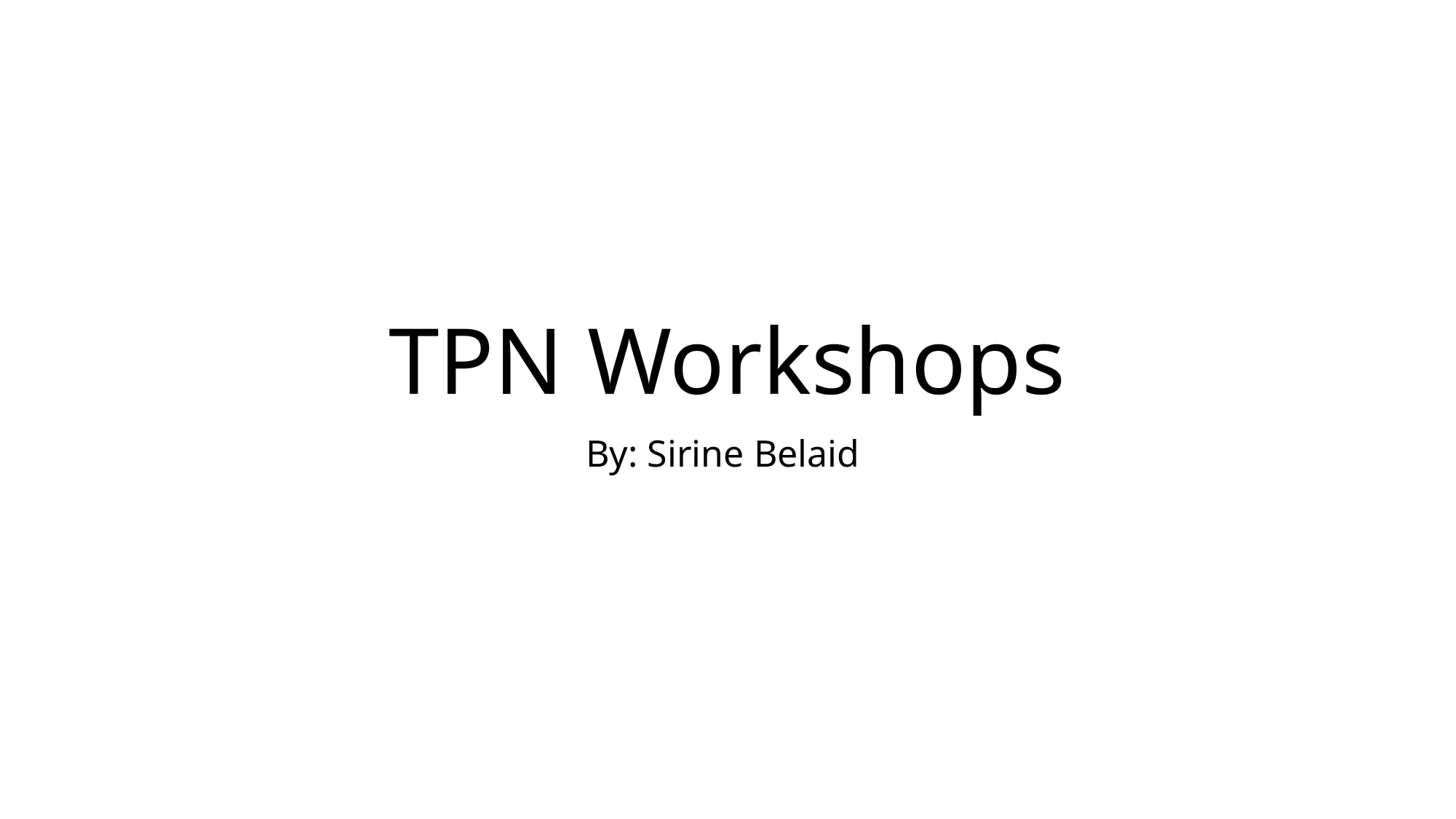

# TPN Workshops
By: Sirine Belaid

## Slide 2
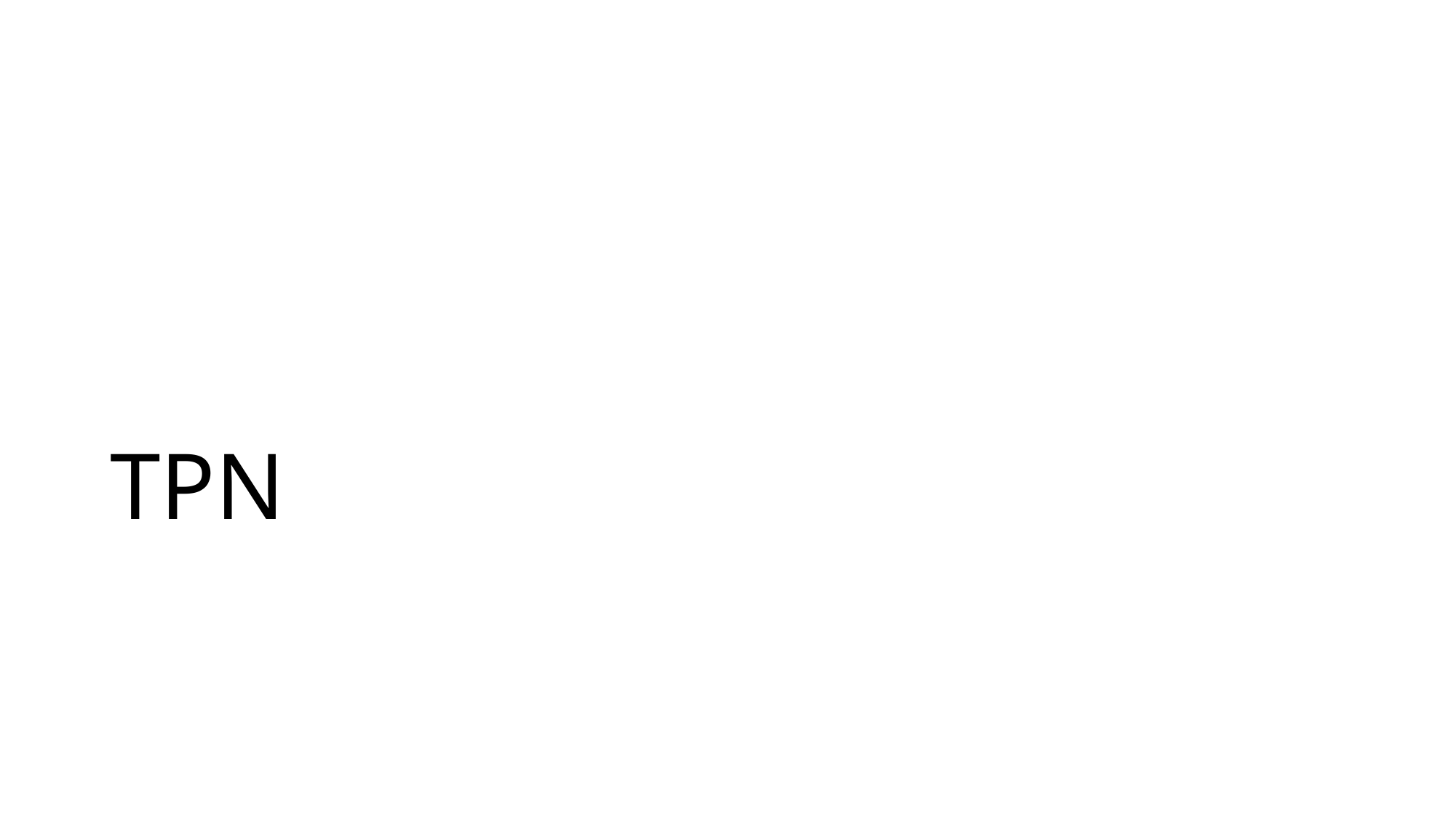

# TPN

## Slide 3
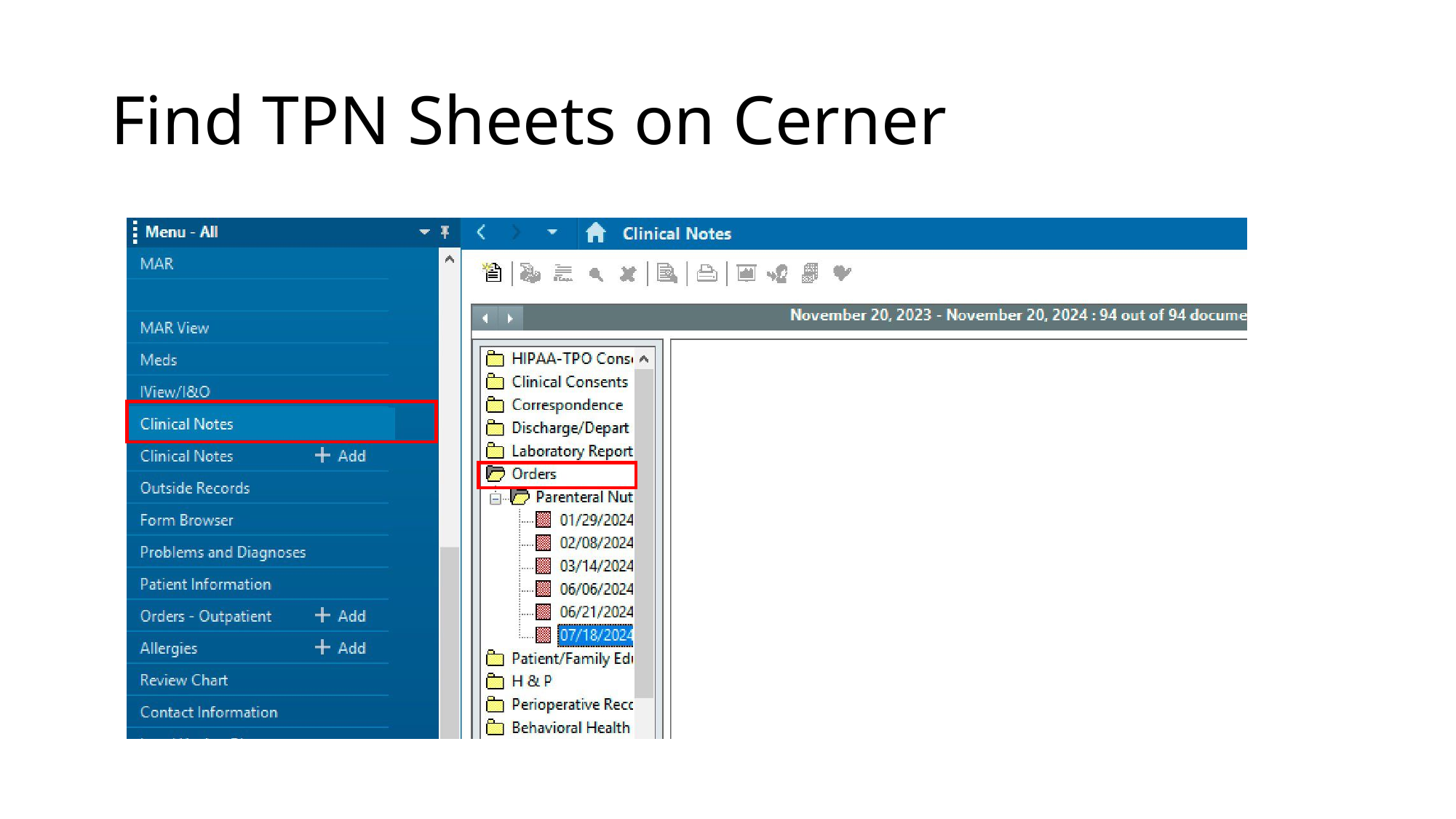

# Find TPN Sheets on Cerner

## Slide 4
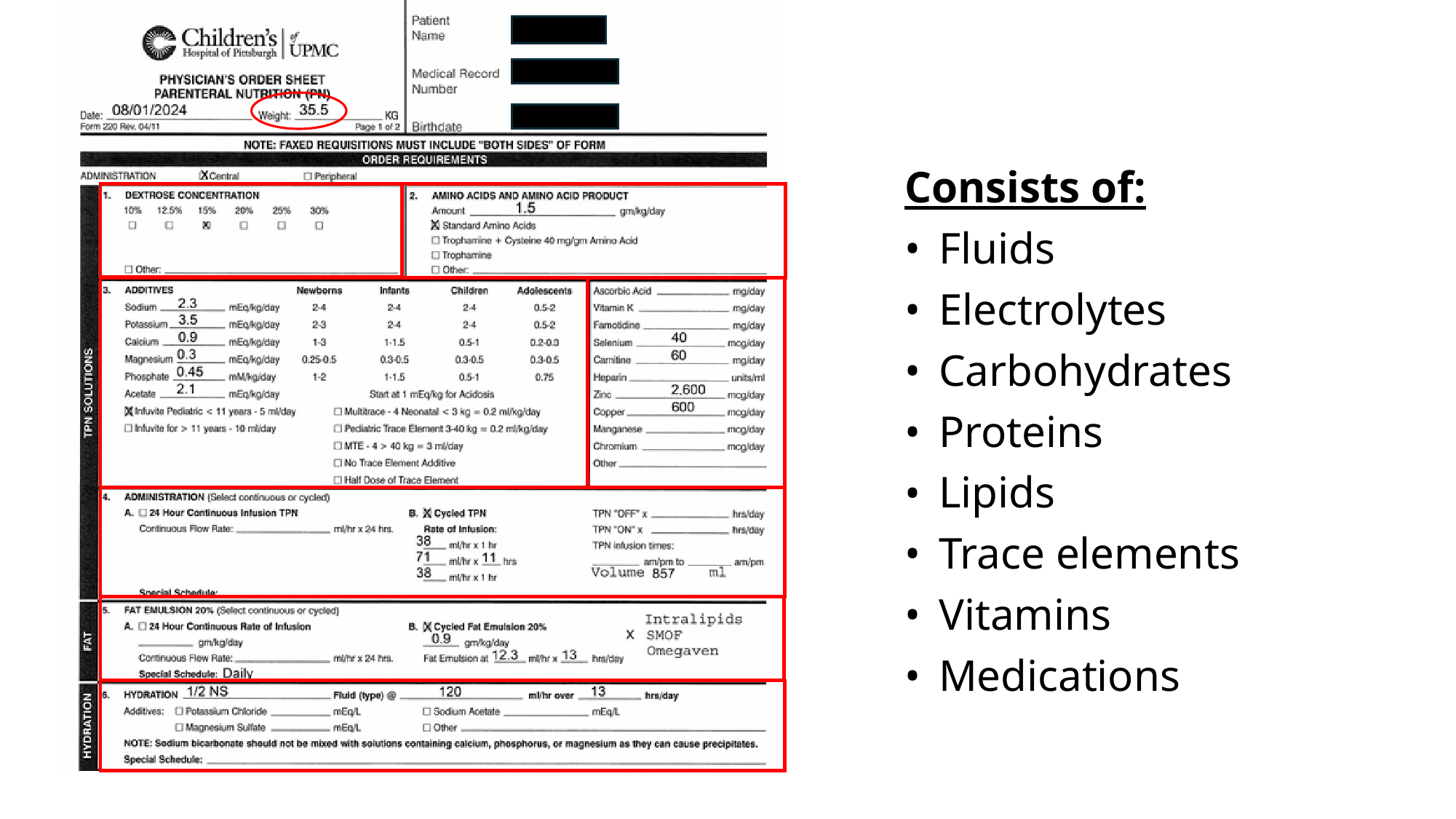

Consists of:
Fluids
Electrolytes
Carbohydrates
Proteins
Lipids
Trace elements
Vitamins
Medications

## Slide 5
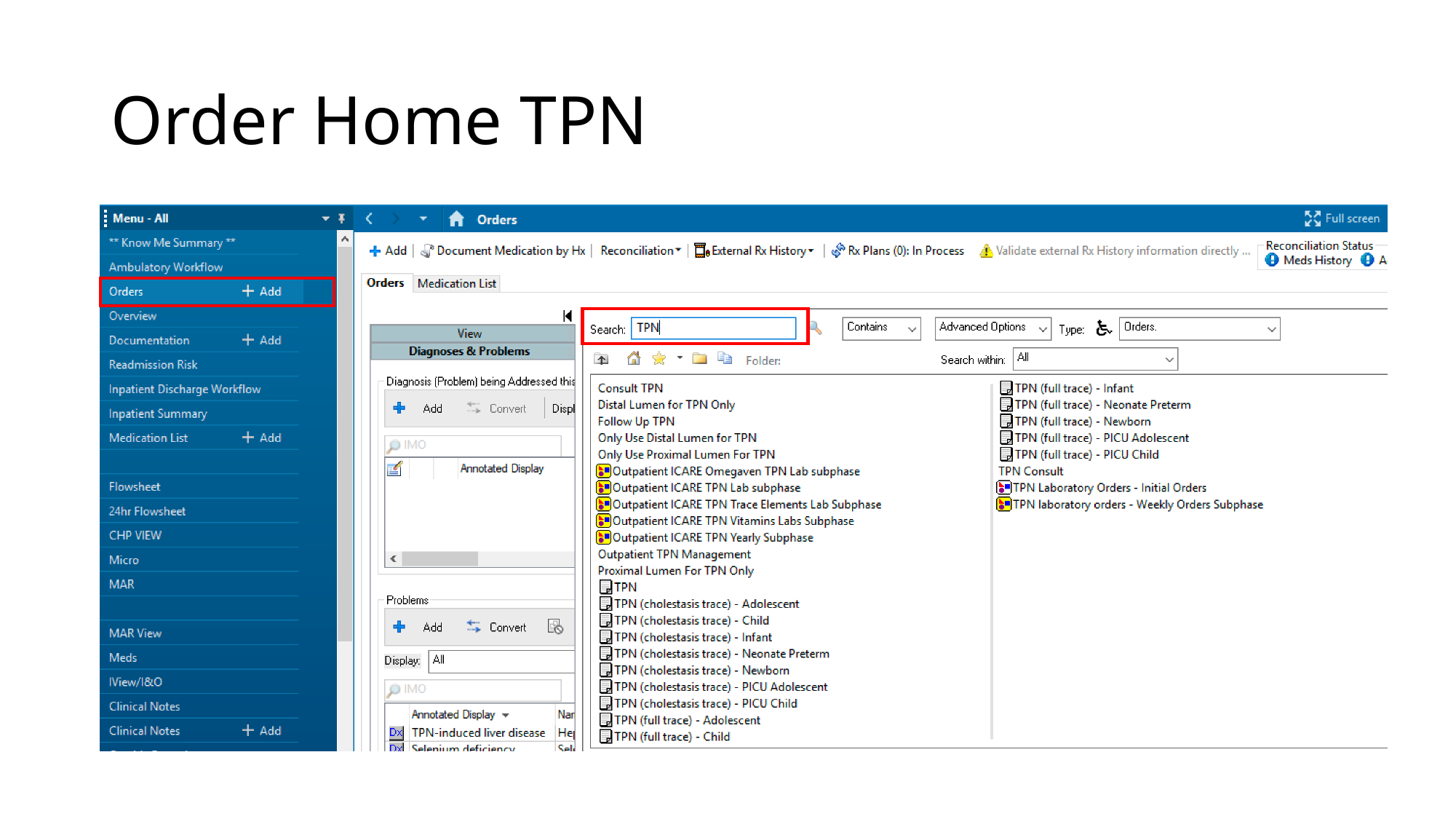

# Order Home TPN

## Slide 6
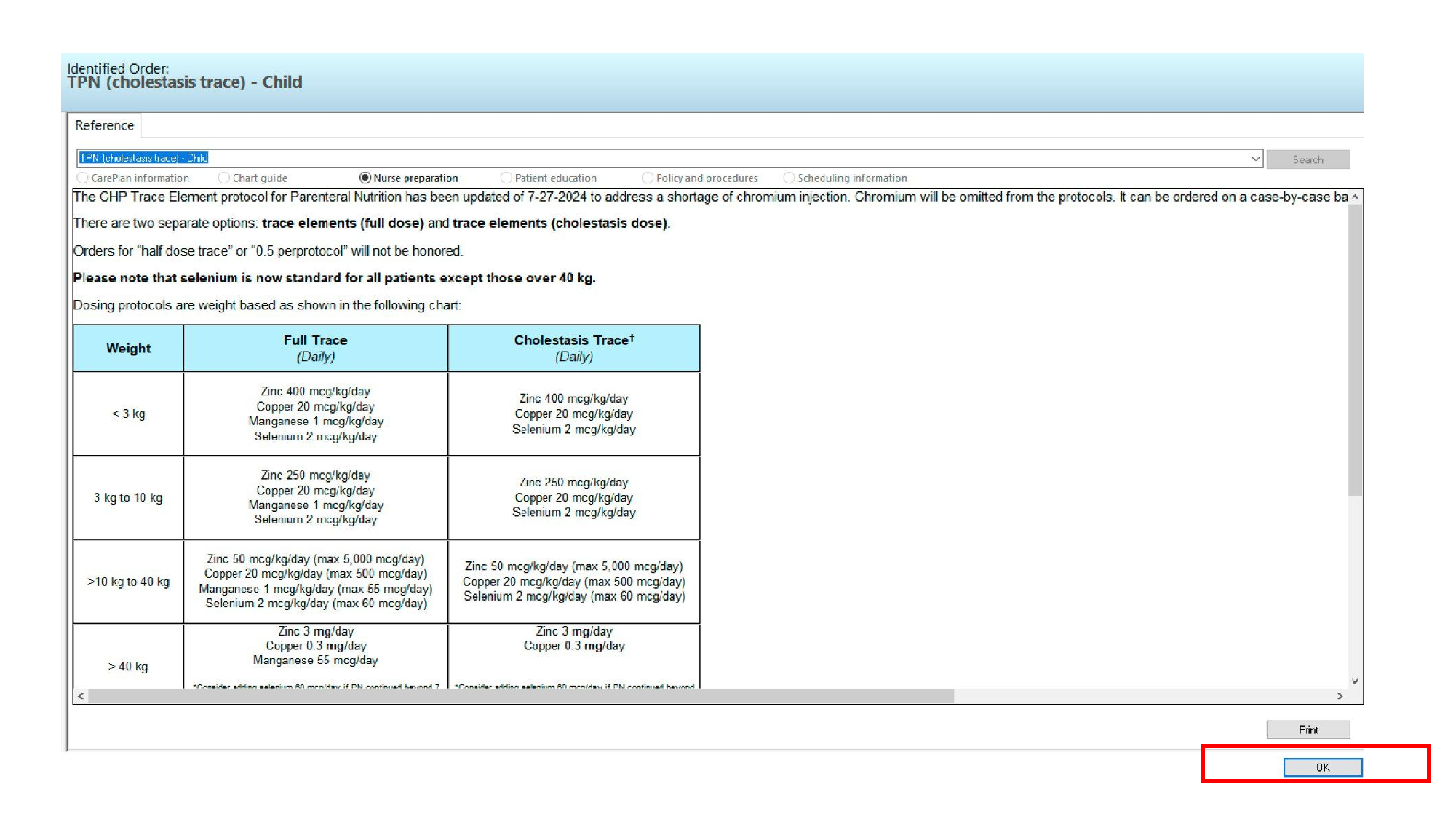

## Slide 7
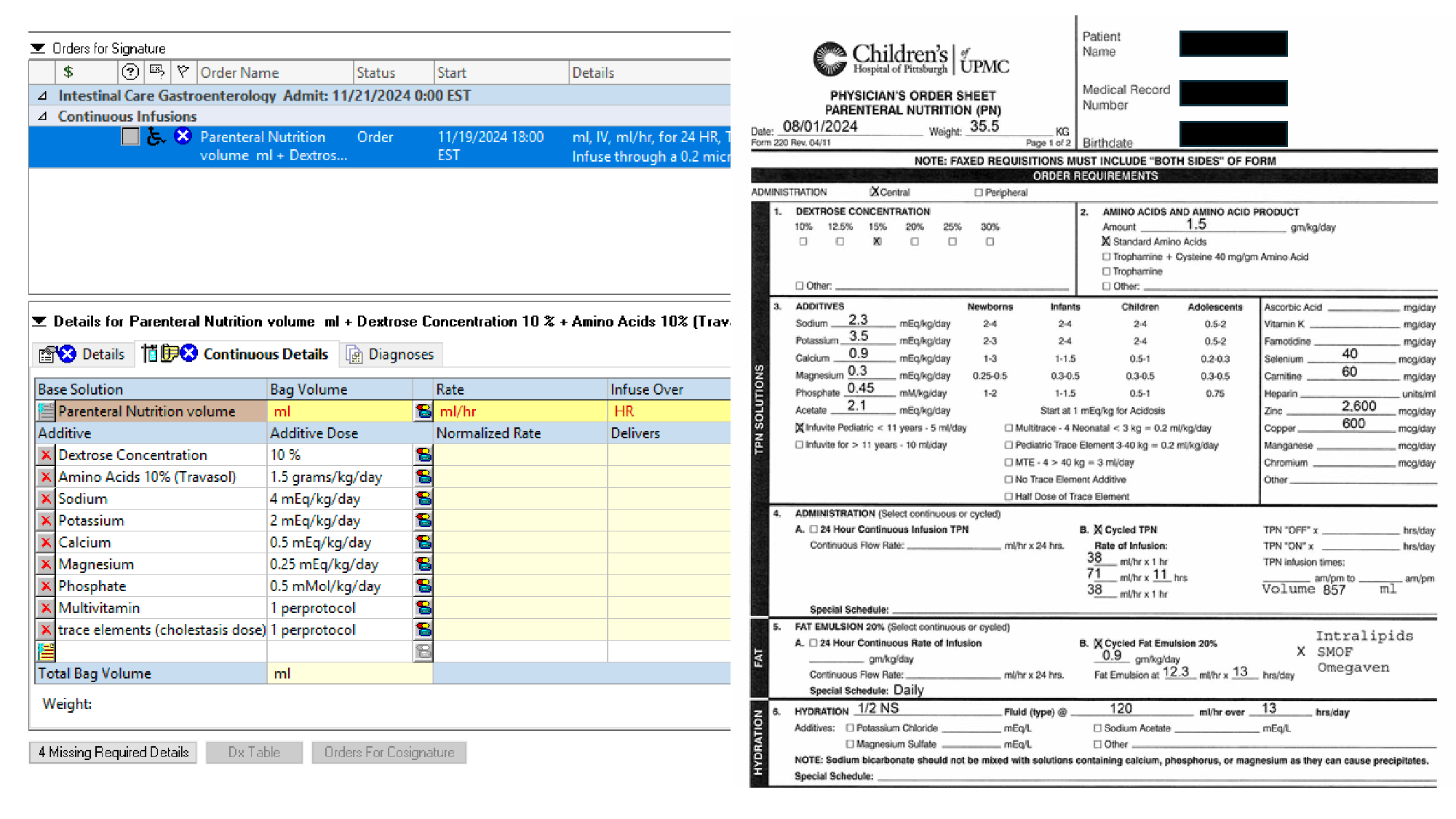

## Slide 8
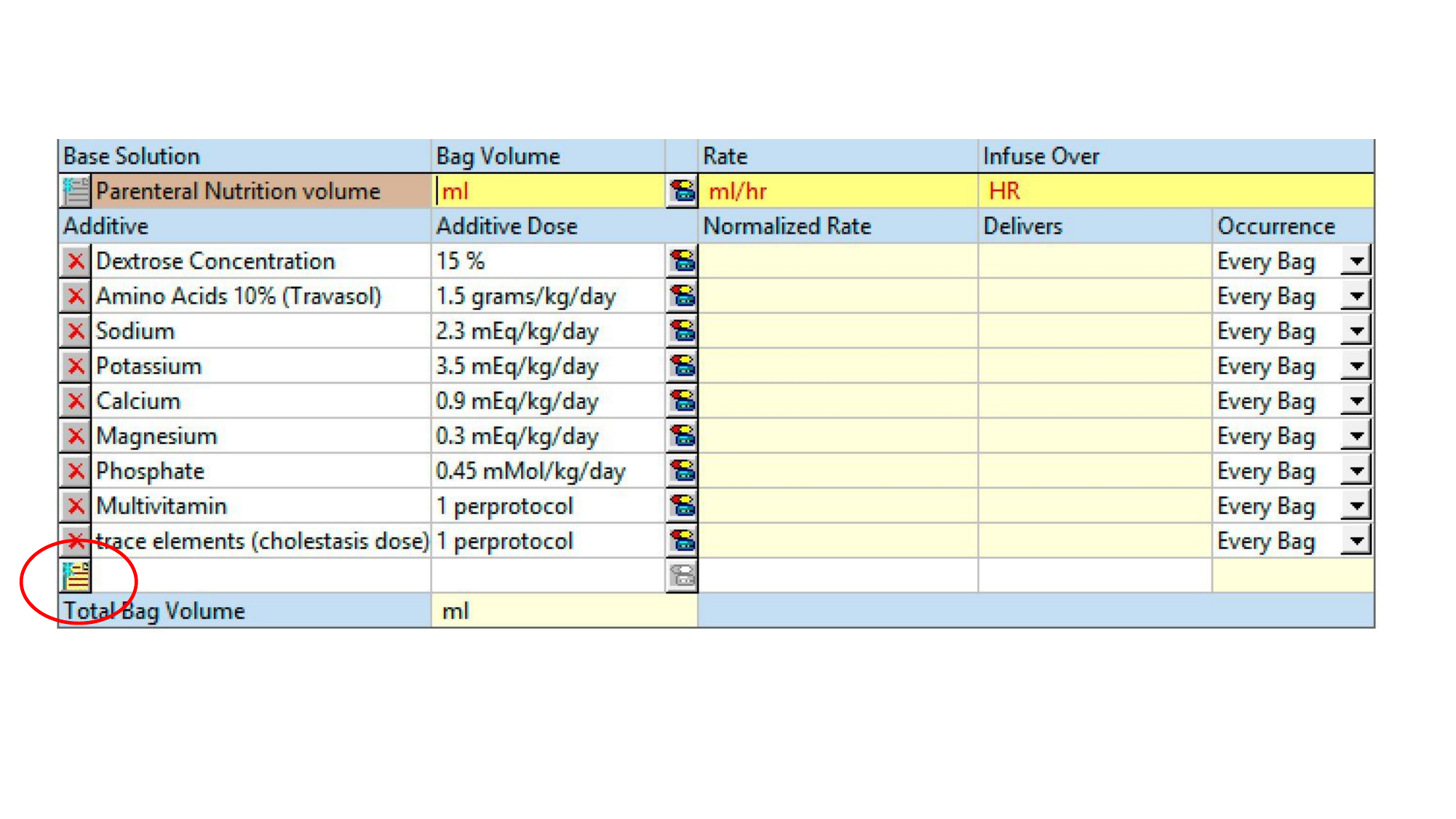

## Slide 9
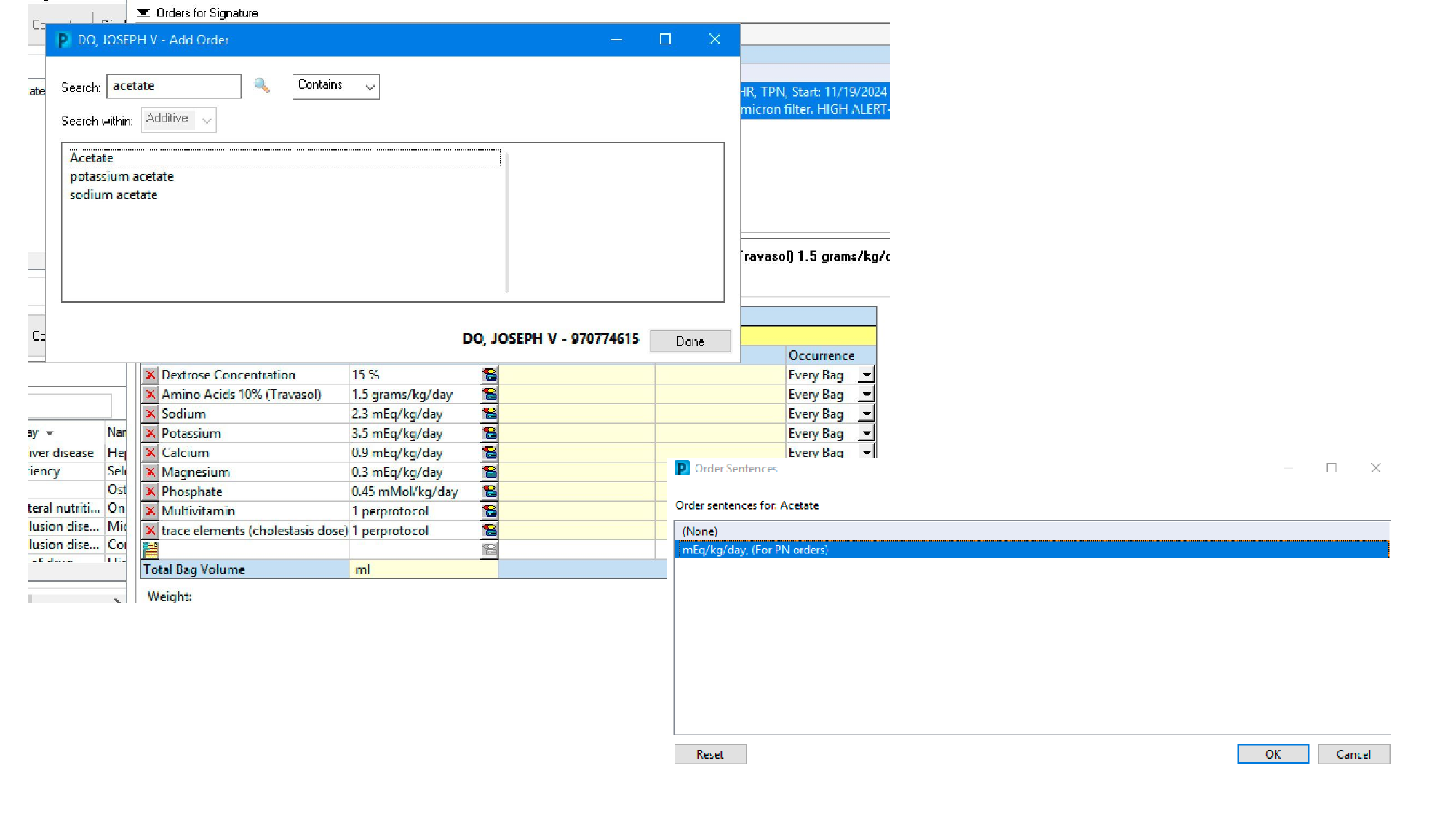

## Slide 10
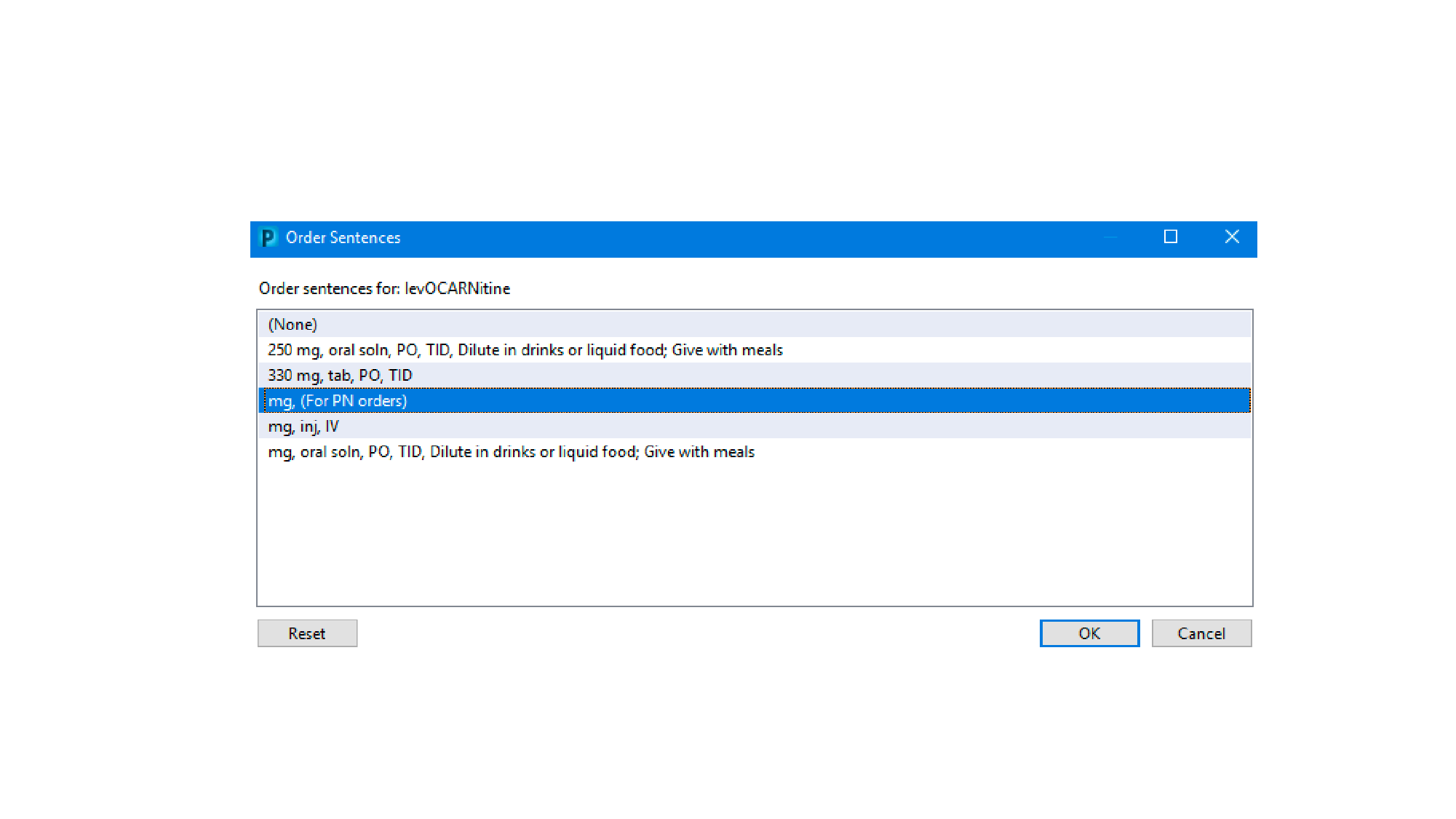

## Slide 11
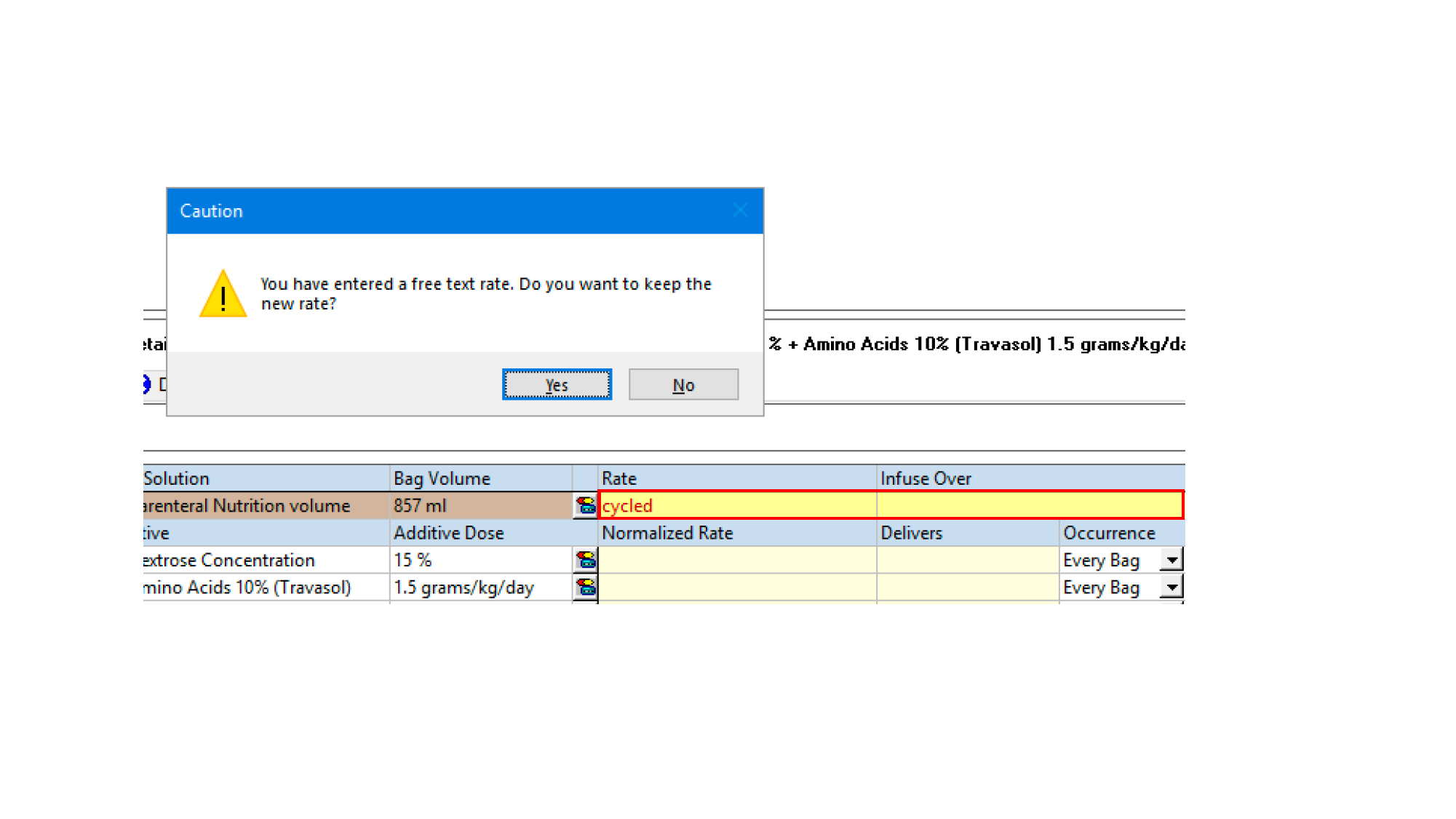

## Slide 12
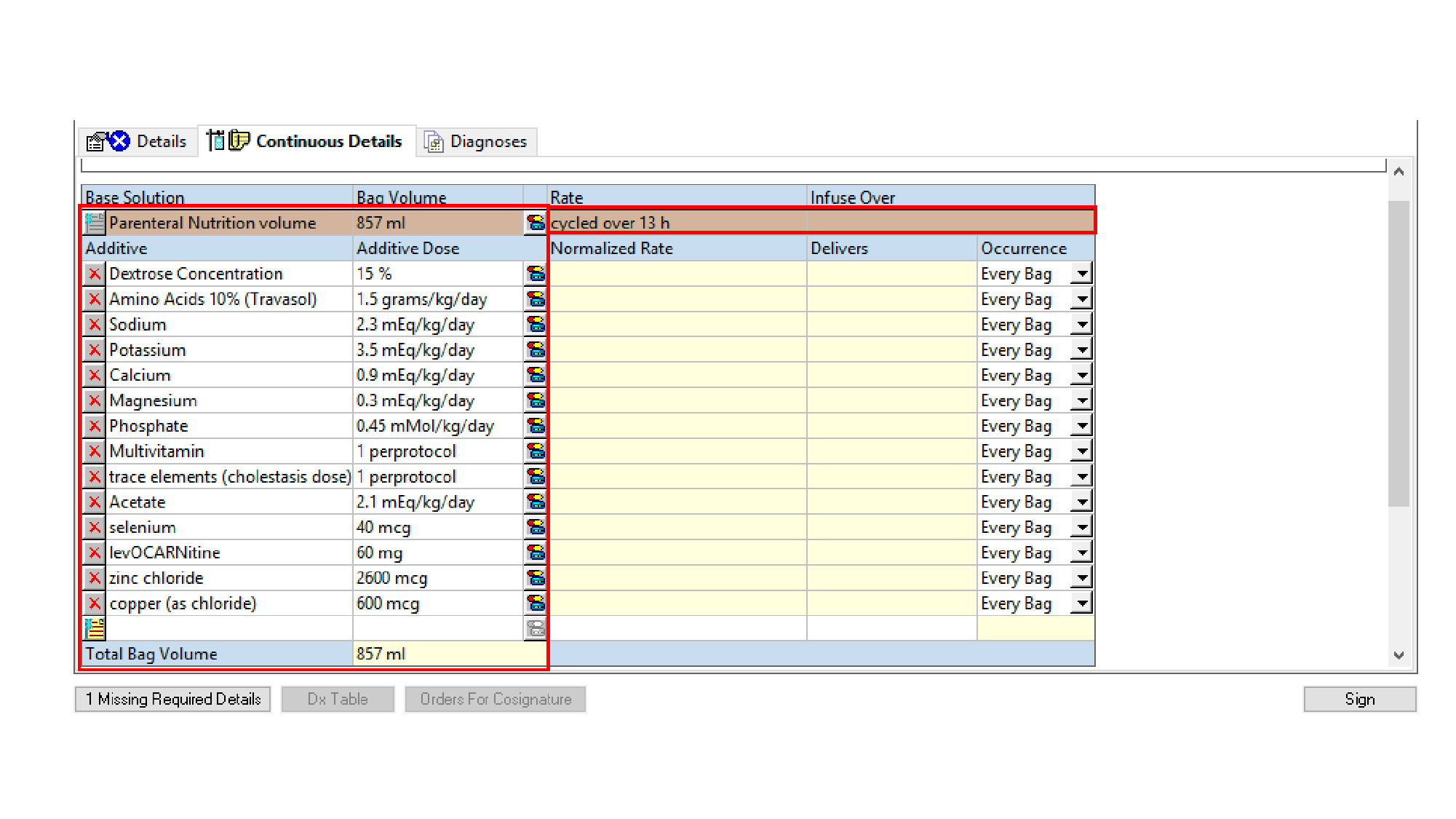

## Slide 13
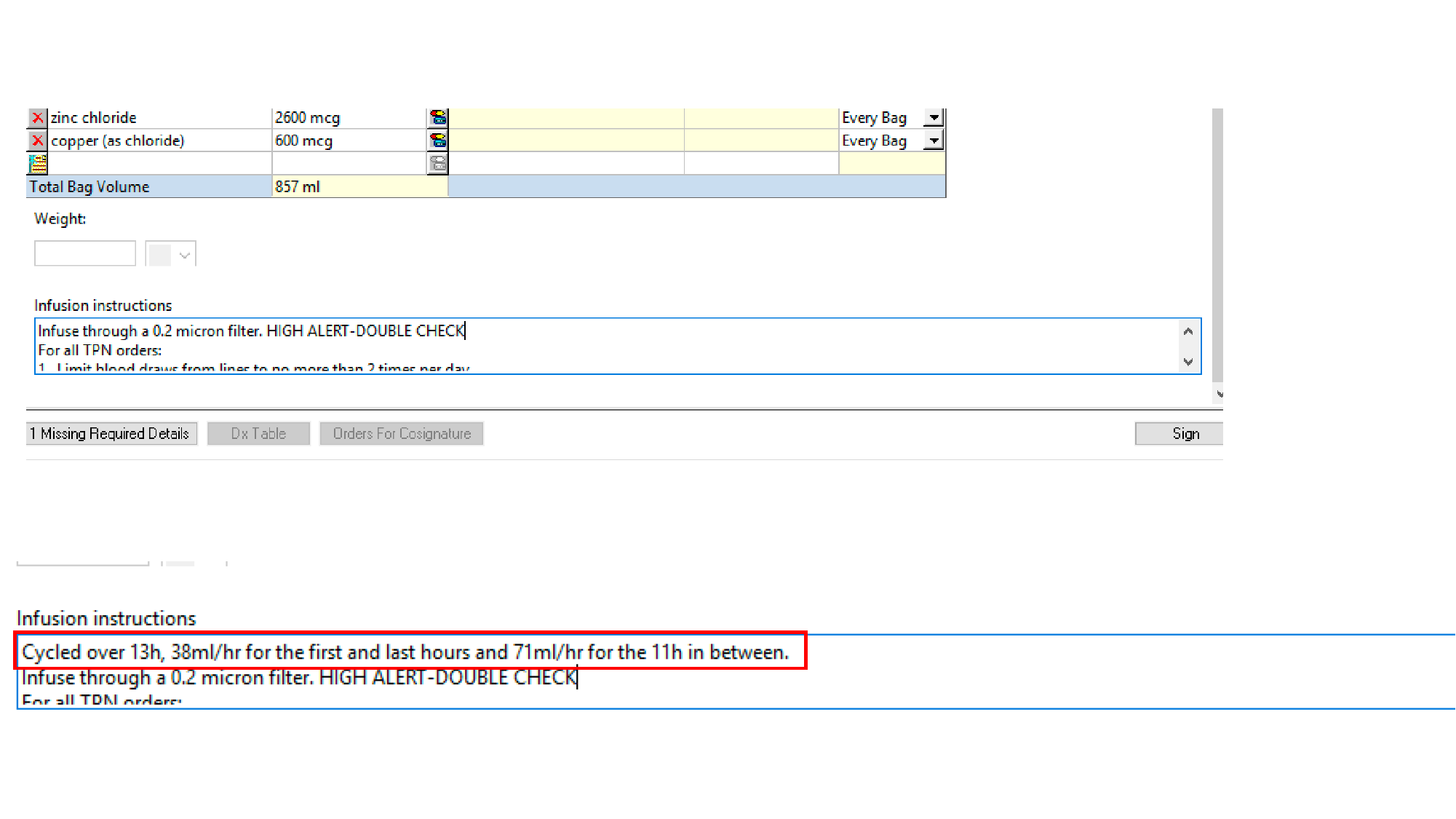

## Slide 14
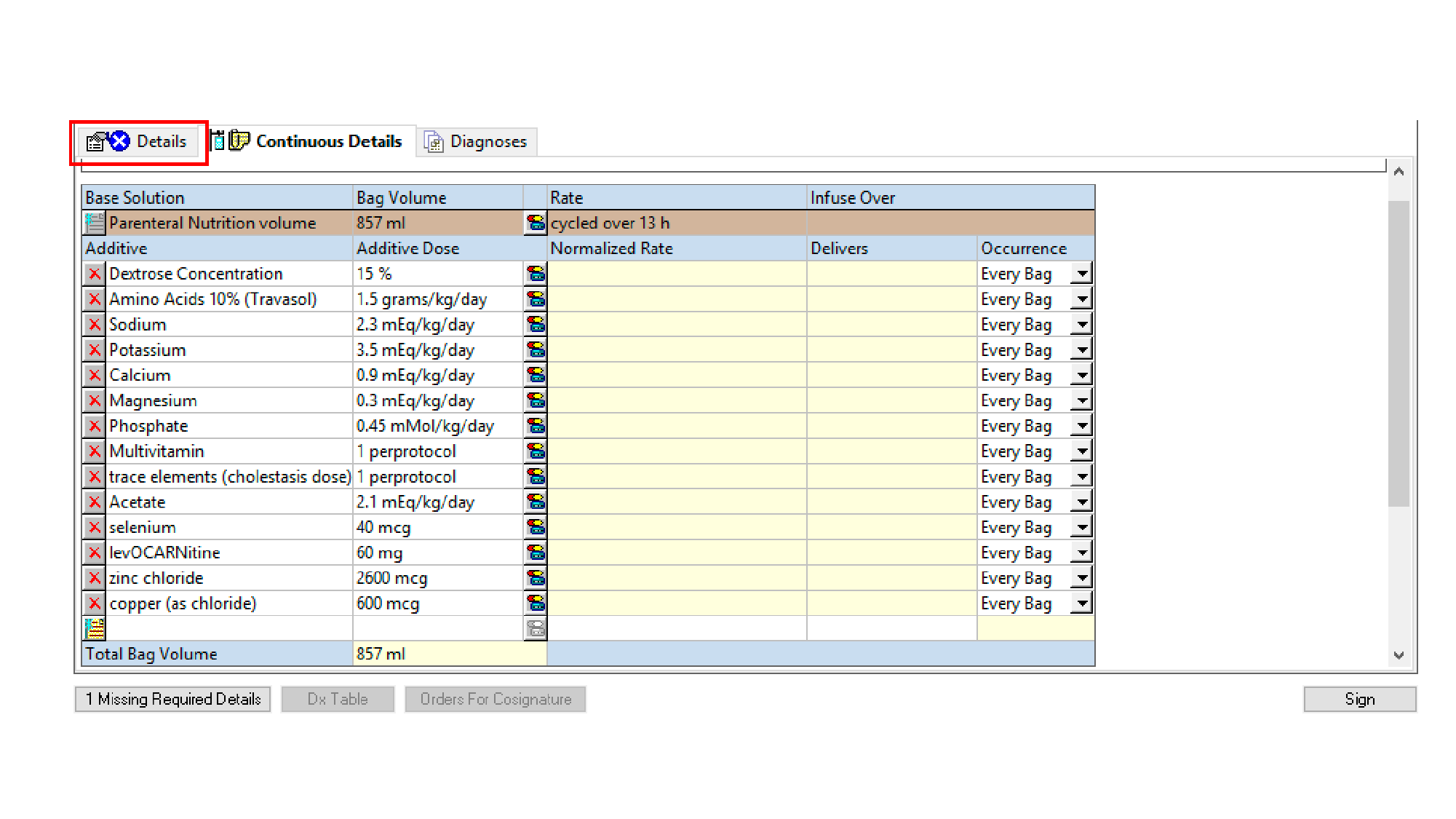

## Slide 15
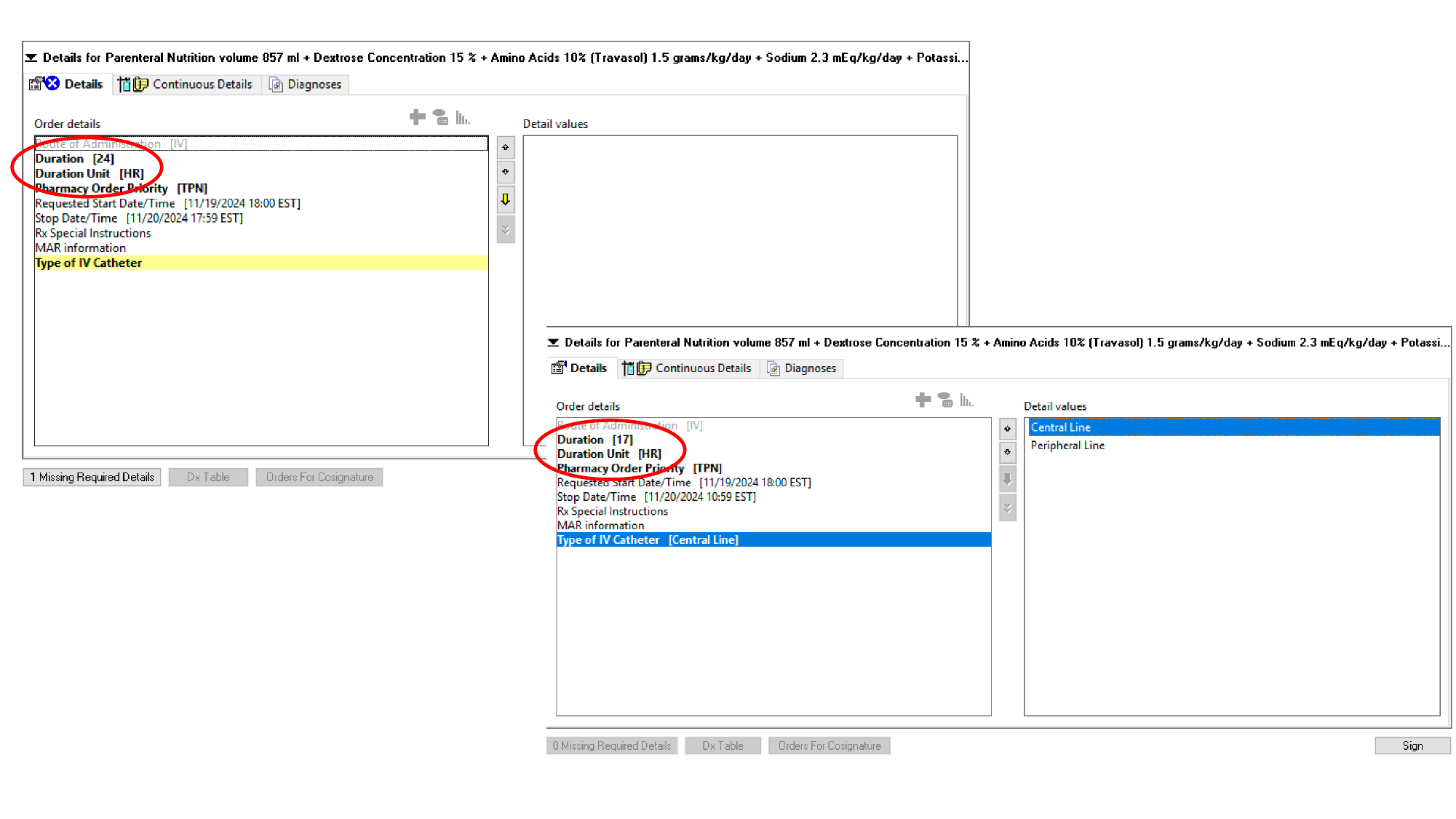

## Slide 16
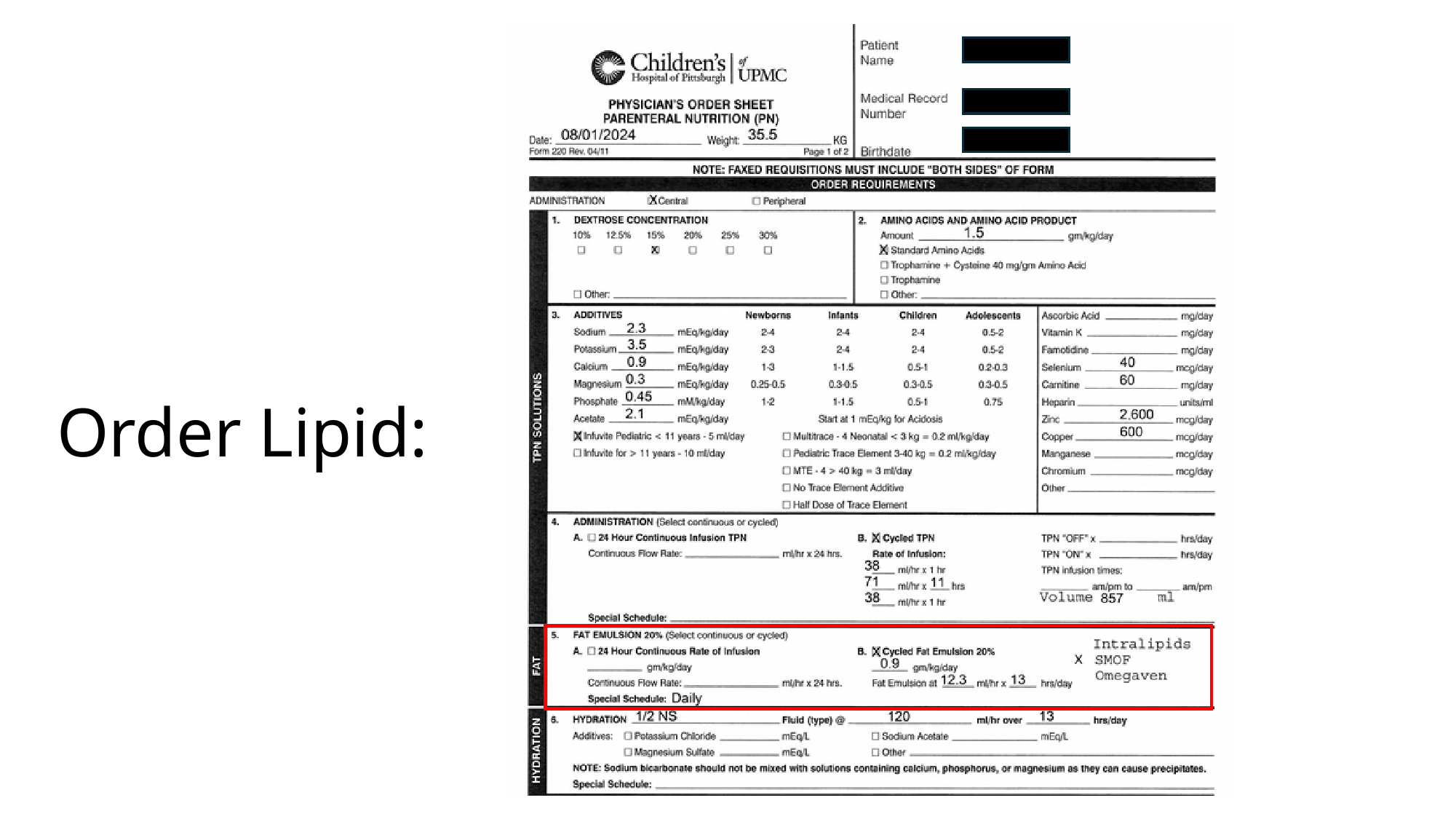

# Order Lipid:

## Slide 17
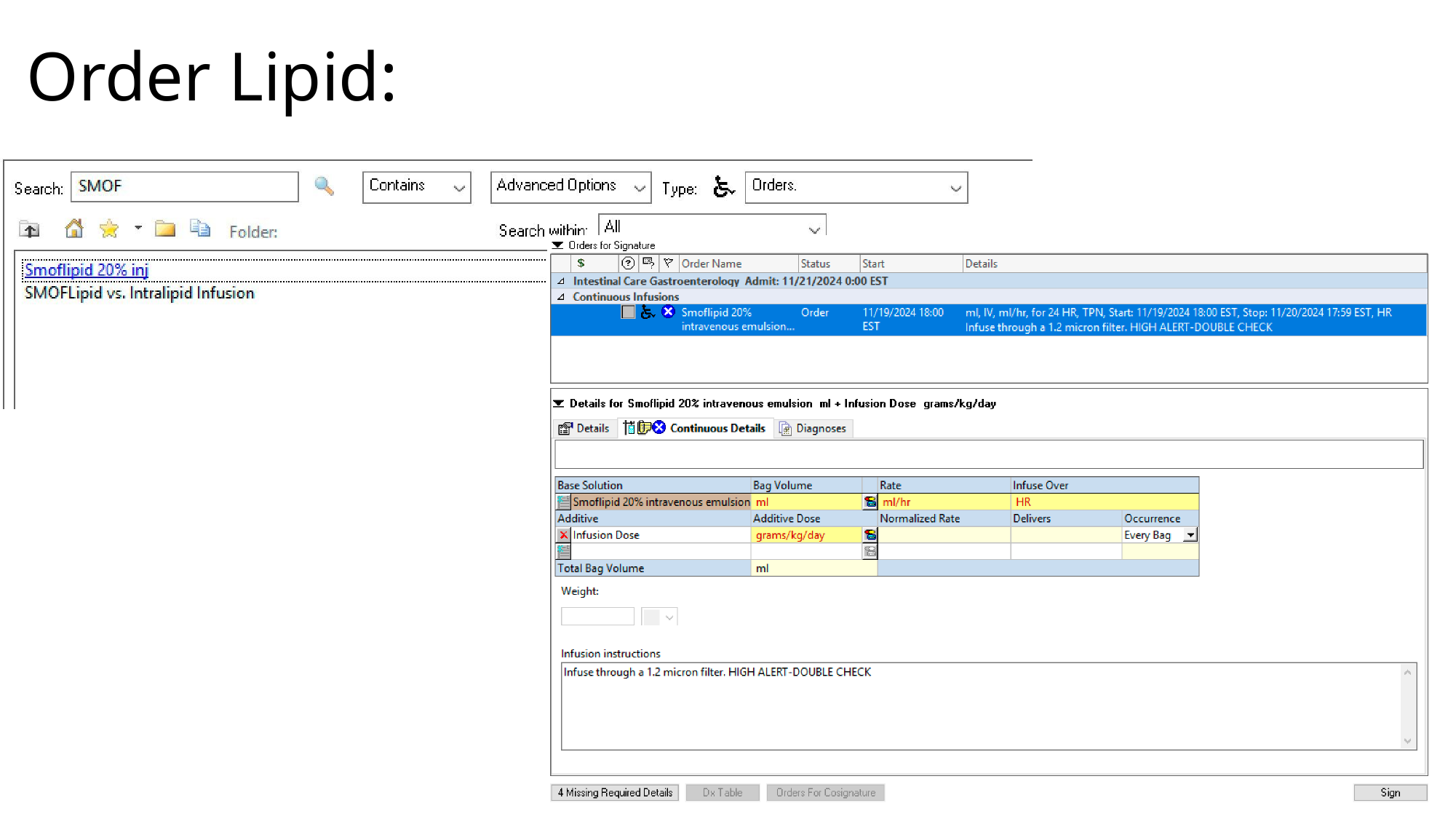

# Order Lipid:

## Slide 18
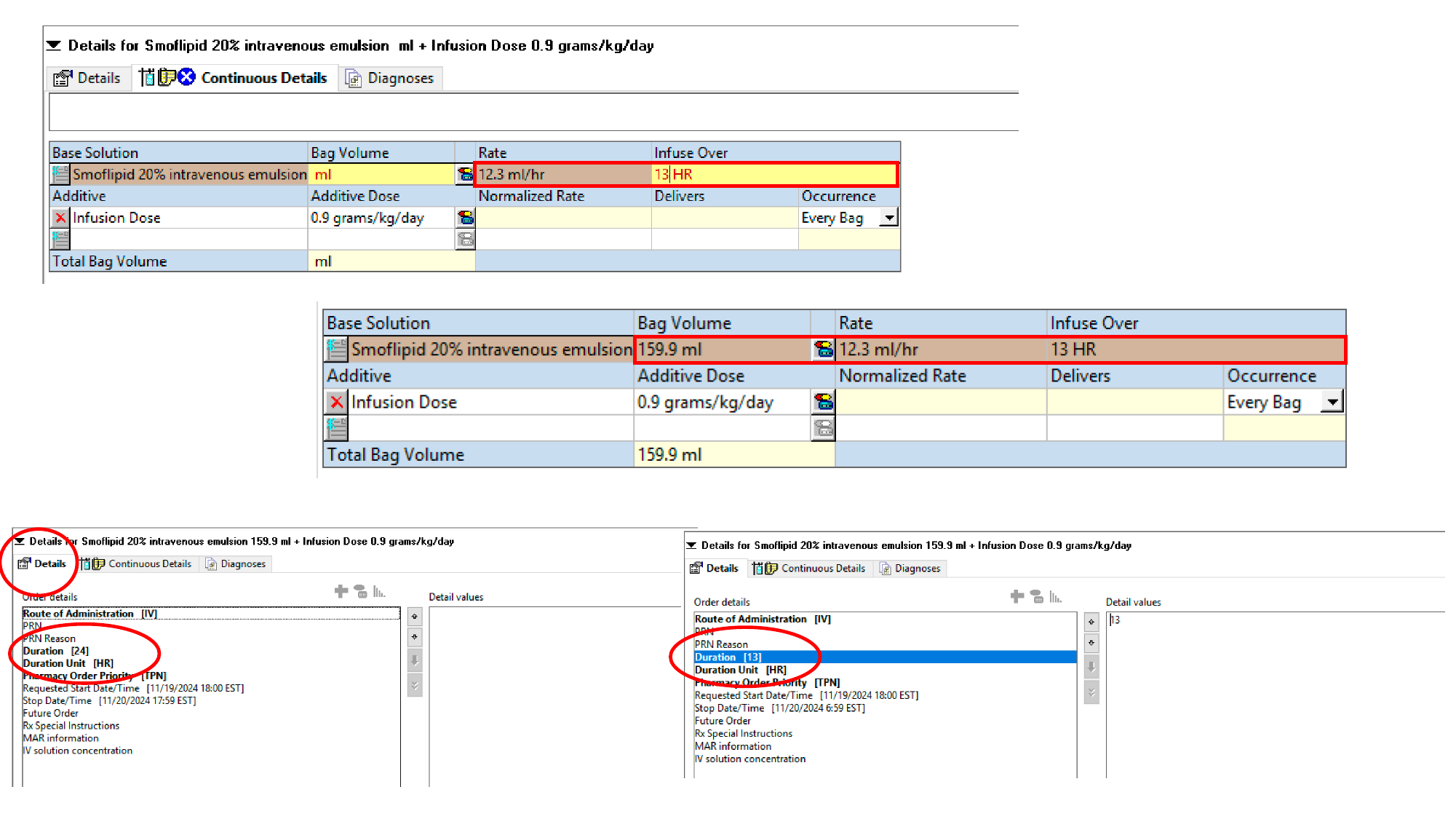

## Slide 19
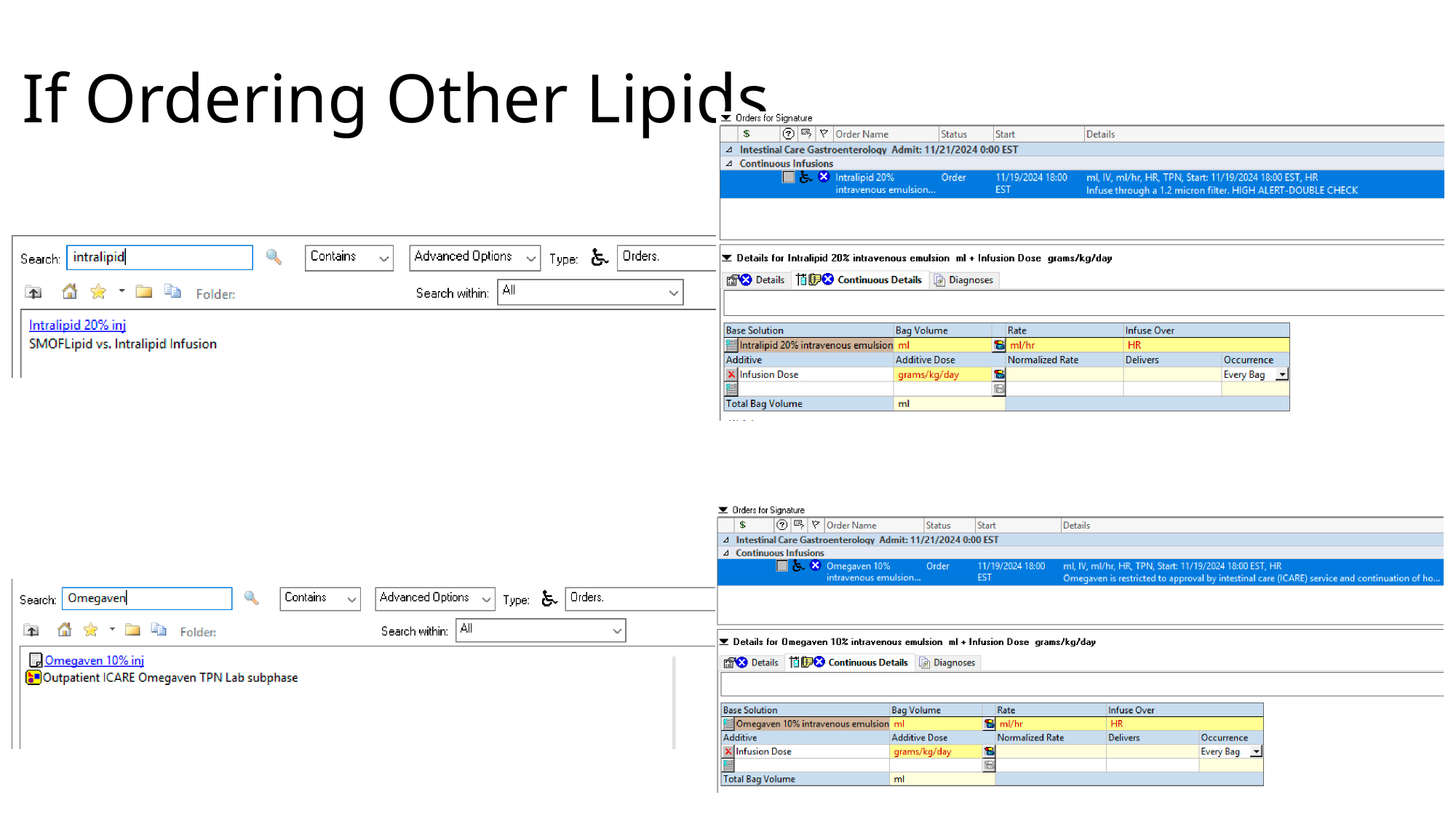

# If Ordering Other Lipids

## Slide 20
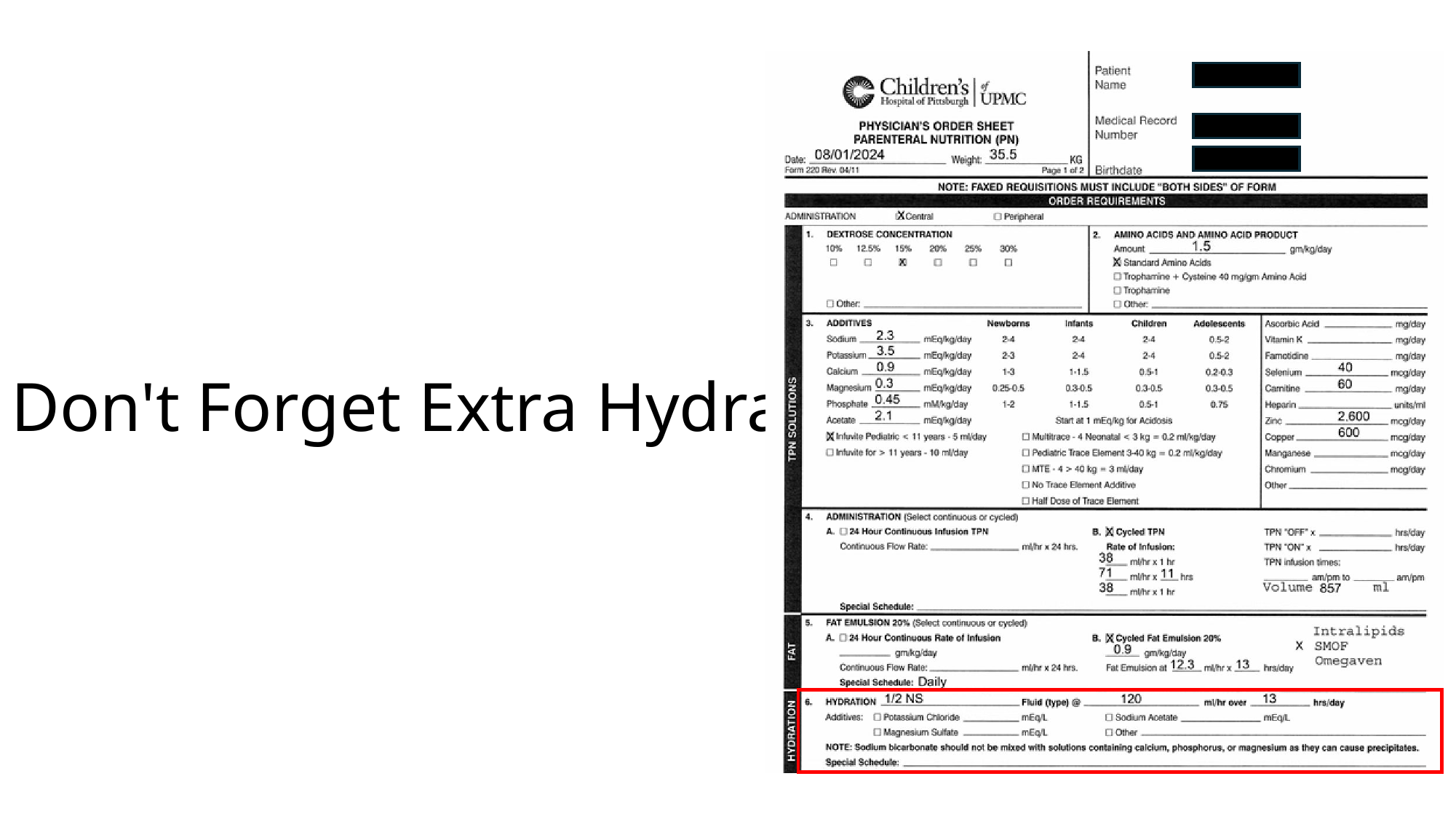

# Don't Forget Extra Hydration

## Slide 21
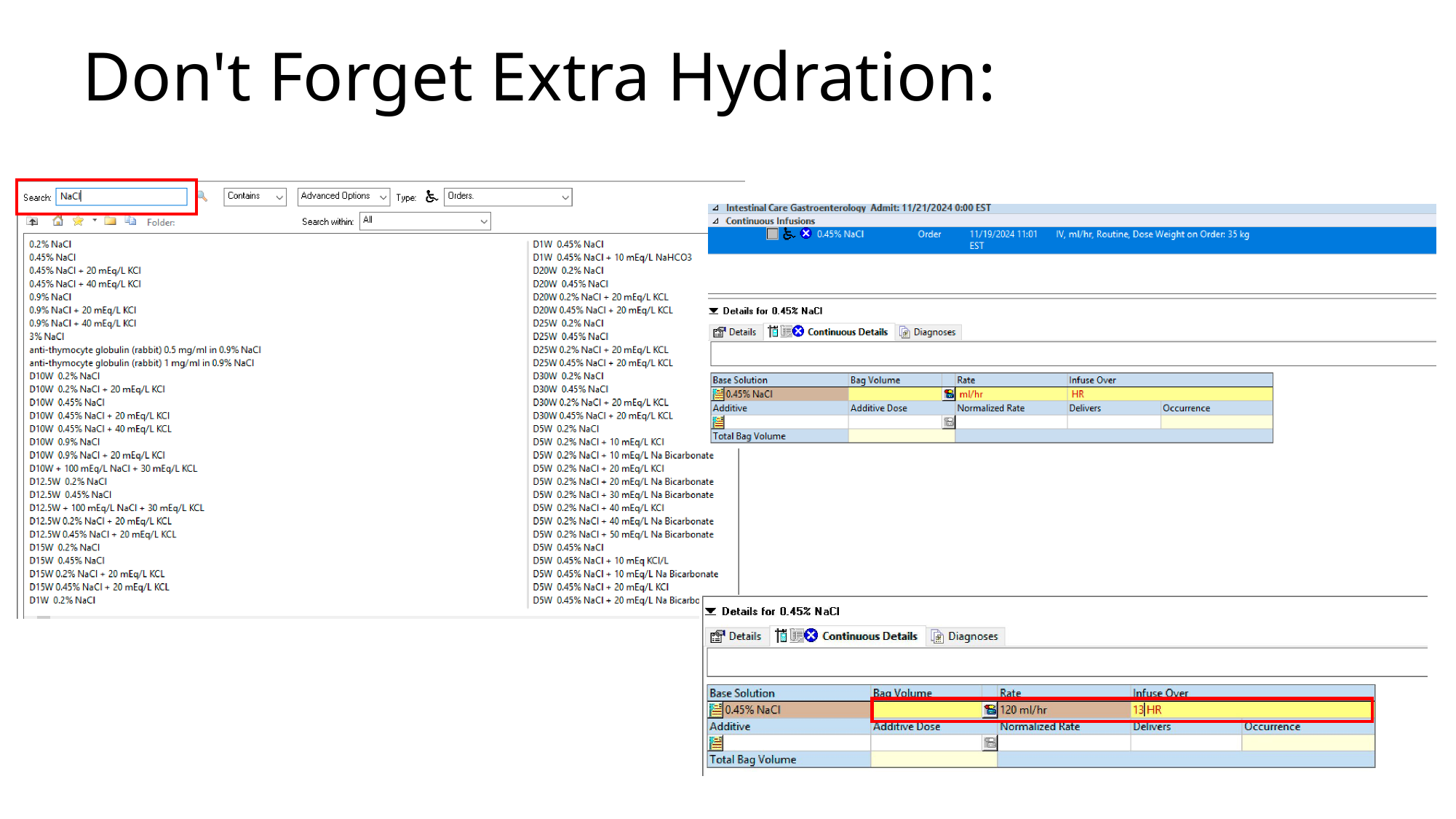

# Don't Forget Extra Hydration:

## Slide 22
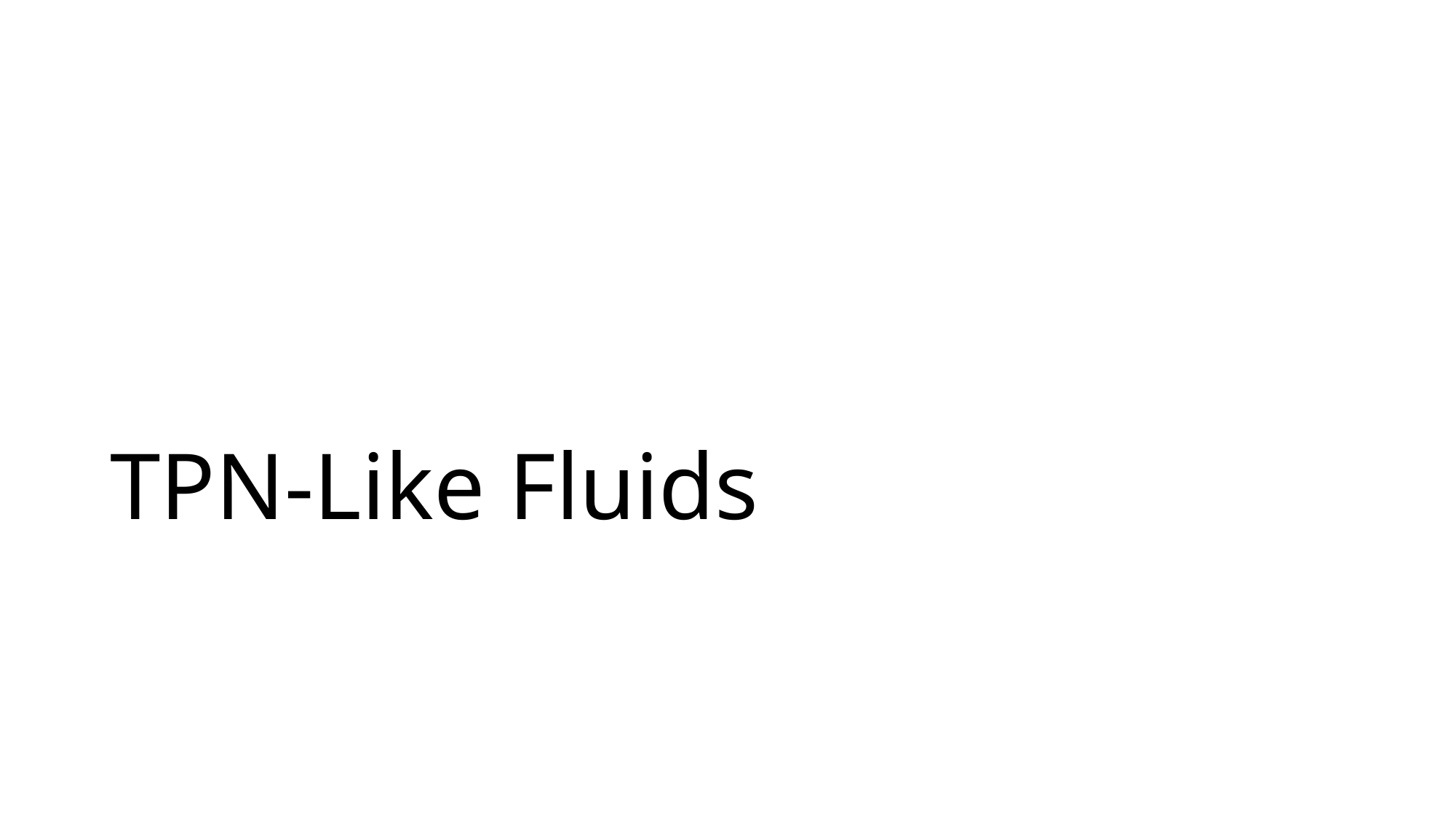

# TPN-Like Fluids

## Slide 23
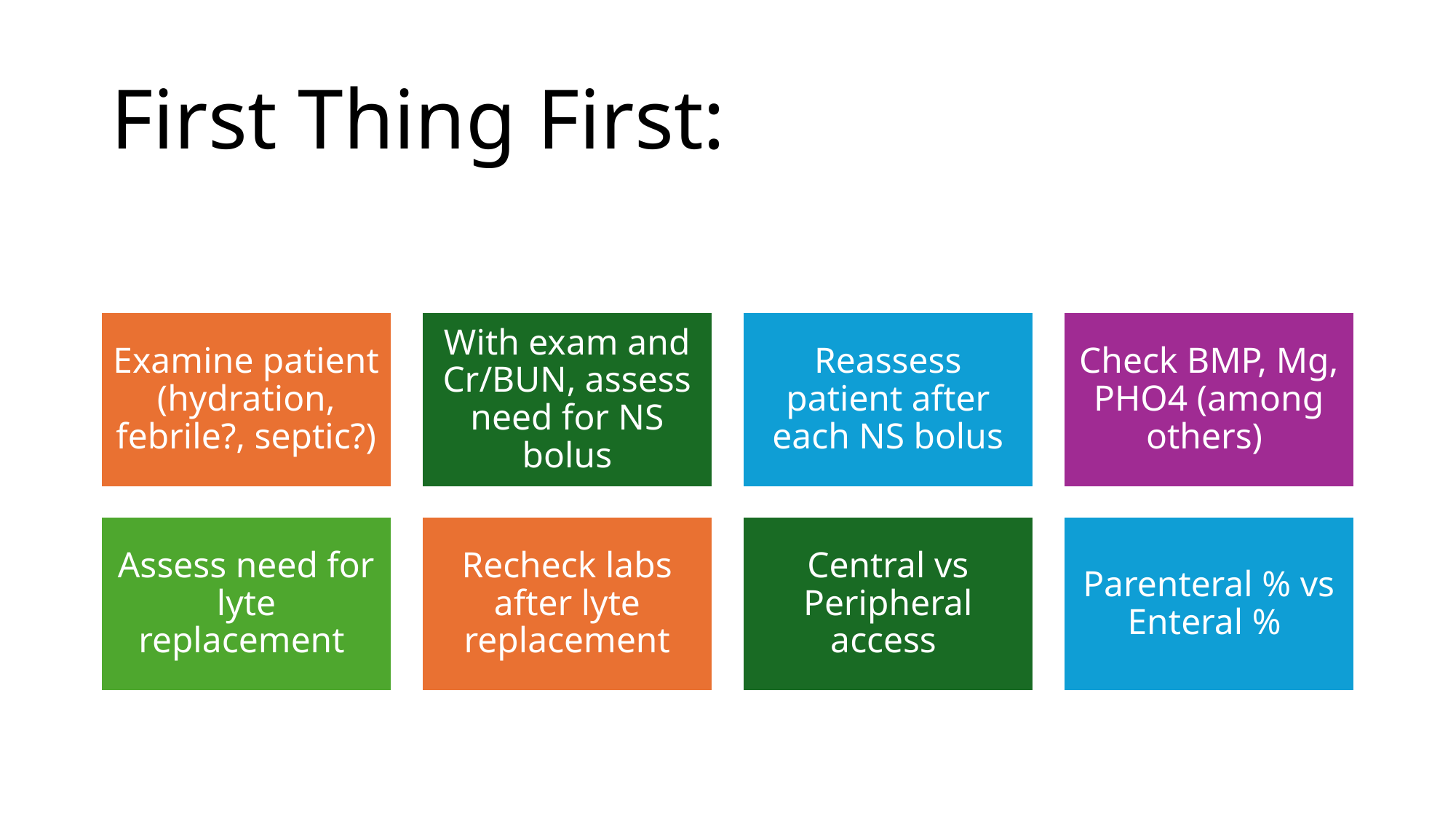

# First Thing First:

## Slide 24
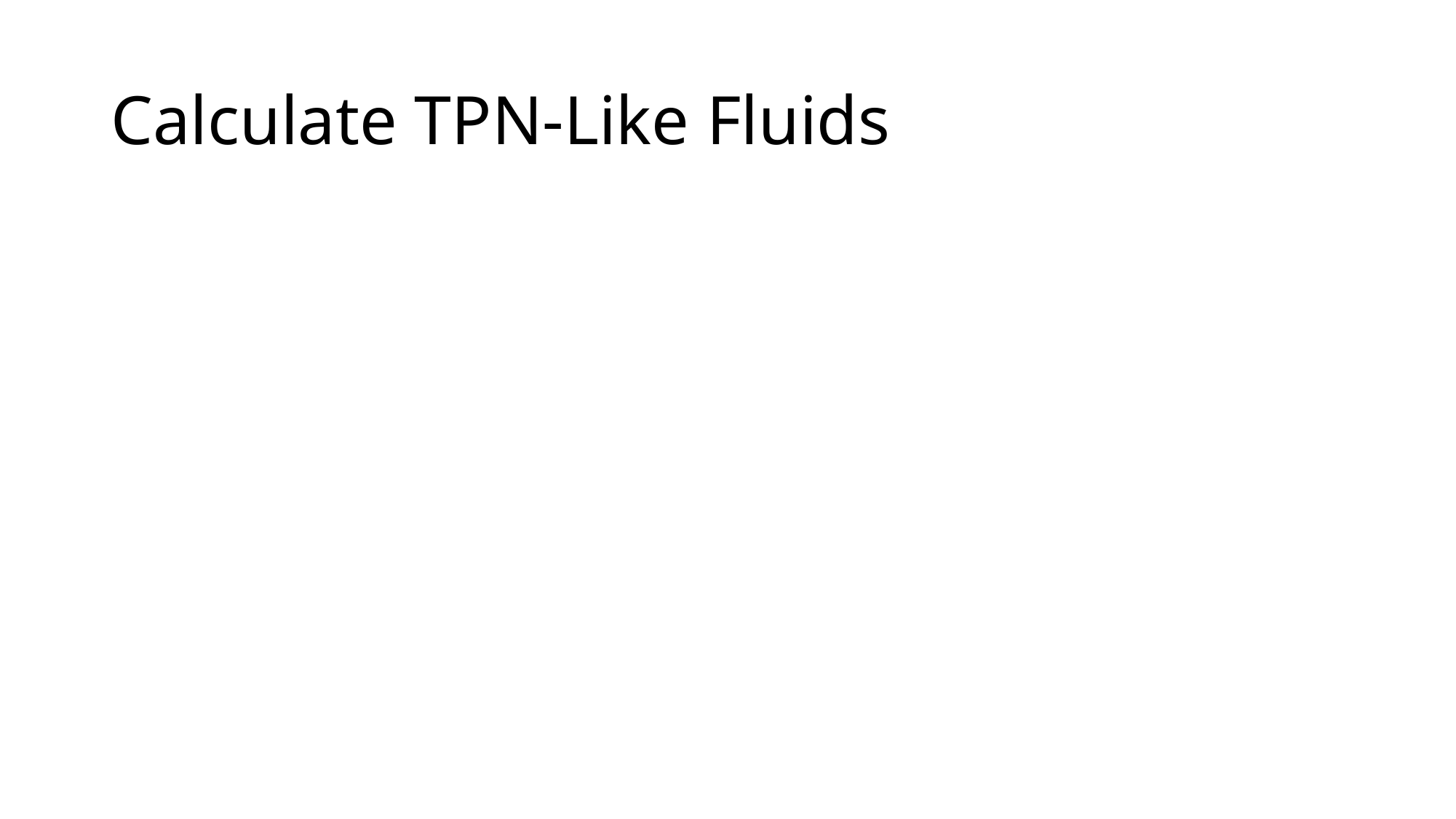

# Calculate TPN-Like Fluids

## Slide 25
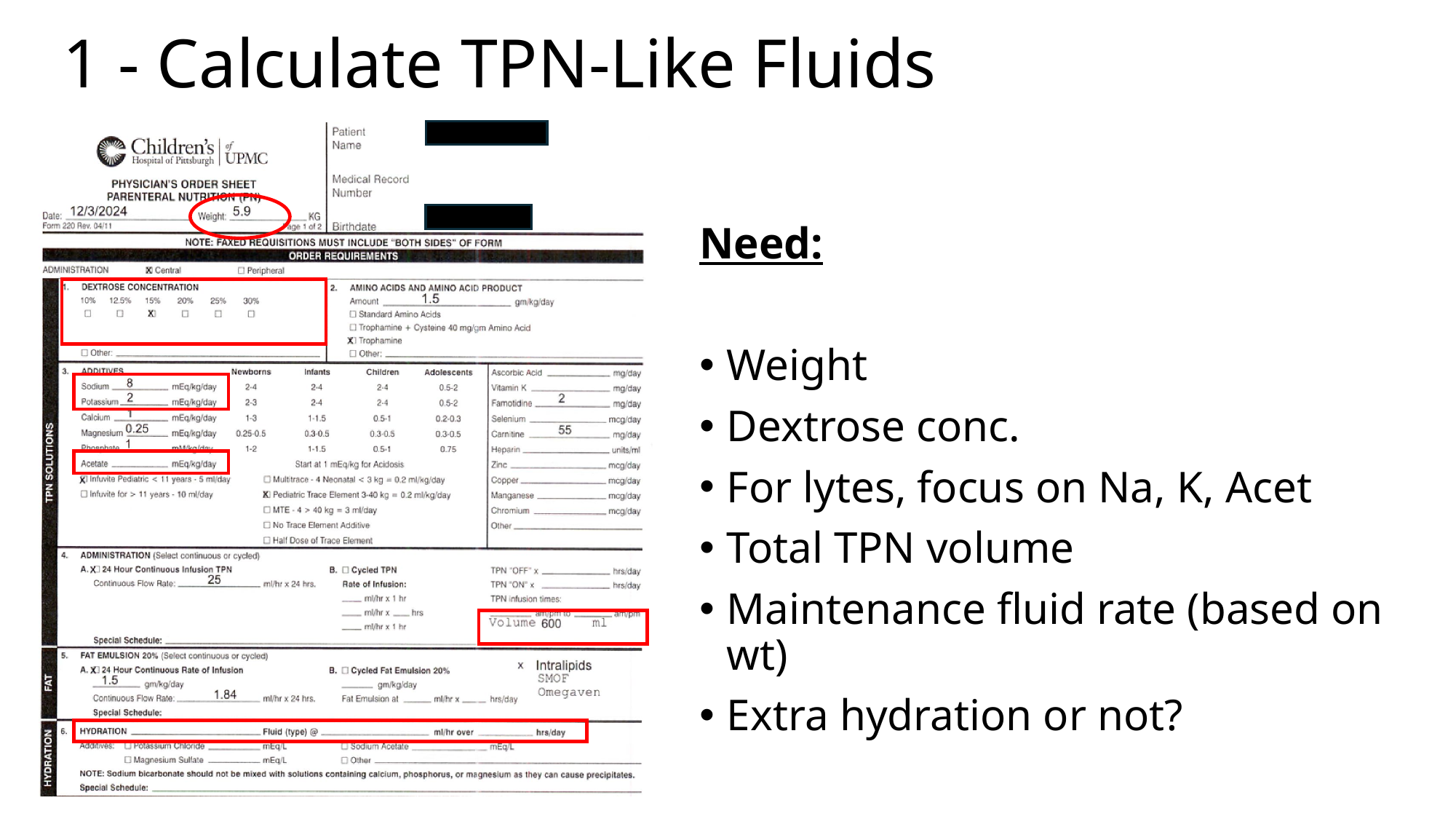

# 1 - Calculate TPN-Like Fluids
Need:
Weight
Dextrose conc.
For lytes, focus on Na, K, Acet
Total TPN volume
Maintenance fluid rate (based on wt)
Extra hydration or not?

## Slide 26
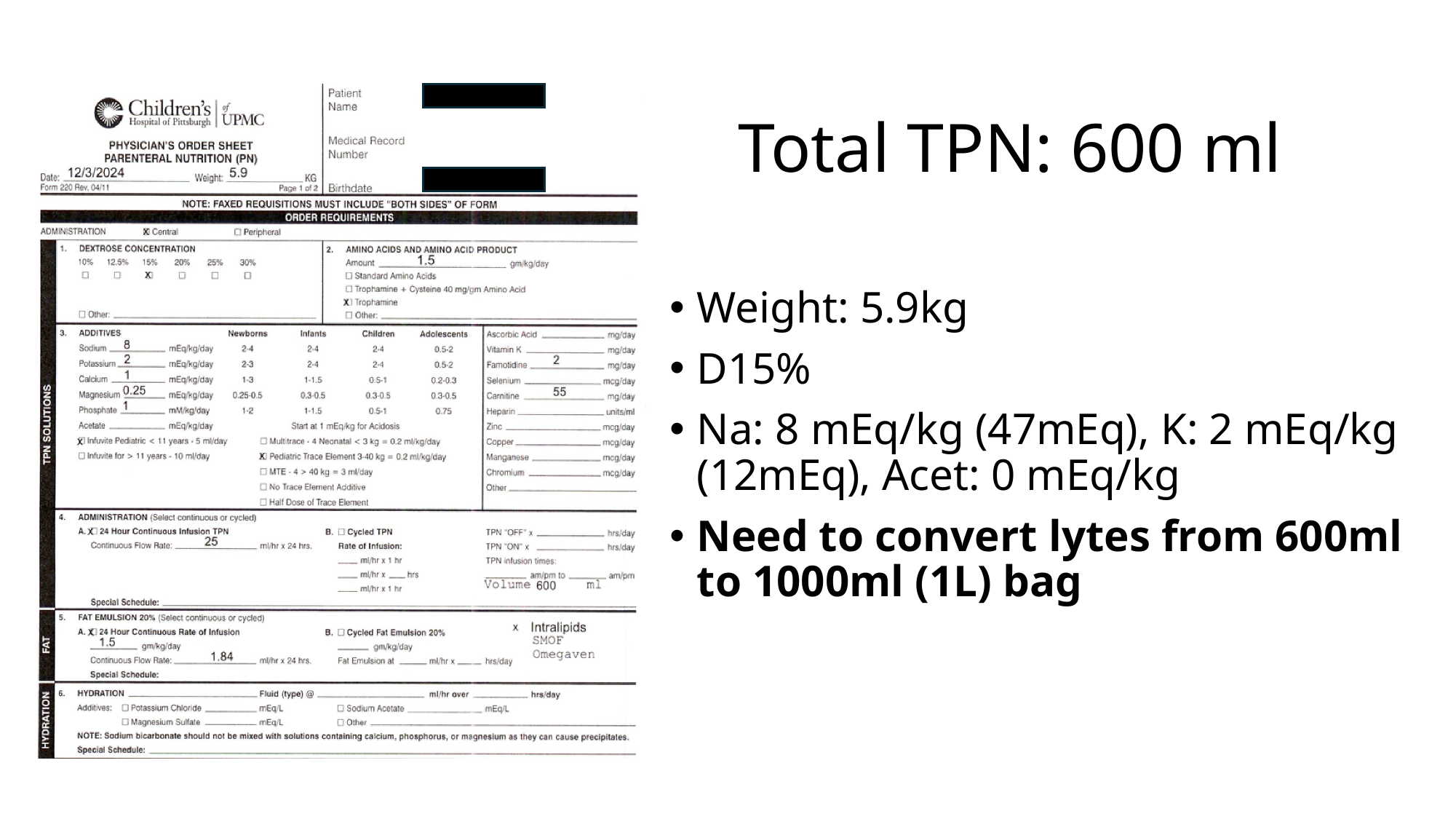

# Total TPN: 600 ml
Weight: 5.9kg
D15%
Na: 8 mEq/kg (47mEq), K: 2 mEq/kg (12mEq), Acet: 0 mEq/kg
Need to convert lytes from 600ml to 1000ml (1L) bag

## Slide 27
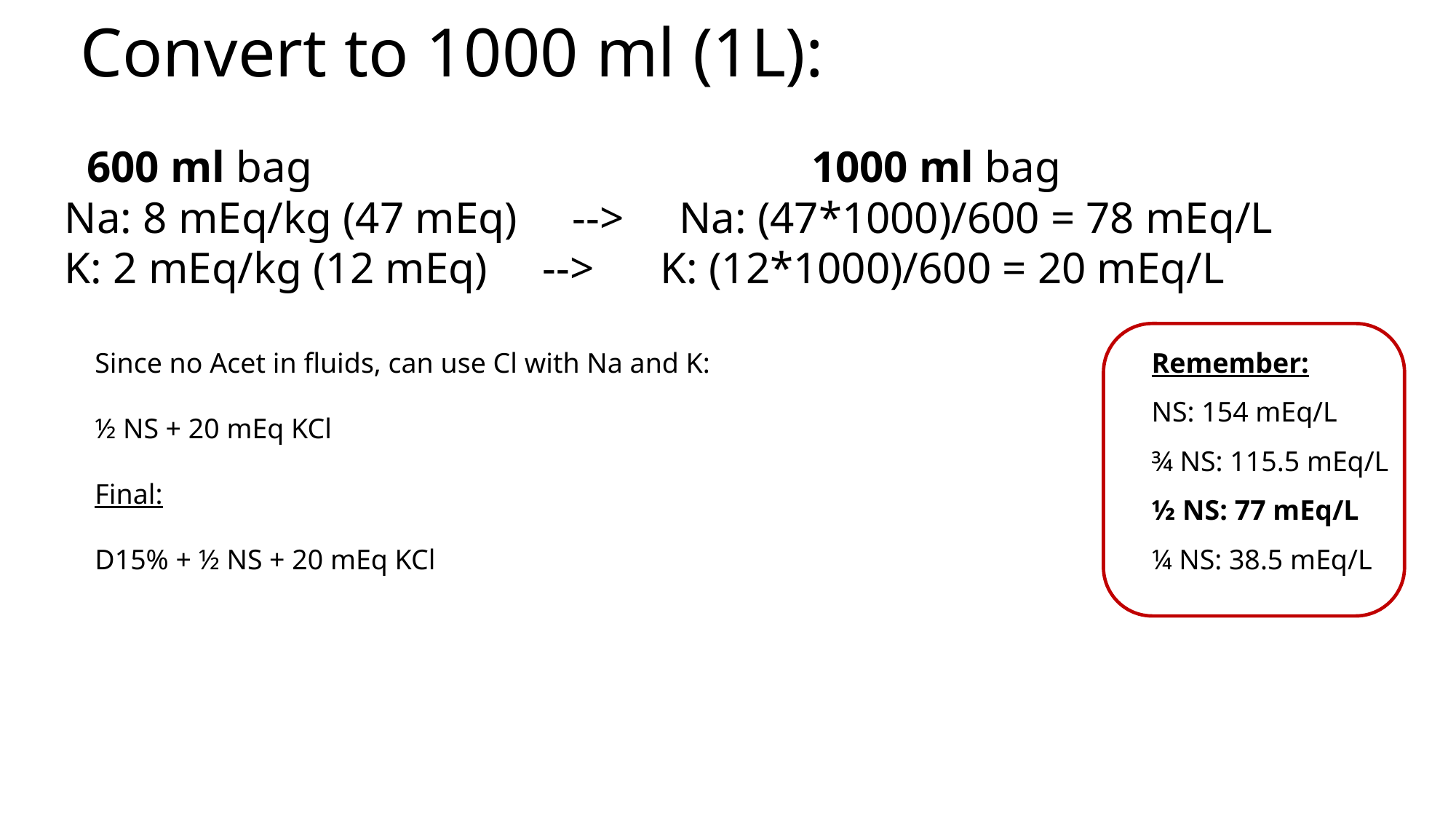

# Convert to 1000 ml (1L):
 600 ml bag 1000 ml bag
Na: 8 mEq/kg (47 mEq) --> Na: (47*1000)/600 = 78 mEq/L
K: 2 mEq/kg (12 mEq) --> K: (12*1000)/600 = 20 mEq/L
Since no Acet in fluids, can use Cl with Na and K:
½ NS + 20 mEq KCl
Final:
D15% + ½ NS + 20 mEq KCl
Remember:
NS: 154 mEq/L
¾ NS: 115.5 mEq/L
½ NS: 77 mEq/L
¼ NS: 38.5 mEq/L

## Slide 28
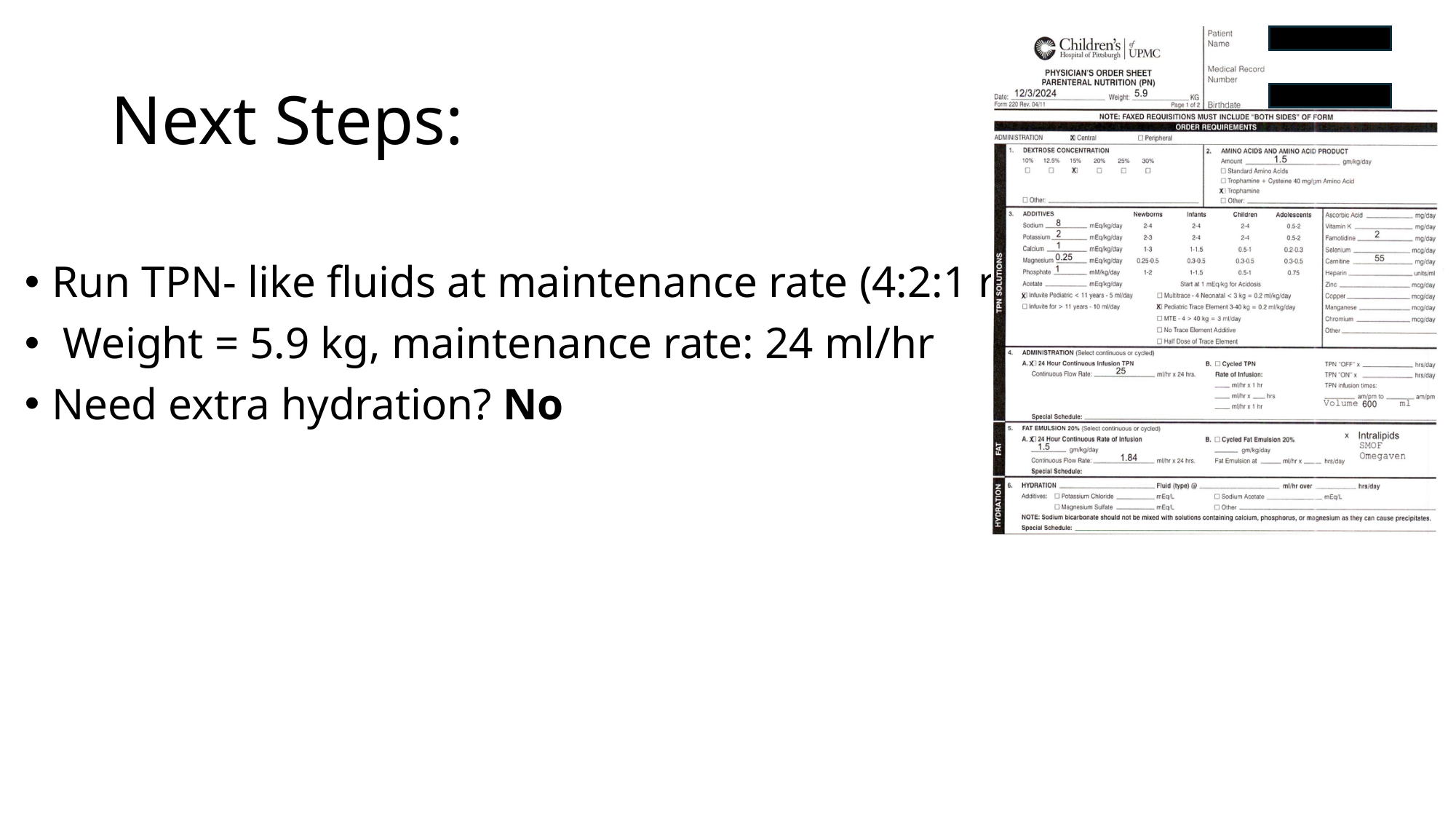

# Next Steps:
Run TPN- like fluids at maintenance rate (4:2:1 rule)
 Weight = 5.9 kg, maintenance rate: 24 ml/hr
Need extra hydration? No

## Slide 29
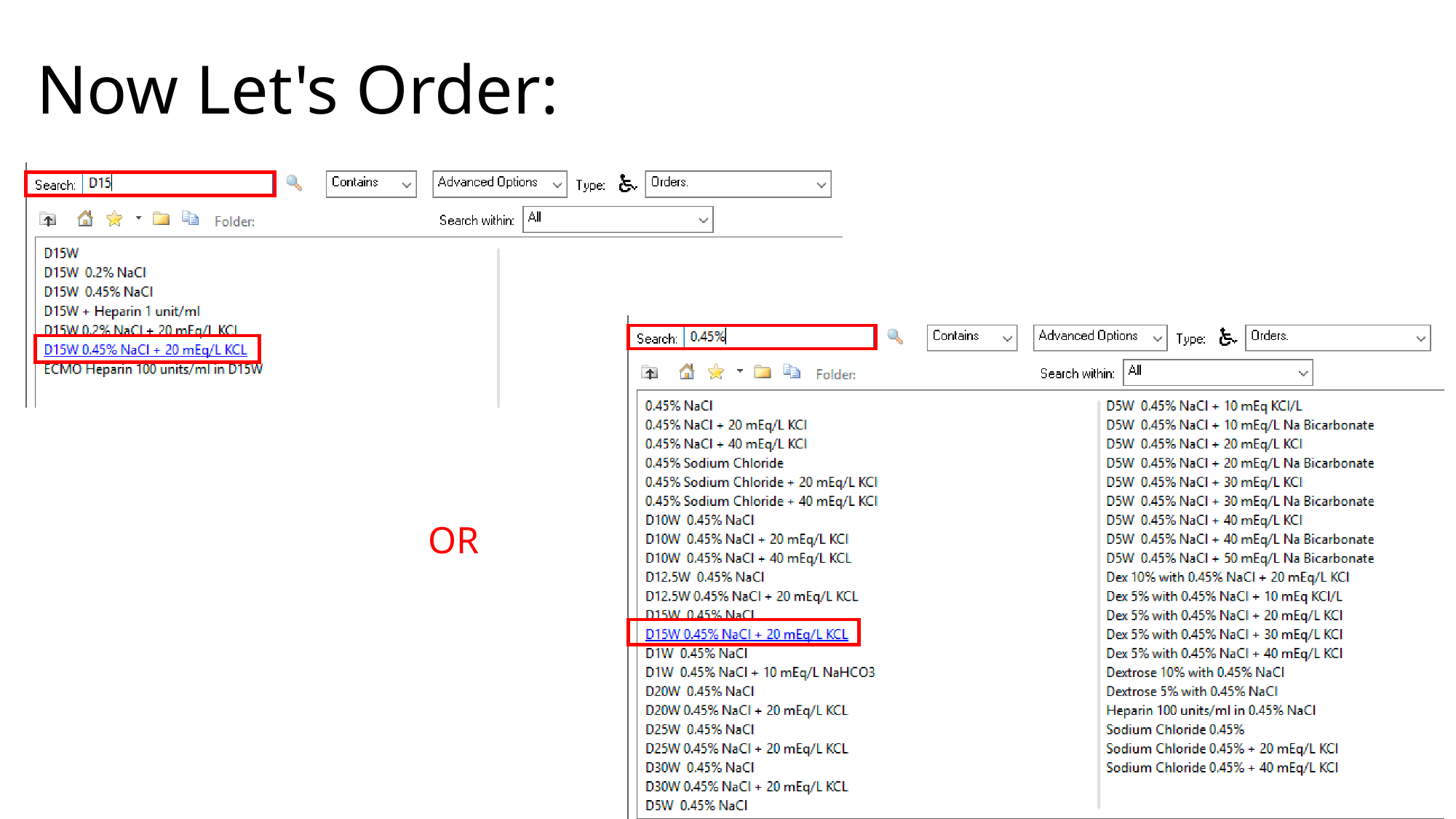

# Now Let's Order:
OR

## Slide 30
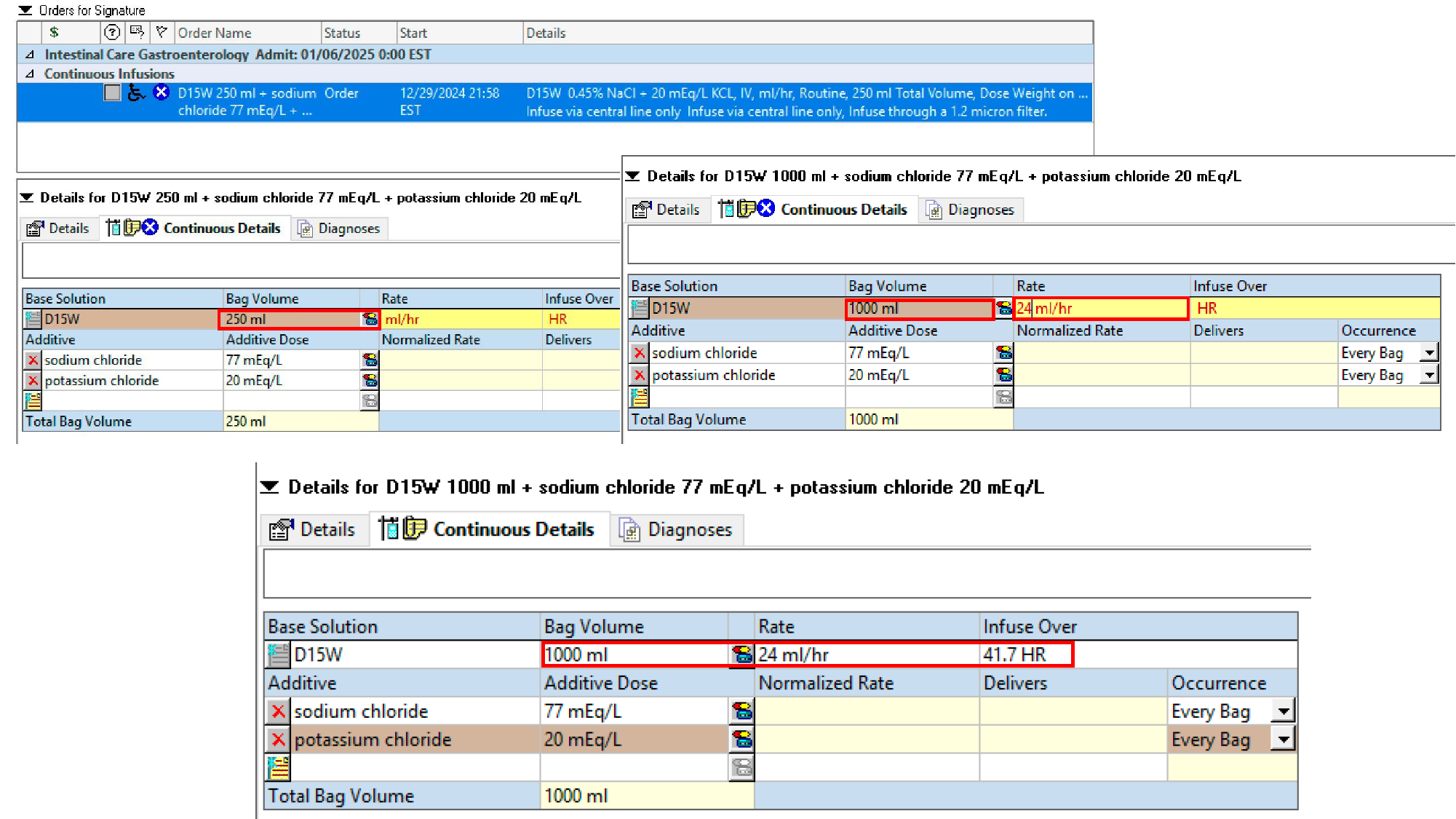

## Slide 31
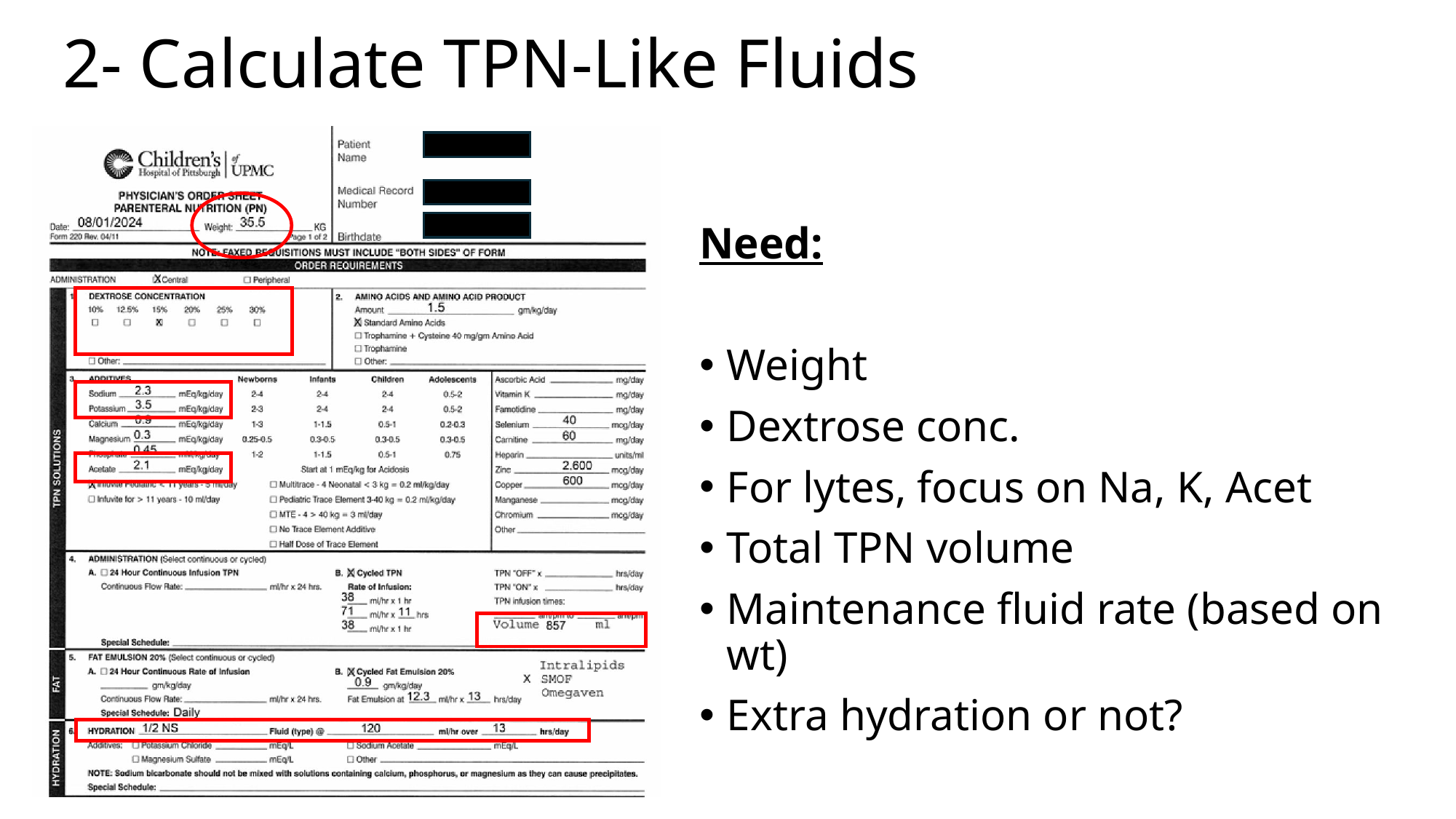

# 2- Calculate TPN-Like Fluids
Need:
Weight
Dextrose conc.
For lytes, focus on Na, K, Acet
Total TPN volume
Maintenance fluid rate (based on wt)
Extra hydration or not?

## Slide 32
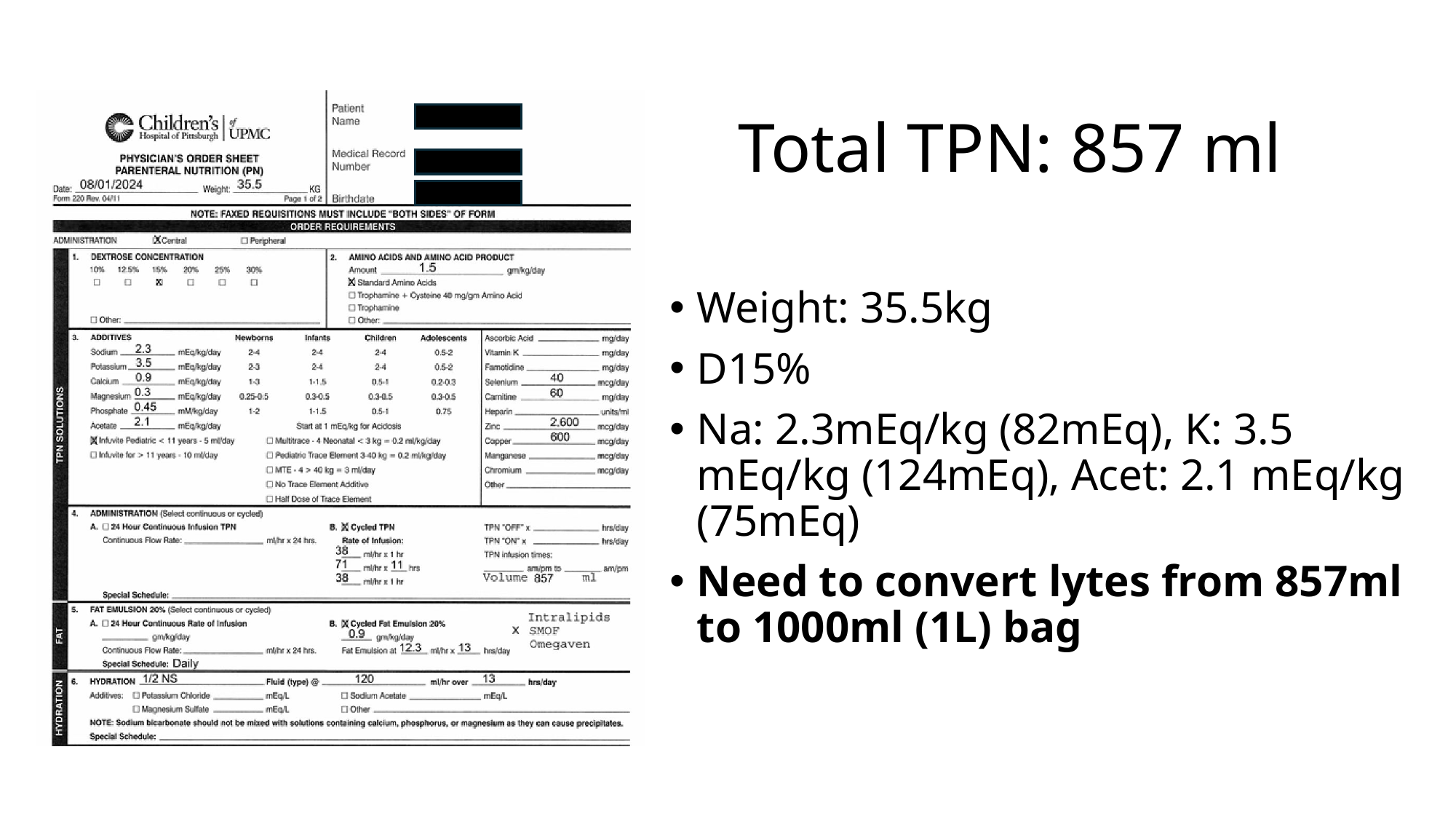

# Total TPN: 857 ml
Weight: 35.5kg
D15%
Na: 2.3mEq/kg (82mEq), K: 3.5 mEq/kg (124mEq), Acet: 2.1 mEq/kg (75mEq)
Need to convert lytes from 857ml to 1000ml (1L) bag

## Slide 33
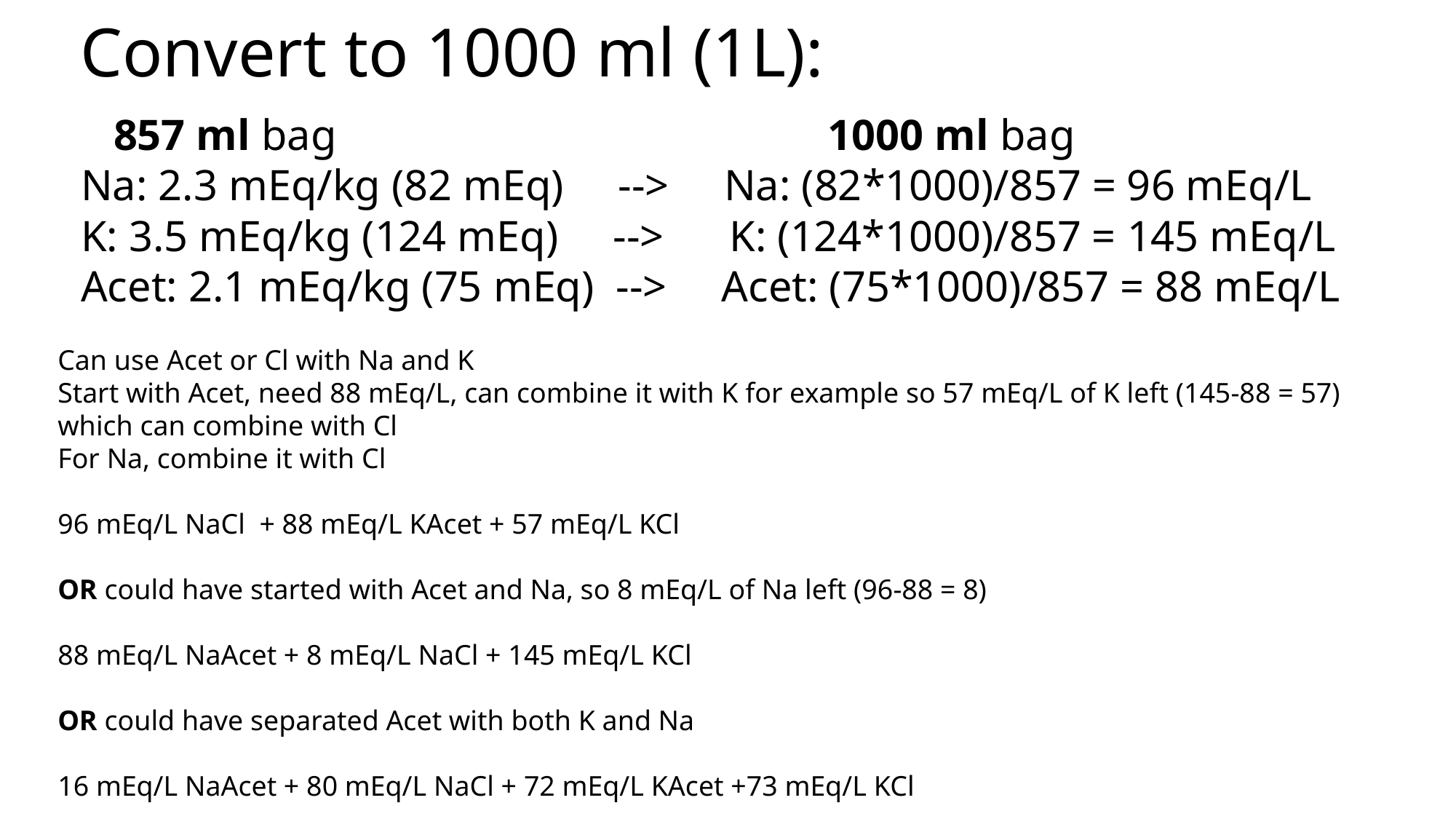

# Convert to 1000 ml (1L):
 857 ml bag 1000 ml bag
Na: 2.3 mEq/kg (82 mEq) --> Na: (82*1000)/857 = 96 mEq/L
K: 3.5 mEq/kg (124 mEq) --> K: (124*1000)/857 = 145 mEq/L
Acet: 2.1 mEq/kg (75 mEq) --> Acet: (75*1000)/857 = 88 mEq/L
Can use Acet or Cl with Na and K
Start with Acet, need 88 mEq/L, can combine it with K for example so 57 mEq/L of K left (145-88 = 57) which can combine with Cl
For Na, combine it with Cl
96 mEq/L NaCl  + 88 mEq/L KAcet + 57 mEq/L KCl
OR could have started with Acet and Na, so 8 mEq/L of Na left (96-88 = 8)
88 mEq/L NaAcet + 8 mEq/L NaCl + 145 mEq/L KCl
OR could have separated Acet with both K and Na
16 mEq/L NaAcet + 80 mEq/L NaCl + 72 mEq/L KAcet +73 mEq/L KCl

## Slide 34
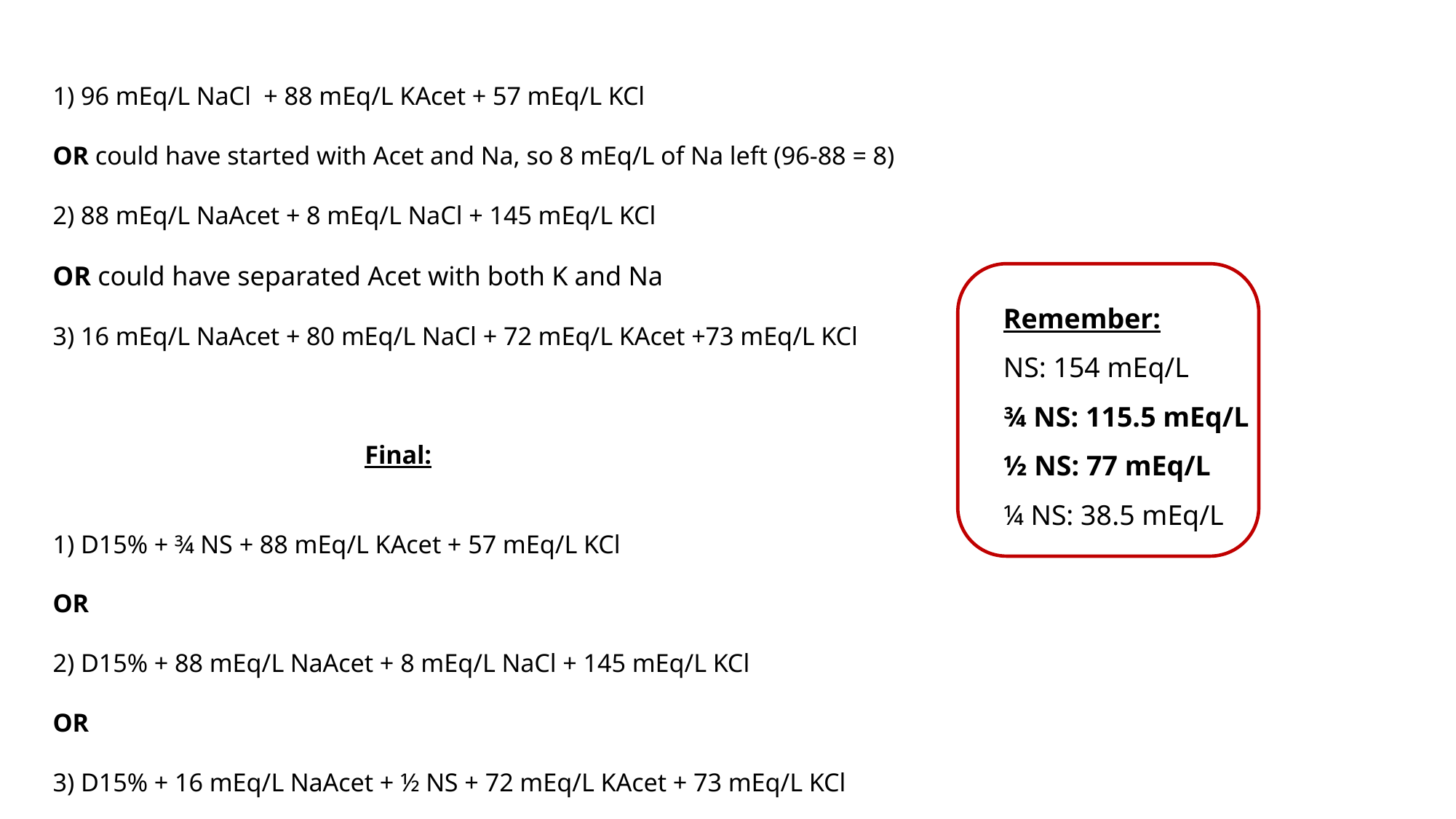

1) 96 mEq/L NaCl  + 88 mEq/L KAcet + 57 mEq/L KCl
OR could have started with Acet and Na, so 8 mEq/L of Na left (96-88 = 8)
2) 88 mEq/L NaAcet + 8 mEq/L NaCl + 145 mEq/L KCl
OR could have separated Acet with both K and Na
3) 16 mEq/L NaAcet + 80 mEq/L NaCl + 72 mEq/L KAcet +73 mEq/L KCl
 Final:
1) D15% + ¾ NS + 88 mEq/L KAcet + 57 mEq/L KCl
OR
2) D15% + 88 mEq/L NaAcet + 8 mEq/L NaCl + 145 mEq/L KCl
OR
3) D15% + 16 mEq/L NaAcet + ½ NS + 72 mEq/L KAcet + 73 mEq/L KCl
Remember:
NS: 154 mEq/L
¾ NS: 115.5 mEq/L
½ NS: 77 mEq/L
¼ NS: 38.5 mEq/L

## Slide 35
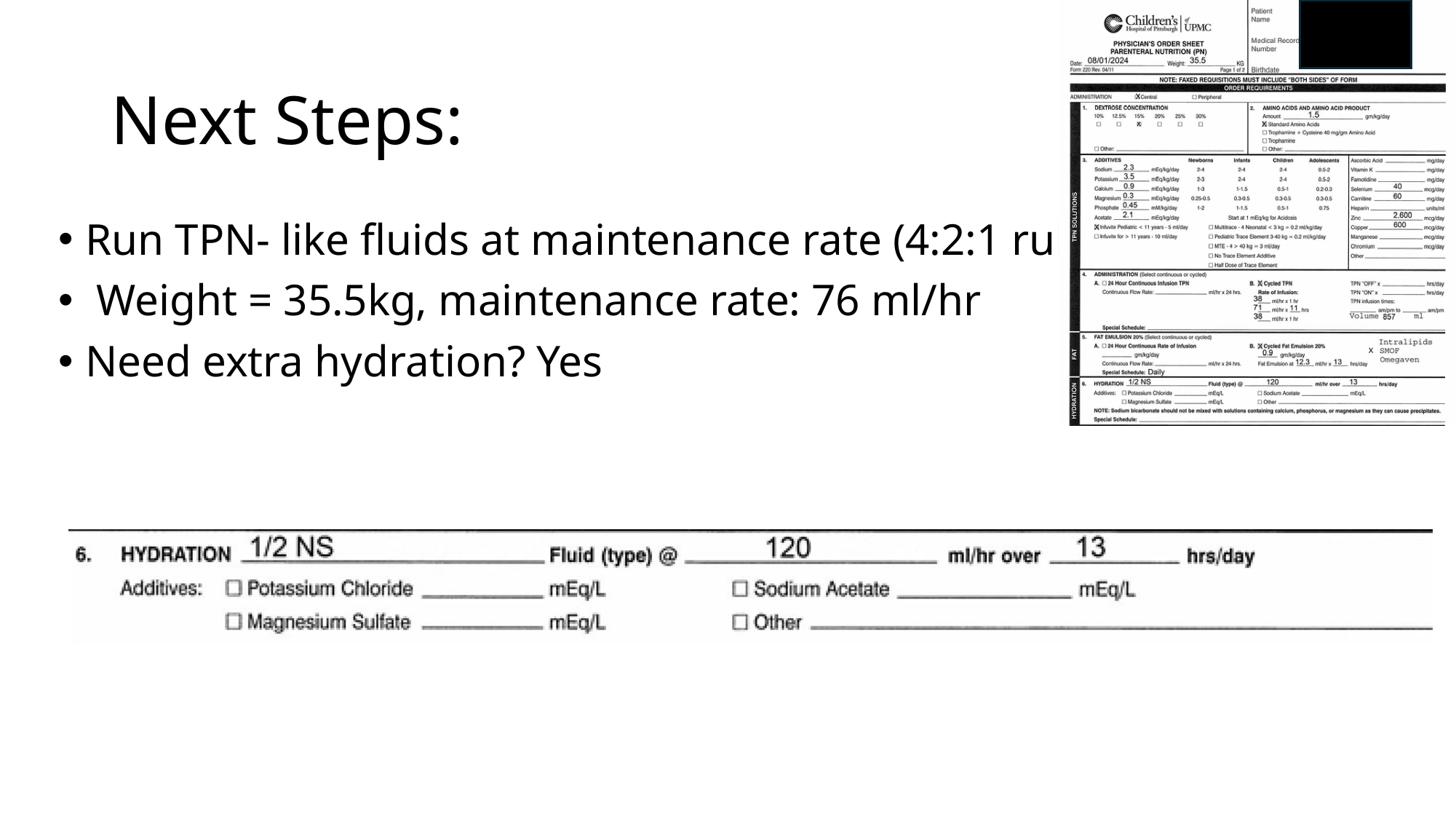

# Next Steps:
Run TPN- like fluids at maintenance rate (4:2:1 rule)
 Weight = 35.5kg, maintenance rate: 76 ml/hr
Need extra hydration? Yes

## Slide 36
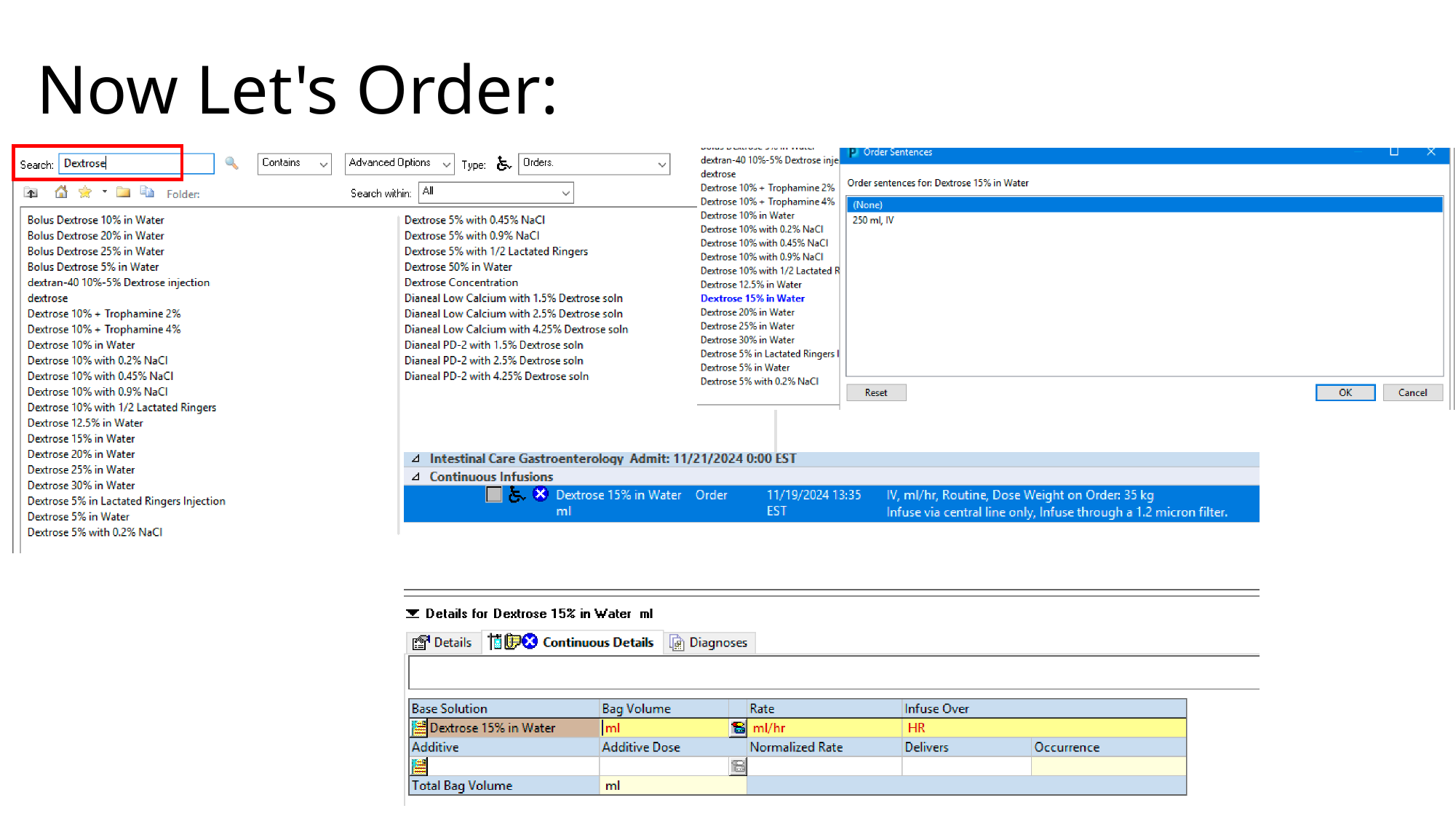

# Now Let's Order:

## Slide 37
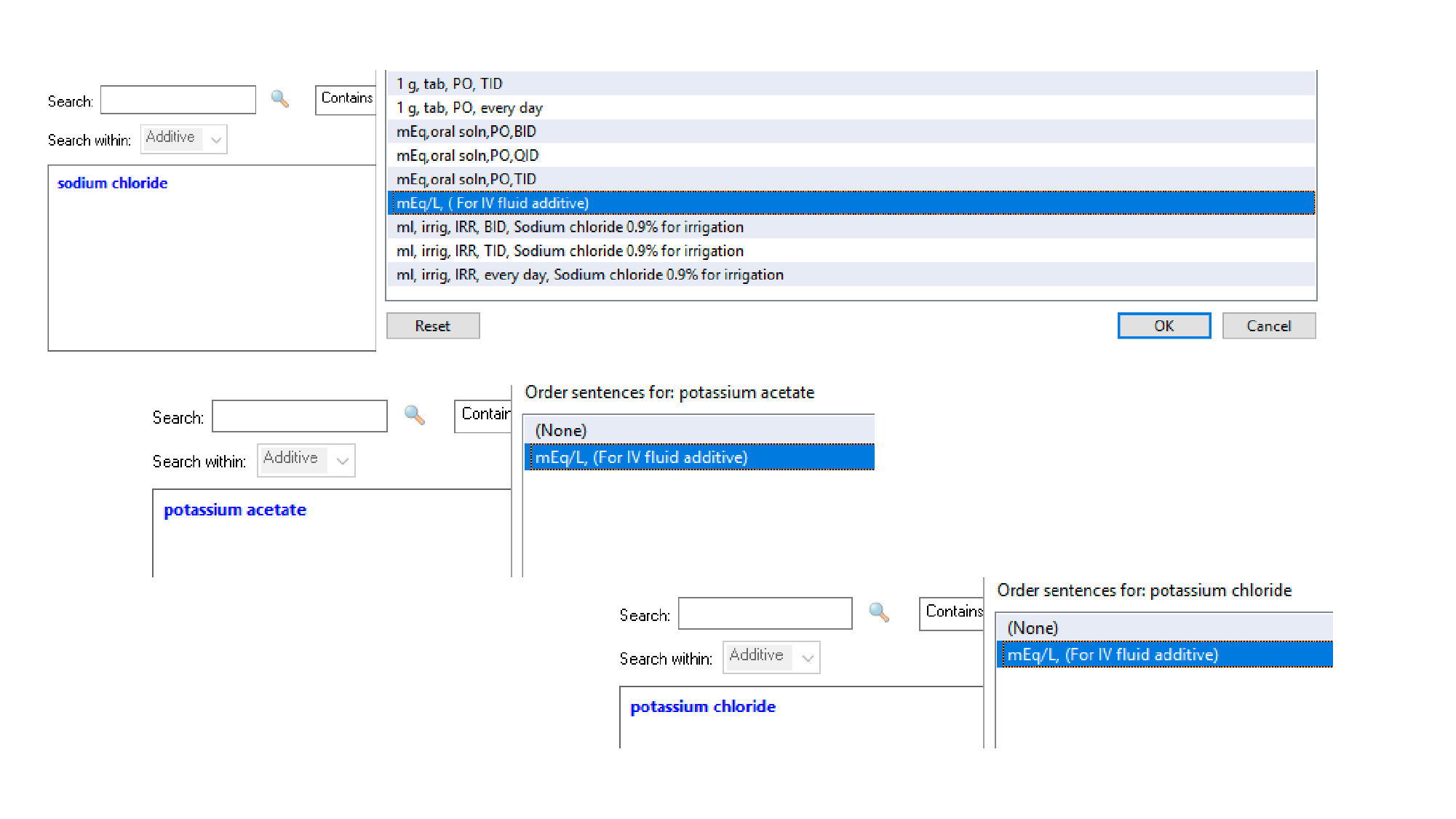

## Slide 38
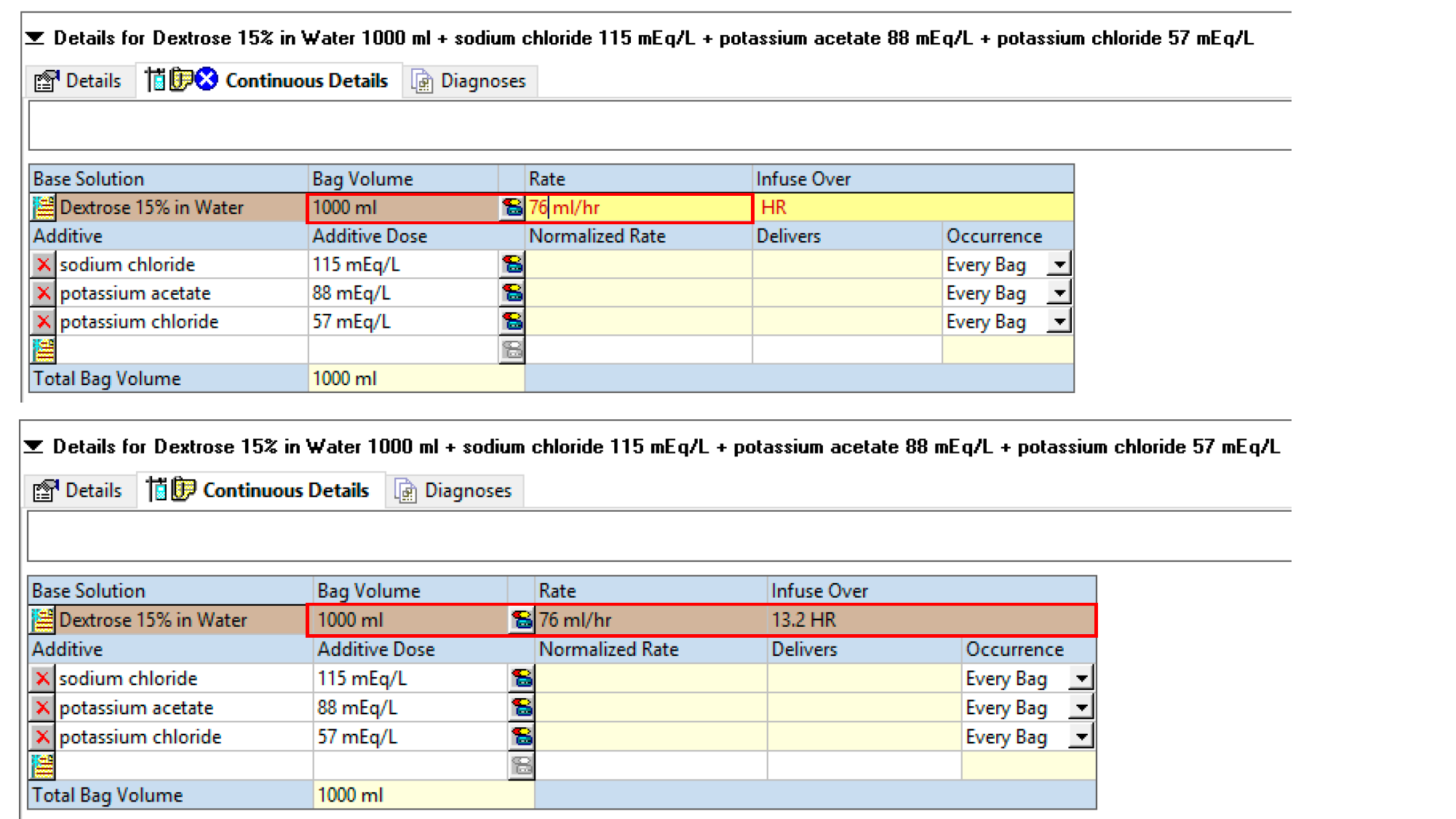

## Slide 39
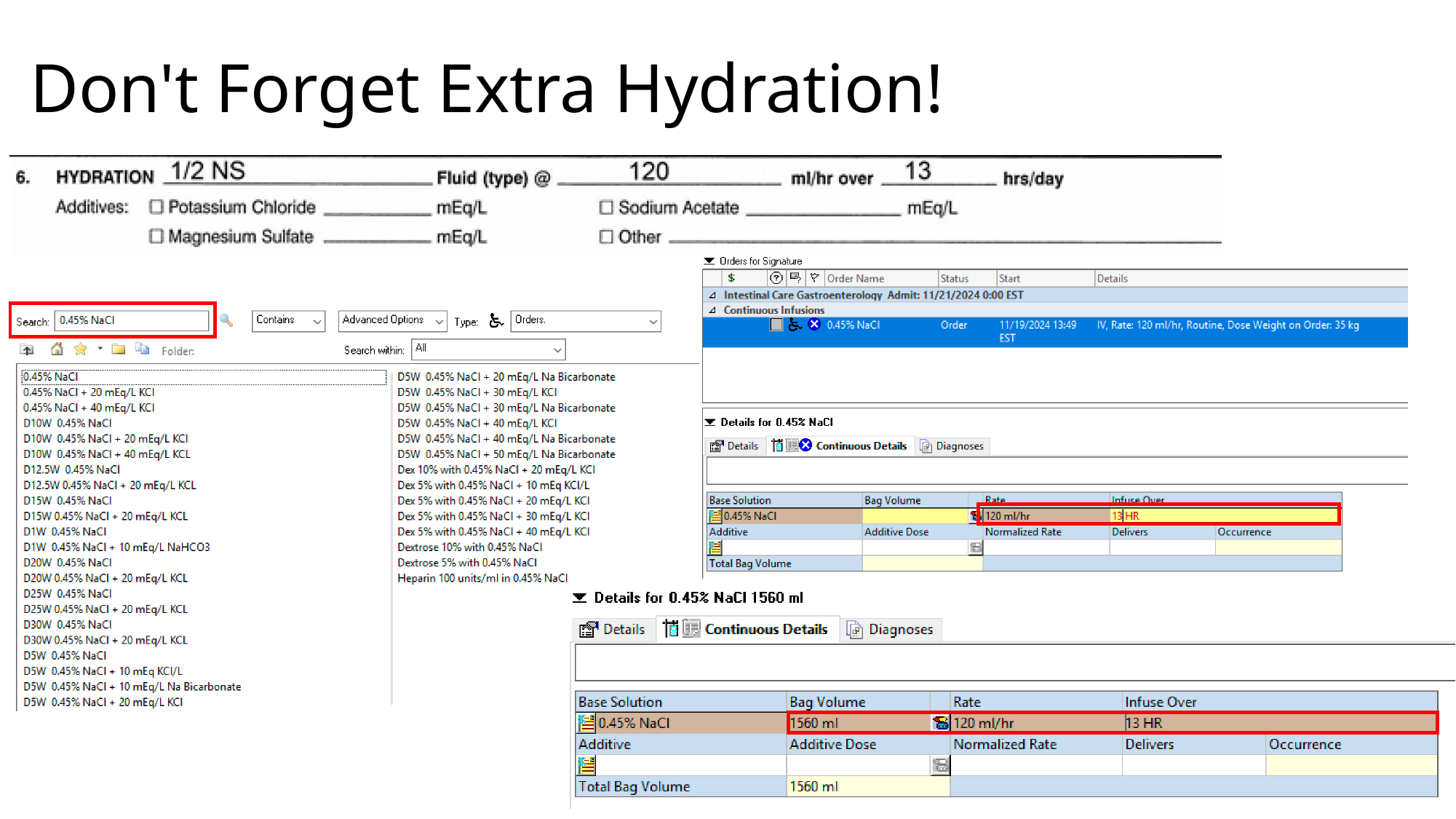

# Don't Forget Extra Hydration!

## Slide 40
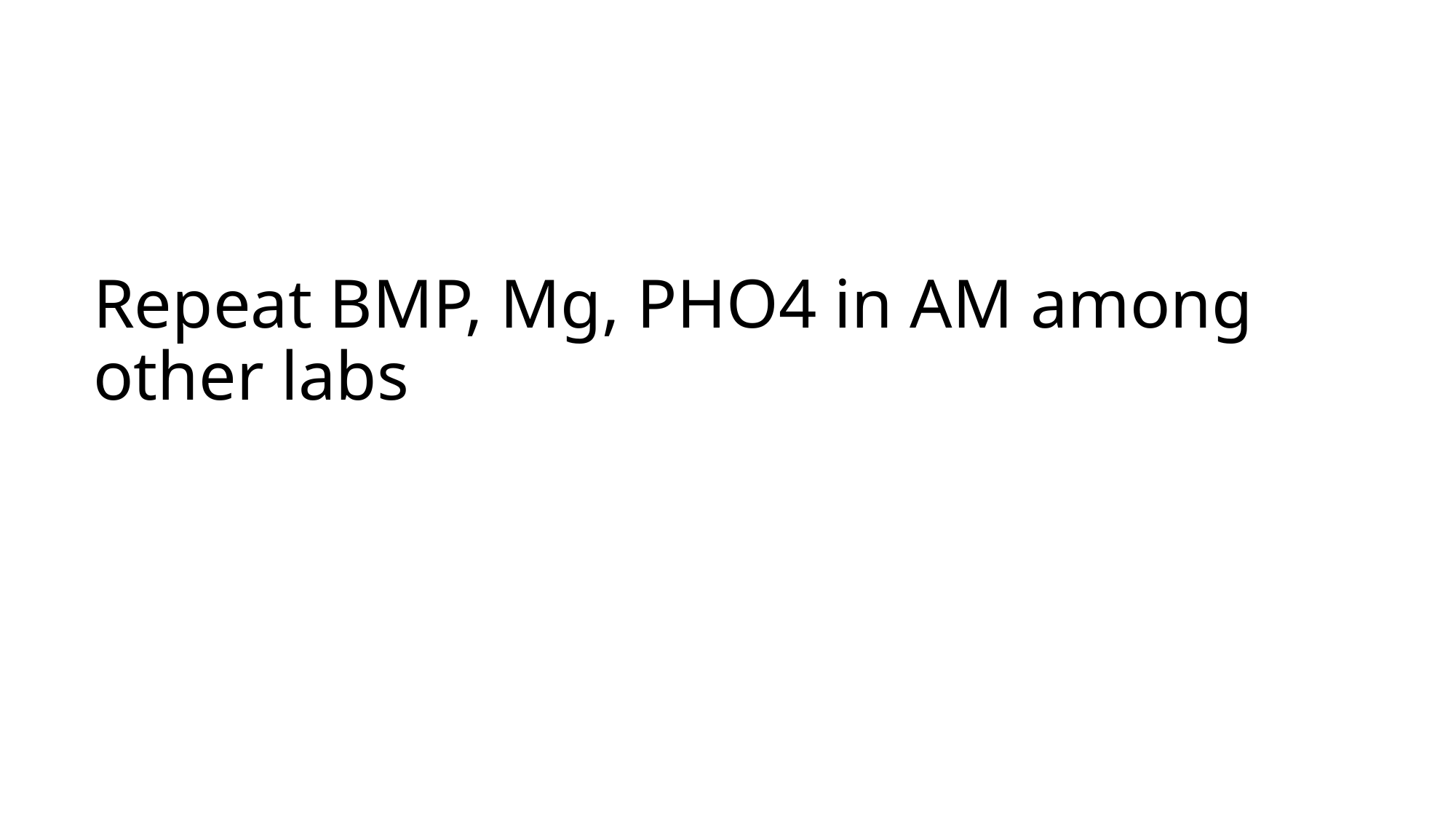

# Repeat BMP, Mg, PHO4 in AM among other labs

## Slide 41
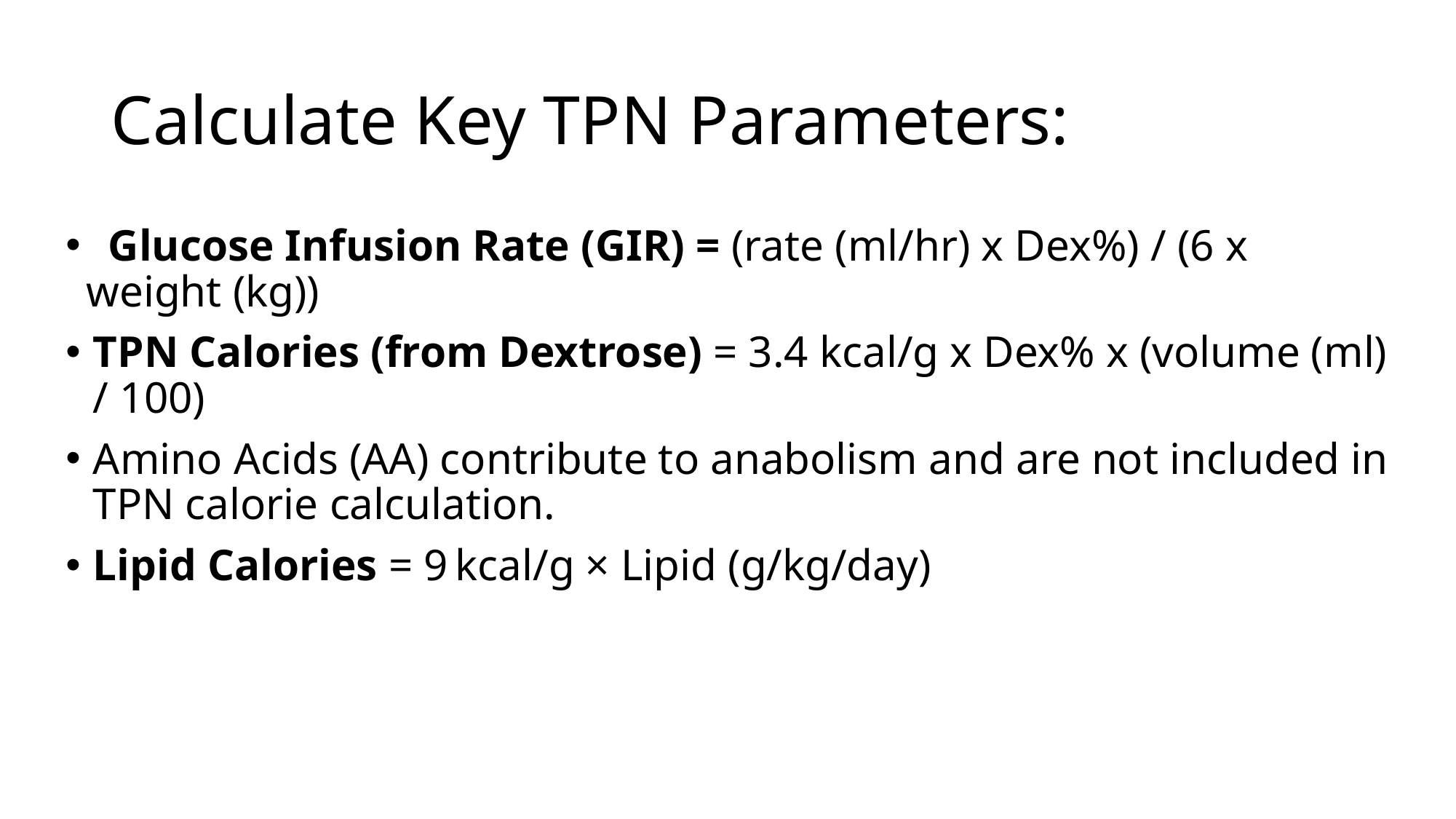

# Calculate Key TPN Parameters:
 Glucose Infusion Rate (GIR) = (rate (ml/hr) x Dex%) / (6 x weight (kg))
TPN Calories (from Dextrose) = 3.4 kcal/g x Dex% x (volume (ml) / 100)
Amino Acids (AA) contribute to anabolism and are not included in TPN calorie calculation.
Lipid Calories = 9 kcal/g × Lipid (g/kg/day)
