# Supplementary material for: Addressing gaps in pediatric resident education on the management of intestinal failure in the United States: Creation and implementation of a targeted curriculum
Source: Intest Fail. 2026 Apr 11;10:100368. doi: 10.1016/j.intf.2026.100368 (PMC13092194; doi:10.1016/j.intf.2026.100368)
Supplement: Supplementary file 4 — Supplementary material [file mmc4.docx]

1. **Order Home TPN**

1. **Find TPN Sheets**:
   1. Navigate to: Clinical Notes → Orders → Parenteral Nutrition
   2. Select the most recent TPN sheet.
2. **Add New Order**:
   1. Type "TPN" and select the TPN formula appropriate for the patient’s age.
3. For "full trace" or "cholestasis trace," check the bottom of Section 3 on the TPN sheets. If neither is specified, choose one (as you will specify it in Step 5).
4. **Adjust TPN Components**:
   1. Modify the following based on the TPN sheets:
      1. Dextrose (Dex%)
      2. Amino Acids (AA)
      3. Electrolytes (Lytes)
5. **Add Vitamins and Additives**:
   1. Click on the small yellow notepad icon on the bottom left to add vitamins, trace elements, and other additives.
   2. After selecting the item, choose “For PN Orders”.
6. **Set Total Volume and Cycled TPN**:
   1. Enter Total Bag Volume.
   2. For cycled TPN, enter “cycled over # hours” in the Rate section (first row).
      1. A warning box will appear—click “Yes” to proceed with free-text rate.
   3. In the Infusion Instructions box, specify:
      1. Cycled over # hours. Rate for the first and last hours and rate for the middle hours

Example: “Cycled over 13 hours, 36 ml/hr for the first and last hours, 71 ml/hr for the 11 hours in between.”

1. **Additional Details**:
   1. Select TPN to be delivered via central line, adjust the TPN duration accordingly in the Details section (top right).
2. **Order Lipids and Hydration**:
   1. Order lipids and extra hydration separately as per TPN sheets.
3. **Timing**:
   1. TPN and intralipids should be ordered daily before 2 PM for adequate pharmacist preparation.

### **2. Calculate Key TPN Parameters**

1. **Glucose Infusion Rate (GIR) =** (rate (ml/hr) x Dex%) / (6 x weight (kg))
2. **TPN Calories (from Dextrose)** = 3.4 kcal/g x Dex% x (volume (ml) / 100)

Amino Acids (AA) contribute to anabolism and are not included in TPN calorie calculation.

1. **Lipid Calories** = 9 kcal/g × Lipid (g/kg/day)

### **3. Order TPN-like Fluids (for overnight admissions)**

1. **Check Initial Lytes**:
   1. Review electrolyte levels obtained in the ED to assess the need for electrolyte replacement before starting TPN-like IVF.
2. **Find Home TPN Sheets**:
   1. Follow the steps outlined above
   2. Collect this data: weight (kg), total bag volume (ml), Dex%, electrolytes (only Na, K, Acetate), and extra hydration fluids (if present).
3. **Assess Access Type (Central vs Peripheral)**:
   1. Peripheral access: max Dex% is 12.5%.
4. **Adjust for Dextrose Drop**:
   1. If a significant drop in Dextrose occurs when switching from home TPN to peripheral access, consider:
      1. Enteral feeds, if appropriate (check NPO status).
      2. If patient is NPO or an infant, monitor blood glucose levels frequently and determine if a D10 bolus is needed (for hypoglycemia) or if additional dextrose-containing fluids need to be infused through another line.
5. **Convert Electrolytes**:
6. Calculate electrolytes (Na, K, Acetate) for a **1L** bag based on the patient's home TPN values:
7. For example: If the total bag volume is 600 ml and Na is 8 mEq/kg/day for a 6 kg patient: There are 48 mEq Na in that 600ml bag, then cross multiply to find out how much Na is in 1L bag: ((48 x1000) / 600) which equals 80 mEq Na in 1L bag.
8. ii. Repeat above steps for K and Acetate
9. **Combining Electrolytes**:
   1. Combine Acetate with Na or K or both until the Acetate requirement is met, then combine the remaining Na and K with Chloride (Cl).
10. **Order Appropriate Fluids**:
    1. Use the following guidelines for fluids:
       1. **Normal Saline (NS)**: 154 mEq/L
       2. **3/4 NS**: 115.5 mEq/L
       3. **1/2 NS**: 77 mEq/L
       4. **1/4 NS**: 38.5 mEq/L
    2. Dextrose: It's acceptable to round the Dex% to the nearest 5% (e.g., D18.5% can be ordered as D20%).
    3. Electrolytes: Round electrolytes (e.g., KCl) to the nearest 5 mEq/L (e.g., 17 mEq/L KCl can be rounded to 20 mEq/L).
    4. Cerner may suggest fluid combinations, such as D15% + 1/2 NS + 20 KCl.
    5. If none of the suggested combinations work, add components separately.
       1. Start the order with either the Dextrose or saline concentration, then add the remaining components.
11. **Order for 1L Bag**:
    1. Ensure you write the order for a **1000 ml** bag and set the **maintenance rate** (use 4:2:1 rule).
12. **Order Extra Hydration**:
    1. Order any extra hydration fluids separately, as specified in the TPN sheets.
13. Order relevant labs: BMP, Mg, PHO4 in AM

**4. Central Line Complications**

***A. Central Line-Associated Blood Stream Infections (CLABSI)***

- **Signs of CLABSI**: Fever (≥ 38.0°C / 100.4°F) + central line + variable clinical appearance. Also consider CLABSI if the patient is not acting his/her normal self.
- **Action**:
  - Obtain blood cultures from all lumens of the CVC and a peripheral culture.
  - Evaluate for other infections (labs, urine testing, viral testing, CXR).
  - Fluid resuscitation: If a ≥60 mL/kg NS bolus is required, PICU admission may be necessary.
  - Start IV antibiotics: Cefepime and Vancomycin (check for any previous drug sensitivities or renal disease, obtain Vanco trough prior to 4th dose).
  - Consult Infectious Disease if history of multidrug-resistant bacteria.
  - Consider antifungals (e.g., Micafungin) if pseudo hyphae on culture or blood smear, in the ill-appearing child or if clinical concern persists despite adequate antibacterial coverage.
  - Repeat central cultures every 24 hours until bacteremia clears.
  - Repeat peripheral culture every 24h only if previous culture returns positive.

#### **B. Line Malposition/Dislodgement**

- **Signs of Dislodgement**: Line malpositioned or line cuff exposed.
- **Action**:
  - Obtain CXR to confirm line position.
  - Central: Tip approximates at the cavoatrial junction (can remain central up to the brachiocephalic vein)
  - If dislodged, replace line via IR or Surgery (consult the team who placed the line).

#### **C. Line Breakage**

- **Action**:
  - The IV team will assess in the ED (as silicone lines are repairable)
  - If unrepairable, admit to ICARE for line replacement.
  - “Consult Vascular Access” order set, not “Consult IR” (allows IV team to facilitate the scheduling with IR)
  - If the line was placed by pediatric surgery, consult surgery for further management.
  - NPO status: Order TPN-like IVF, CBC, and coags in AM.
  - Hold Lovenox/Xarelto.

#### **D. Line Occlusion**

- **Types**:
  - Partial: Can flush but cannot aspirate.
  - Complete: Cannot flush, infuse, or aspirate.
- **Action**:

1. Rule out external mechanical causes (kinking, clamping).
2. Attempt positional changes (e.g., raise/lower arm, stand up, sit, roll onto side).
3. Obtain CXR to check for malposition or kink.
4. Try NS flush (don't force it) for possible fibrin sheath.
5. If needed, administer fibrinolytic agents such as TPA:
6. Dosage: <10 kg: 0.5 mg, >10 kg: 1 mg
7. Dilute TPA with NaCl, leave in the lumen for 1-2 hours, then aspirate TPA and flush with NS.
8. If TPA fails x2, consider venous ultrasound
9. Primary team may later consider further imaging such as: CT/MR Venography, Venography (Fluoroscopy)
10. If thrombus is present, IR can dislodge thrombus at tip of CVC with a guidewire and/or rewire the line.
11. If thrombus is present, consider Heme consult to initiate LMWH for 6 weeks to 3 months.
