# Supplementary material for: Addressing gaps in pediatric resident education on the management of intestinal failure in the United States: Creation and implementation of a targeted curriculum
Source: Intest Fail. 2026 Apr 11;10:100368. doi: 10.1016/j.intf.2026.100368 (PMC13092194; doi:10.1016/j.intf.2026.100368)
Supplement: Supplementary file 5 — Supplementary material [file mmc5.pdf]

Default Question Block

Q1. Have you rotated on ICARE (day or night) prior to taking this post-intervention survey?

- Yes
- No
- I cannot recall

Q2. Did you attend the in-person lecture on the "Intestinal Rehab Residency Curriculum" presented by Sirine Belaid, 3rd year Pediatric GI fellow, on 1/2/25?

- Yes
- No
- I cannot recall

Q3. Did you attend the TPN workshops led by Sirine Belaid, 3rd year Pediatric GI fellow, focused on practicing the calculation and ordering of TPN-like IV fluids and home TPN?

- ◯ Yes
- ◯ No
- ◯ I cannot recall

Q4. Rate from 1 to 10 the following items based on your **level of confidence** in performing the following tasks **individually** when managing ICARE patients, with 1 being not confident and 10 being very confident.

ICARE stands for Intestinal Care and Rehabilitation Center
TPN stands for Total Parenteral Nutrition
CLABSI stands for Central Line-Associated Bloodstream Infection

Click to write Label 1

	0	1	2	3	4	5	6	7	8	9	10
Finding TPN sheets on Cerner											
Calculate TPN-like fluids											
Order TPN-like fluids on Cerner											
Order home TPN											
Identify proper antibiotics for CLABSI rule out											
Order proper dose of antibiotics for CLABSI rule out											
Order proper interval of antibiotics for CLABSI rule out											

Click to write Label 1

	0	1	2	3	4	5	6	7	8	9	10	
Order appropriate blood cultures required for CLABSI rule out												
Identify proper steps when central access is lost												
Manage poor blood return from central line												
Identify signs of hypovolemic shock												
Manage hypovolemic shock												
Identify signs of septic shock												

Click to write Label 1

	0	1	2	3	4	5	6	7	8	9	10
Manage septic shock											
Identify appropriate steps for feeding intolerance (with otherwise reassuring exam)											
Identify signs of D-lactic acidosis											
Identify signs of bacterial overgrowth											
Manage suspected bacterial overgrowth											

Click to write Label 1

	0	1	2	3	4	5	6	7	8	9	10
Effectively understand their remaining bowel anatomy											
Identify barrier cream for enteral/stoma care											
Order barrier cream for enteral/stoma care											
Contact ostomy nurse to assist me in patient care											

Click to write Label 1

	0	1	2	3	4	5	6	7	8	9	10
Use educational resources (such as Lexicomp, Uptodate) to assist me in patient care											

Block 1

Q5. After attending the in-person lecture on the "Intestinal Rehab (IR) Residency Curriculum" or viewing the recorded session, how did the lecture impact your **confidence** in independently managing ICARE patients?

- I **did not attend** the lecture or watch the recorded session
- **Not at all confident** – I do not feel prepared to manage ICARE patients independently
- **Slightly confident** – I gained some understanding, but still need significant guidance
- **Moderately confident** – I feel more capable, but would still prefer supervision

○ **Very confident** – I feel well-prepared to manage ICARE patients with **minimal support**

○ **Extremely confident** – I feel fully prepared to manage ICARE patients **independently**

Q6. What **feedback or suggestions** do you have to improve the in-person lecture and help increase your confidence in managing ICARE patients?

Q7. After attending the TPN workshops, how did the sessions impact your **confidence** in independently calculating and ordering TPN-like IV fluids and home TPN for ICARE patients?

○ I **did not attend** the workshops

○ **Not at all confident** – I do not feel prepared to calculate or order TPN-like IV fluids independently

- ○ **Slightly confident** – I gained some understanding, but still need significant guidance
- ○ **Moderately confident** – I feel more capable, but would still prefer supervision
- ○ **Very confident** – I feel well-prepared to calculate and order TPN like IVF and home TPN with **minimal support**
- ○ **Extremely confident** – I feel fully prepared to calculate and order TPN like IVF and home TPN **independently**

Q8. What **feedback or suggestions** do you have to improve the TPN workshops and increase your confidence in calculating and ordering TPN-like IV fluids and home TPN for ICARE patients?

Q9. Overall, I believe it is of **educational value** for pediatric residents to manage ICARE patients.

- ○ Strongly disagree
- ○ Somewhat disagree

- ○ Neither agree nor disagree
- ○ Somewhat agree
- ○ Strongly agree

Powered by Qualtrics
